# Safety and Adherence to Intermittent Pre-Exposure Prophylaxis (PrEP) for HIV-1 in African Men Who Have Sex with Men and Female Sex Workers

**DOI:** 10.1371/journal.pone.0033103

**Published:** 2012-04-12

**Authors:** Gaudensia Mutua, Eduard Sanders, Peter Mugo, Omu Anzala, Jessica E. Haberer, David Bangsberg, Burc Barin, James F. Rooney, David Mark, Paramesh Chetty, Patricia Fast, Frances H. Priddy

**Affiliations:** 1 Kenya AIDS Vaccine Initiative, University of Nairobi, Nairobi, Kenya; 2 Kenya Medical Research Institute, Kilifi, Kenya; 3 Nuffield Department of Medicine, University of Oxford, Headington, United Kingdom; 4 Massachusetts General Hospital Center for Global Health, Boston, Massachusetts, United States of America; 5 The EMMES Corporation, Rockville, Maryland, United States of America; 6 Gilead, Foster City, California, United States of America; 7 International AIDS Vaccine Initiative, New York, New York, United States of America; 8 International AIDS Vaccine Initiative, Johannesburg, South Africa; University of Cape Town, South Africa

## Abstract

**Background:**

Little is known about safety of and adherence to intermittent HIV PrEP regimens, which may be more feasible than daily dosing in some settings. We present safety and adherence data from the first trial of an intermittent PrEP regimen among Kenyan men who have sex with men (MSM) and female sex workers (FSW).

**Methods/Principal Findings:**

MSM and FSW were randomized to daily oral FTC/TDF or placebo, or intermittent (Monday, Friday and within 2 hours after sex, not to exceed one dose per day) oral FTC/TDF or placebo in a 2∶1∶2∶1 ratio; volunteers were followed monthly for 4 months. Adherence was assessed with the medication event monitoring system (MEMS). Sexual activity data were collected via daily text message (SMS) queries and timeline followback interviews with a one-month recall period. Sixty-seven men and 5 women were randomized into the study. Safety was similar among all groups. Median MEMS adherence rates were 83% [IQR: 63–92] for daily dosing and 55% [IQR:28–78] for fixed intermittent dosing (p = 0.003), while adherence to any post-coital doses was 26% [IQR:14–50]. SMS response rates were low, which may have impaired measurement of post-coital dosing adherence. Acceptability of PrEP was high, regardless of dosing regimen.

**Conclusions/Significance:**

Adherence to intermittent dosing regimens, fixed doses, and in particular coitally-dependent doses, may be more difficult than adherence to daily dosing. However, intermittent dosing may still be appropriate for PrEP if intracellular drug levels, which correlate with prevention of HIV acquisition, can be attained with less than daily dosing and if barriers to adherence can be addressed. Additional drug level data, qualitative data on adherence barriers, and better methods to measure sexual activity are necessary to determine whether adherence to post-coital PrEP could be comparable to more standard regimens.

**Trial Registration:**

ClinicalTrials.gov NCT00971230

## Introduction

Recent studies indicate the widespread presence of MSM groups in Africa with high rates of HIV infection, HIV risk behavior and linkages to heterosexual networks [1]. Heterosexual transmission of HIV remains the predominant mode of transmission in Kenya, however up to 15% of all new infections are estimated to be directly attributable to men who have sex with men (MSM) [2]. Recently, the use of oral antiretroviral pre-exposure prophylaxis (PrEP) was shown to be effective in preventing HIV in MSM: the iPrEX study of approximately 2500 MSM and transgender women demonstrated a 44% reduction in the incidence of HIV following daily use of the fixed dose combination pill, Truvada, containing emtricitabine and tenofovir disoproxil fumarate (FTC/TDF) [3]. Efficacy correlated closely with drug adherence and drug levels. Pill use on 90% or more of days was associated with 73% efficacy, while detectable drug levels were associated with 92% efficacy. Early in 2011, the US Centers for Disease Control published interim guidance for the use of PrEP in MSM [4]. PrEP was also shown to be effective in other at-risk populations. Two studies of daily PrEP with either FTC/TDF or TDF alone in HIV serodiscordant couples and in young men and women in sub-Saharan Africa found that daily FTC/TDF or TDF alone reduced HIV incidence by 62–73% (Baeten J, Celum C. (2011) Antiretroviral Pre-Exposure Prophylaxis for HIV-1 prevention among heterosexual African men and women: the Partners PrEP Study [Abstract MOAX0106]. 6th IAS Conference on HIV Pathogenesis, Treatment and Prevention; 17–20 July 2011; Rome, Italy. Available: http://pag.ias2011.org/flash.aspx?pid=886. Accessed 14 March 2012. Thigpen MC, Kebaabetswe PM, Smith DK, Segolodi TM, Soud FA, et al. Daily oral antiretroviral use for the prevention of HIV infection in heterosexually active young adults in Botswana: results from the TDF2 study. [Abstract WELBC01]. 6th IAS Conference on HIV Pathogenesis, Treatment and Prevention; 17–20 July 2011; Rome, Italy. Available: http://pag.ias2011.org/abstracts.aspx?aid=4631. Accessed 14 March 2012.)

In contrast, a study of daily PrEP using FTC/TDF in at-risk women failed to find a reduced risk of HIV infection in the treatment group [5]. The possible biologic and behavioral reasons for lack of efficacy among women in this trial are under investigation. However, the prospect of keeping healthy individuals on lifelong chemoprophylaxis presents unique challenges in drug adherence and the potential for drug toxicity [6]. Critics of PrEP have also expressed concern about the cost of keeping a large population of HIV-uninfected people on chronic treatment, as well as the potential for HIV risk behavior compensation [7]. Both FTC and TDF are licensed for use by HIV-infected patients and have favorable safety profiles [8].

Reducing the frequency of administration by intermittent rather than daily use could mitigate concerns about toxicity and cost. The long intracellular half-lives of TDF and FTC, 40–60 hours, present this theoretical possibility [9], [10]. A macaque challenge study demonstrated that intermittent TDF/FTC PrEP offered comparable protection to daily PrEP against rectal transmission of SHIV [11]. However little is known about adherence to and acceptability of intermittent PrEP regimens in HIV at-risk groups. In particular, preference for and adherence to fixed doses (i.e. once weekly or twice weekly) versus post-coital dosing is unknown. Of interest for HIV vaccine development, PrEP may also have the potential to allow development of effective HIV-specific immune responses in people with repeated mucosal exposures who do not become infected. Several animal studies have shown higher rates of SIV-specific immune responses in PrEP treated animals that were protected from SIV infection or that controlled infection [12]–[19].

In this study, which was initiated and completed prior to the release of any PrEP efficacy results from studies cited above, we compared safety and drug adherence between daily and intermittent PrEP regimens of FTC/TDF. The intermittent PrEP regimen was defined as a twice-weekly dosing plus a coitally-dependent dose. We focused on MSM due to the high and ongoing burden of new HIV infections in this population, but included a limited number of FSW to help avoid community identification of the study as MSM-exclusive. In addition, we investigated the concept that suppression of viral replication by PrEP during repeated mucosal exposures may allow the development of effective anti-HIV immune responses.

## Methods

The protocol for this trial and supporting CONSORT checklist are available as supporting information; see Checklist S1 and Protocol S1.

### Participants

Two centers in Kenya (Kenya AIDS Vaccine Initiative [KAVI]-Kangemi in Nairobi, and Center for Geographic Medicine Research – Coast [GMRC]-KEMRI in Kilifi) were among the first in Africa to develop MSM cohort studies with very high HIV-1 incidence (∼9% per 100 person years observation), largely determined by unprotected receptive anal intercourse [20]. Kenya has now made interventions for MSM a key priority in their national HIV strategic plan [21] and centers in Nairobi and Kilifi are providing a comprehensive HIV prevention package to at-risk research volunteers, including regular HIV testing and risk reduction counseling, provision of male condoms and water-based lubricants, screening and treatment of sexually transmitted infections and referral for adult male circumcision for men practicing heterosexual sex. FSW are also followed at the centers. Volunteers were recruited from ongoing HIV prevention cohorts at the two centers. HIV-uninfected MSM and FSW aged 18–49 years who reported at least one of the following risk criteria in the past 3 months were enrolled in the study: current or previous STI, multiple episodes of unprotected vaginal or anal sex, or engaging in transactional sex. Volunteers with chronic hepatitis B infection (HBsAg-positive) or with creatinine clearance <80 mL/min and pregnant or lactating mothers were excluded due to study drug toxicity concerns. Women of childbearing potential were required to use a non-barrier form of contraception (hormonal contraception or intrauterine device) during study participation.

### Community stakeholder consultations

Both centers conduct regular community stakeholder consultations on new and ongoing research and have active community advisory boards. Study participation was discussed in small groups of 6–8 cohort volunteers at both centers. Community advisory boards at both centers reviewed study aims of the protocol and provided feedback on potential community concerns.

### Ethics

All volunteers gave written informed consent to participate in the study. The study was approved by the Kenyatta National Hospital Ethics Review Committee and the Kenya Medical Research Institute Ethics Review Committee.

### Study procedures

Volunteers were randomized to daily FTC/TDF or placebo, or intermittent (fixed dose on Mondays, Fridays and post-coital dose within 2 hours after sex, not to exceed 1 dose per day) FTC/TDF or placebo in a 2∶1∶2∶1 ratio, and followed monthly with standardized adherence and risk reduction counseling, HIV testing, and safety evaluation for 4 months. Adherence rate was assessed by the medication event monitoring system (MEMS), in which each opening of the pill bottle is recorded electronically, and by monthly self-report using a timeline followback interview calendar [22]–[24]. Briefly, the calendar is used to prompt recall of recent behavior and has been used successfully in other behavioral studies. Volunteers were given a key chain pill holder for portable dosing and were instructed to load the holder with a couple of pills if they felt that they could not carry the pill bottle with MEMS cap. Sexual activity data were collected via daily short message service (SMS) text message queries, as well as through the timeline followback face-to-face risk assessment with a monthly recall period. Volunteers were provided with free mobile phones, SIM cards and airtime credits, as well as condoms and water-based lubricants.

### Objectives

The objectives of the study were (1) to evaluate the safety of daily and intermittent dosing of FTC/TDF; (2) to compare the acceptability of and adherence to daily and intermittent PrEP regimens; (3) to evaluate changes in HIV-associated risk behavior; and (4) to evaluate HIV-specific immune responses in volunteers randomized to FTC/TDF and placebo.

### Outcomes

The main outcome measures were (1) clinical adverse events including mild, moderate and greater severity renal toxicities and serious adverse events, (2) willingness to use the study regimen, if shown to be effective, (3) adherence rates for daily and intermittent dosing, (4) change in HIV-associated risk behavior during trial participation, (5) the proportion of volunteers with HIV-specific immune responses as measured by Interferon-γ ELISpot.

### Sample size

Seventy-two volunteers were randomized to daily and intermittent FTC/TDF regimens (24 active and 12 placebo recipients per group). Because this was an exploratory study to evaluate safety, adherence and acceptability of intermittent PrEP, it was understood at the outset that this study had limited power to rule-out smaller differences in safety and adherence. For example, with 24 volunteers assigned to either daily or intermittent FTC/TDF, if no drug-related serious toxicities were observed, the upper limit of the two-sided exact 95% confidence interval for the corresponding true incidence was 14.3%. Therefore, observing no drug-related severe toxicities provided 97.5% confidence that the true incidence was no more than 14.3%. With 36 volunteers assigned to active and placebo for each regimen, the study had 51% power to detect an adherence rate of 90% for each regimen. Assuming condoms are used always or frequently (more than half the time) by 60% of volunteers at baseline, the study had >80% power to detect a 50% decrease in condom usage by treatment group (n = 24).

### Randomization

A random allocation sequence was generated by an external data coordinating center. Study product was randomly assigned in mixed blocks of 3 and 6, and the dosing schedule randomly assigned within study product using a block size of 2, stratified according to site. Investigators at the study sites enrolled volunteers via an electronic enrollment system (administered by the data coordinating center), where allocation codes were assigned consecutively to eligible volunteers at the time of first dispensation of study drug.

### Blinding

Allocation to FTC/TDF or identical placebo pill was blinded to study volunteers, all research staff and the study sponsor. Allocation to daily or intermittent dosing was not blinded.

### Adherence calculations

The unadjusted monthly MEMS adherence for the daily group was calculated as the number of MEMS events in 28 days divided by 28 days. Both the monthly self-report of curiosity openings (openings without removing pills) and monthly self-report of pocket doses (removal of multiple pills at one opening) were used to estimate adjusted MEMS adherence. The number of curiosity openings was subtracted from the number of MEMS openings, while the number of pocket doses was added to the number of MEMS openings. Estimates were calculated over a 28 day interval. Unadjusted monthly MEMS adherence for the intermittent group was calculated as the sum of days when volunteers were adherent to fixed dosing (Mondays and Fridays with a MEMS event, and non-Mondays and non-Fridays on which neither sexual activity nor a MEMS event occurred) plus post-coital dosing (other days on which sexual activity was reported by SMS and a MEMS event occurred), divided by 28. In a post-hoc analysis, intermittent dosing was adjusted for extra pills taken out of the MEMS bottle, with all pills assigned to post-coital. Adherence was also assessed by monthly self-report using timeline followback tools. Because SMS response rates varied (see below), we also calculated adherence to post-coital doses as the number of days with both MEMS events and timeline followback reported sexual events divided by the number of days with timeline followback reported sexual events. Adherence for post-coital doses within 2 hours of sex was calculated as the number of days post-coital dosing within 2 hours of sex by timeline followback report divided by the number of days with sexual events per SMS. It was also calculated as the number of days post-coital dosing within 2 hours of sex occurred by timeline followback report divided by the number of days sexual events occurred by timeline followback report. Acceptability was assessed by Likert scale during the timeline followback face-to-face assessment after 16 weeks in the study. Volunteers with missing responses were excluded from the denominator.

### Statistical methods

Comparisons of categorical and continuous factors were conducted using the Fisher's exact test and Wilcoxon rank-sum test, respectively. A two-sided p value of less than 0.05 was considered to indicate statistical significance. Statistical analyses were performed with the use of SAS software, version 9.2 (SAS Institute, Cary, NC, USA).

### Laboratory methods

Screening and clinical safety laboratories were performed on-site following Good Clinical Laboratory Practices (GCLP) in labs accredited by Qualogy UK [25]. Interferon-γ ELISpots were performed on frozen peripheral blood mononuclear cells (PBMCs) at KAVI and Kilifi laboratories on baseline and follow-up specimens as described previously [26]. A positive ELISpot was defined as (1) an average background(mock)-subtracted count per peptide greater than 38 spot forming units (SFU) per 10^6^ PBMCs with the coefficient of variation no greater than 70%, (2) mean count greater than 4× mean background (mock), and (3) mean background (mock) below 50 SFU/10^6^ PBMCs.

## Results

### Participant flow

**Figure 1:**
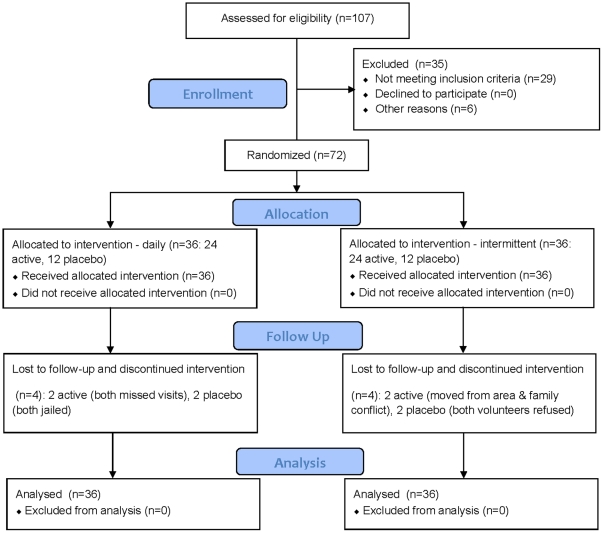
Flow of participants.

A total of 107 volunteers were screened for eligibility and 72 volunteers, 67 men and 5 women, were randomized into the study between October 19, 2009 and December 10, 2009. Enrollment of women was limited in order to maintain a primarily MSM study focus while avoiding community identification of the study as MSM-exclusive. Laboratory abnormalities were the most common reason for being excluded from participating, 22/35 (63%). The most common laboratory abnormalities at baseline were creatinine clearance calculated by the Cockcroft-Gault formula <80 mL/min, 12/22 (55%) and proteinuria on urine dipstick, 4/22 (18%). Follow-up continued until May 27, 2010, and 64 volunteers (89%) completed the study (Figure 1). The trial stopped when the end of the follow-up period was reached for all volunteers who completed the study.

### Baseline data

Baseline demographic and risk for HIV infection characteristics were similar between groups (Table 1). Forty-seven percent of volunteers reported drinking alcohol before sex, 67% reported transactional sex and 64% reported having receptive anal sex in the past 28 days. Volunteers in Kilifi were more likely to report using illicit drugs than those in Nairobi (61% vs. 22%, p = 0.022). All of the women were enrolled from the Kilifi site, since the Nairobi recruitment cohort did not include women at the time of study initiation.

**Table 1:** Baseline demographics and HIV risk behavior for the past 28 days by treatment assignment-schedule and study site.

	Treatment						
	Active		Placebo		Site		Total (N = 72)
	Daily (N = 24)	Intermittent (N = 24)	Daily (N = 12)	Intermittent (N = 12)	Kilifi (N = 36)	Nairobi (N = 36)	
**Male gender – no. (%)**	21 (88)	24 (100)	11 (92)	11 (92)	31 (86)	36 (100)	67 (93)
**Age – yr (mean (range))**	26 (20–36)	26 (19–35)	27 (20–38)	28 (18–46)	26 (18–36)	27 (20–46)	26 (18–46)
**Drank alcohol before sex – no. (%)**	9 (38)	14 (58)	5 (42)	6 (50)	15 (42)	19 (53)	34 (47)
**Used any street drugs – no. (%)**	8 (33)	10 (42)	7 (58)	5 (42)	22 (61)	8 (22)*	30 (42)
**Genital sore or discharge – no. (%)**	1 (4)	0 (0)	0 (0)	1 (8)	1 (3)	1 (3)	2 (3)
**Any condom use with new male partners – no. (%)**	11 (85)	11 (100)	5 (83)	7 (100)	19 (90)	15 (94)	34 (92)
**Any condom use with new female partners – no. (%)**	5 (100)	6 (100)	4 (100)	1 (100)	7 (100)	9 (100)	16 (100)
**Gave or received money/gifts for sex – no. (%)**	17 (74)	15 (63)	8 (73)	7 (58)	26 (74)	21 (60)	47 (67)
**Engaged in group sex – no. (%)**	1 (4)	0 (0)	0 (0)	0 (0)	1 (3)	0 (0)	1 (1)
**Had receptive anal sex – no. (%)**	13 (59)	17 (71)	5 (45)	9 (75)	19 (56)	25 (71)	44 (64)
**Had insertive anal sex – no. (%)**	13 (65)	14 (61)	8 (80)	6 (55)	18 (62)	23 (66)	41 (64)
**Number of sex partners past month – median [IQR]**	3 [2]–[4]	3 [2]–[4]	3 [1.5–4.5]	3 [1.5–4]	3 [1.5–4]	3 [2]–[4]	3 [2]–[4]

* P = 0.022.

### Participants analyzed

Analysis of safety, adherence, acceptability, and change in risk behavior include all randomized volunteers who were HIV-1 negative at the time of randomization and for whom study treatment was dispensed.

### Outcomes and estimation

#### Safety

Ninety-seven percent of non-serious adverse events (AEs) were mild or moderate, with 91% judged unlikely related or not related to study drug. The proportion of volunteers with moderate or above AEs did not differ by regimen (daily: 53%, intermittent: 56%, p = 1.00), or treatment groups (active: 60%, placebo: 42%, p = 0.14). The proportion with mild AEs was also not different by regimen or treatment group (Table 2). The percentage of volunteers with gastrointestinal complaints was not significantly higher in active (42%) than placebo recipients (21%) (p = 0.12). Among those on the daily regimen, a total of 82 adverse events (AEs) occurred; 91% were judged to be unlikely related or unrelated to the study product; 96% were mild or moderate. A similar pattern was observed among those on the intermittent regimen with a total of 76 AEs of which 89% were unlikely related or unrelated to the study product; 99% were mild or moderate. Two volunteers had asymptomatic elevated plasma bilirubin levels that were graded as very severe and unlikely related to study drug. Two volunteers had a transient episode of neutropenia each that was graded as severe and unlikely related to study drug. These AEs resolved spontaneously. Mild creatinine elevations (1.1–1.3 times the upper limit of normal) were observed in three volunteers on active regimen, all of which resolved spontaneously while continuing study drug. Two cases of abnormal creatinine clearance were detected, one in active and one in placebo recipients, which resolved spontaneously. No other clinically significant renal dysfunction was found. No drug-related SAEs were reported. One HIV infection occurred in a volunteer in the placebo group at week 16.

#### Adherence

There was no difference in adherence rates between treatment and placebo groups, thus these groups were combined for the adherence analyses (Table 3). Median unadjusted MEMS adherence rates were 83% [IQR: 63–92] for daily dosing and 55% [IQR: 28–78] for fixed intermittent dosing (p = 0.003), while adherence to any post-coital doses was 26% [IQR: 14–50]. Adherence rates did not change when adjusted for curiosity openings (i.e. when no pills were taken out). When adjusted for extra openings and extra pills taken out, median MEMS adherence for daily dosing was 92% [IQR: 82–99]. In a post-hoc analysis, intermittent dosing was adjusted for extra pills taken out, as defined above, with all pills assigned to post-coital dosing. Median MEMS adherence for post-coital dosing increased to 88% [IQR: 50–152]. There was no difference by study site in daily, fixed intermittent or post-coital dosing adherence rates. The median number of days per week of PrEP use according to MEMS data was 5.8 [IQR 4.4–6.4] in the daily group and 2.1 [IQR 1.3–2.7] in the intermittent group.

**Table 2:** Number (percentage) of volunteers with AEs categorized by maximum severity experienced, and treatment assignment and schedule.

Assignment Schedule		Maximum AE Severity				
		None	Mild	Mod	Severe	Very Severe
**Active**	**Daily**	4 (17)	6 (25)	11 (46)	2 (8)	1 (4)
	**Intermittent**	5 (21)	4 (17)	14 (58)	0 (0)	1 (4)
	**Overall**	**9 (19)**	**10 (21)**	**25 (52)**	**2 (4)**	**2 (4)**
**Placebo**	**Daily**	2 (17)	5 (42)	5 (42)	0 (0)	0 (0)
	**Intermittent**	4 (33)	3 (25)	5 (42)	0 (0)	0 (0)
	**Overall**	**6 (25)**	**8 (33)**	**10 (42)**	**0 (0)**	**0 (0)**

**Table 3:** PrEP adherence rates for daily and intermittent groups.

		Active	Placebo	Overall
**DAILY ADHERENCE RATE**	Overall unadjusted	82% [62–89]	84% [63–96]	83% [63–92]
**Median [IQR]**				
	Adjusted*	92% [79–101]	93% [84–96]	92% [82–99]
**INTERMITTENT ADHERENCE RATE**	Overall unadjusted	72% [62–80]	68% [63–76]	68% [63–78]
**Median [IQR]**				
	Fixed doses	56% [31–88]	34% [19–72]	55% [28–78]
	Post-coital doses	32% [13–50]	19% [14–45]	26% [14–50]
	Post-coital doses – (MEMS events and self report sexual events)	27% [13–60]	16% [8]–[37]	23% [13–50]
	Post-coital doses within****2 hrs** (self report and sexual****events per SMS)	115% [57–175]	100% [61–174]	105% [57–175]
	Post-coital doses within****2 hr (self report doses and****sexual events)	100% [100–100]	100% [67–100]	100% [96–100]

* Adjusted accounts for extra openings and extra pills taken out.

** Days on which sexual event reported per SMS.

#### SMS response rates and sexual activity

SMS responses were the primary measure of sexual activity used to calculate overall intermittent dosing adherence and specifically post-coital adherence. The median daily SMS response rate was 23% (range, 0–80), increasing to 33% (range, 0–100) when days with major SMS server outages (>2 hour outage) are excluded. Major server outages occurred on 65/316 (21%) days. Problems with SMS gateway providers required changing mobile phone networks and SIM cards, and SMS responses decreased by over 60% among volunteers who were requested to change SIM cards. Loss of mobile phones was rare.

Because SMS response rates were low, we also calculated post-coital adherence using timeline followback self-report data. The median number of days per week when sex occurred by SMS reporting was 1.4 [IQR: 0.4–2.5] in the daily group and 0.7 [IQR: 0.4–1.9] in the intermittent group. By timeline followback, it was 1.8 [IQR: 0.9–3.9] in the daily group and 1.0 [IQR: 0.7–1.6] in the intermittent group. Using MEMS and timeline followback sexual activity data, the median MEMS adherence rate for post-coital dosing was 23% [IQR: 13–50]. Using timeline followback adherence data and SMS sexual activity data, adherence for post-coital doses within 2 hours of sex was 105% [IQR: 57–175]. Using only timeline followback data for adherence and sexual activity data, it was 100% [IQR: 96–100].

The most common reasons cited for missing a pill dose were being away from home (12%), not having pills (11%), forgetting to take pill (8%), a change in daily routine (7%), and using alcohol or drugs (3%). There were no differences between daily and intermittent groups in reasons for missing a pill dose.

#### Acceptability

Eighty-three percent (60/72) of volunteers would be willing to use the pill regimen most or all of the time if it was shown to be safe and effective and was inexpensive or free. There was no difference in acceptability between daily and intermittent groups (80% vs. 86%), or between active and placebo groups (86% vs. 80%). Acceptability of using the MEMScap was high with 88% (62/72) reporting it was somewhat or very easy to use. Eleven volunteers required replacement of MEMS cap due to loss or damage.

#### HIV behavior change

The median number of sexual partners in the past month increased from 3 [IQR 2–4] at baseline to 4 [IQR 2–8] at month 4 during the trial, primarily due to an increase of 3 [IQR 1.5–4] to 5 [IQR 2–12] at the Kilifi site. Because there may have been underreporting of sex partners at baseline, we also compared month 2 and month 4; the median number of sexual partners was 4 at month 2 and at month 4. No other HIV risk behaviors reported at baseline changed during the trial.

#### HIV-specific immune responses

Minimal HIV-specific immune responses were detected by IFN-γ ELISpot at baseline; two of 53 (3.8%) volunteers with specimens available for ELISpot testing had a positive IFN-γ ELISpot response at baseline, one to a Nef peptide and one to all 6 peptide pools. Only four of 62 (6.5%) volunteers with post-baseline ELISpot data had a response to one or more peptides at a single time point. One volunteer assigned to the intermittent active treatment group had positive IFN-γ ELISpot responses to a Pol/Int peptide during the study at week 12 and 16. Two volunteers in the intermittent active treatment group responded to an Env peptide pool at week 16. One volunteer in the intermittent placebo treatment group responded to the RT peptide pool at week 16. The IFN-γ ELISPOT responses were generally low magnitude.

## Discussion

### Interpretation and Overall Outcome

To our knowledge, this is the first report of safety and adherence to an intermittent PrEP regimen. It describes the use of both daily and intermittent PrEP regimens in a primarily MSM population in sub-Saharan Africa, which is a key risk group that may be targeted for PrEP. In addition, this is the first published report of the use of electronic medication monitoring for PrEP adherence. Safety with FTC/TDF for PrEP was similar for both daily and intermittent users. Similar to the larger iPrEX study using FTC/TDF, the Partners PrEP trial using FTC/TDF and TDF, and the Botswana TDF2 trial using FTC/TDF, we found no significant drug safety patterns [3, Baeten 2011, Thigpen 2011]. Our study adds to the safety data on FTC/TDF PrEP for African men with 48 volunteers in the active treatment group, compared to 45 African men on active treatment in the iPrEX study. However our safety data reflects only short-term use over a four month period, with varying levels of adherence among volunteers. Like the iPrEX study, a higher proportion of volunteers in the active treatment group had gastrointestinal complaints, but this was not statistically significant. No evidence of renal dysfunction was found. Acceptability of PrEP was high, and interestingly, did not differ between daily and intermittent users.

Median adherence as measured by MEMS was significantly higher for daily use, 83%, compared to either component of the intermittent regimen, either the fixed Monday and Friday doses, 55%, or the post-coital doses, 26%. After adjusting the daily adherence measurement for extra pills taken out, median adherence to daily dosing may have been as high as 92%. The unadjusted median adherence for daily use fell in between self-reported or clinic-based pill count measures of adherence in the iPrEx study, 95% and 89–95% respectively, and clinic-based pill count in the West African study, 74%. However, both self-report and clinic-based pill count have been shown to be inaccurate measures of adherence in many settings, typically overestimating adherence compared to electronic monitoring, unannounced home-based pill counts, and random drug levels [27]–[29]. In contrast, preliminary data from a PrEP study in HIV discordant heterosexual couples in East Africa found near perfect adherence rates by multiple measures, 99.1–101.9% by MEMS, unannounced home-based pill counts or clinic-based pill counts. (Haberer J, Baeten J, Celum C, Tumwesigye E, Katabira E et al. (2011) Near Perfect Early Adherence to Antiretroviral PrEP against HIV Infection among HIV Serodiscordant Couples as Determined by Multiple Measures: Preliminary Data from the Partners PrEP Study [Abstract 488]. In: Proceedings of the 18th Conference on Retroviruses and Opportunistic Infections; 27 February–2 March 2011; Boston, Massachusetts, United States. CROI 2011. Available: http://www.retroconference.org/2011/Abstracts/41269.htm. Accessed 14 March 2012.) This high level of adherence may be due to providing PrEP in the context of stable, socially supported relationships as well as the uninfected partner's hope of maintaining good health while at the same time preserving their serodiscordant relationship [30]. These factors are less likely to play a role in the primarily MSM population in our study.

The lower rates of adherence to fixed and post-coital dosing may be due to either true lower adherence to these dosing schedules, or inaccurate measurement for these regimens using MEMS and SMS reporting. Volunteers were allowed to take extra doses out of the MEMS bottle, for use during travel and/or for post-coital dosing away from home, and even encouraged to do so by distribution of key-chain pill holders. Therefore, it was difficult to know whether self-reported extra pills taken out should be assigned to fixed doses or post-coital doses. However, if all self-reported extra pills taken out were assigned to post-coital dosing, median adherence for post-coital dosing increased from 26% to 88%, suggesting that lower rates of adherence to post-coital dosing may be due in part to inaccurate measurement of this type of dosing with MEMS reporting. However, assessment of adherence to an intermittent regimen including post-coital doses requires self-report measurement of sexual activity, which is known to be inaccurate [31], [32] We used daily SMS reporting of sexual activity as the primary measure with that hope that frequent measurements would decrease recall bias. However SMS response rates varied due to technical and behavioral reasons and overall were low. By substituting timeline followback report of sexual activity for SMS data, median post-coital adherence rate did not improve, 26%, since self-reported sexual activity was low by both measures. However, using exclusively timeline followback self-report data for both adherence and sexual activity, median post-coital dosing adherence was 100%, as was median adherence to post-coital dosing within 2 hours of sex, 100%. These perfect adherence rates suggest that self-report overestimated both pill-taking and sexual activity in this study. Our data highlights the difficulty of measuring adherence to a coitally-dependent regimen. Novel measures providing more accurate data on sexual behavior are needed to improve our assessment and understanding of this type of intermittent dosing. In addition, qualitative data could provide deeper insight into barriers to coitally-dependent PrEP.

Volunteers in daily and intermittent groups reported a median 1.4 and 0.7 sex acts per week by SMS, respectively, and a median 4 sex partners per month by timeline followback self-report, which appears lower than what has been measured previously in at least one of these cohorts. In a comparison study of interview methods at cohort enrolment of MSM and FSW in Kilifi, audio computer-assisted self-interview (ACASI) captured double the median number of regular and casual partners per week compared to face-to-face interview (2 vs. 1) [33]. In a prospective diary study of MSM in the Kilifi cohort, we found high rates of sexual activity: 83 volunteers reported having a median 2.5 sex partners per week and 3.6 sex acts per week (Smith AD, Ferguson A, Kowour D, Van Der Elst E, Agwanda C, et al. (2009) Role Versatility and Female Partnerships among Men Who Sell Sex to Men: Mombasa, Kenya [Abstract 1029]. In: Proceedings of the 16th Conference on Retroviruses and Opportunistic Infections; 8–11 February 2009; Montreal, Canada. CROI 2009. Available: http://www.retroconference.org/2009/Abstracts/36217.htm. Accessed 14 March 2012.) The apparent under-reporting of sexual activity in this study may be due to various causes, including a preference to remember behaviors that occurred on days that pills were taken, unfamiliarity with daily reporting of sexual behavior through SMS, and technical difficulties with SMS.

Lower adherence to intermittent fixed and post-coital dosing may have been due to several barriers. Daily pill-taking behavior is more routine and may be easier to remember than fixed intermittent, twice weekly doses. Few published studies address adherence to less than daily medication regimens, even in the tuberculosis field, where intermittent dosing regimens are common. Changes in routine due to illness, travel or family obligations have been correlated with adherence difficulties with daily regimens; these factors are likely to impact intermittent regimens as well [34]. Perceived HIV risk may influence adherence. If perceived HIV risk is low on the assigned days, adherence may be lower, whereas daily pill adherence may involve less frequent consideration of perceived HIV risk. Perceived HIV risk may influence post-coital adherence depending on the serostatus of the recent sexual partner if known, whether the encounter was anal insertive or receptive, protected or unprotected by condoms, and whether the partner was known or anonymous. Perceived HIV risk has been associated with partner-specific differences in condom use, which, although less covert, is a reasonable comparison for post-coital PrEP [35]–[37]. Close to half of volunteers reported using alcohol before sex. Such substance use around the time of sex could reduce adherence to post-coital dosing. Alcohol use was reported as a barrier to acyclovir adherence in another herpes suppression trial in Africa [38]. In addition, stigma of disclosing HIV at-risk status by taking medication in the presence of a partner may reduce post-coital adherence. Due to the relatively high frequency of transactional sex in this population, volunteers may travel for sex and may not have pills with them at the time for post-coital dosing. It is not clear whether pre-coital dosing would be more successful than post-coital dosing. Pre-coital dosing may still be hampered by travel and location issues as well as drug impairment. Allowing a longer window, e.g. morning dosing on the day of sex, might avoid these issues, but predicting the likelihood of sexual activity later in the day may be unreliable.

Our findings suggest that adherence to intermittent dosing regimens, fixed doses and in particular coitally-dependent doses, may be more difficult than adherence to daily dosing. However, intermittent dosing may still be appropriate for PrEP if intracellular drug levels, which correlate with prevention of HIV acquisition, can be attained with less than daily dosing and if barriers to adherence can be addressed. Forthcoming data from the present study will analyze plasma and intracellular drug levels in both the daily and intermittent groups to determine median drug levels attained at different strata of adherence. Data from ongoing directly observed dosing studies [39] and PrEP efficacy trials [40], [41] may provide sufficient information to determine what intracellular drug levels can be achieved with intermittent dosing and to define the minimum protective drug concentration. Therefore data on drug levels may be key to establishing what level and pattern of adherence is necessary for PrEP effectiveness. If twice weekly dosing of FTC/TDF can produce intracellular drug levels that correlate with efficacy, then this regimen may be appropriate for PrEP if the adherence rate can be improved from the median 55% found for twice weekly fixed dosing in this population.

Despite encouraging data from animal studies, we found no evidence of HIV-specific immune responses as measured by a validated IFN-γ ELISpot in volunteers on PrEP. It is possible that a longer duration of PrEP and/or more intensive mucosal exposures are necessary to develop these responses, or that too few volunteers were exposed to HIV in this trial. Alternatively, if PrEP-related protective responses do exist, they may be located in the mucosa and may not be evaluable with T-cell assays in PBMCs such as IFN-γ ELISpots.

### Generalizability

Our findings on PrEP safety, acceptability and adherence in African MSM may be applicable to MSM in other settings, particularly in the developing world and in groups with high rates of transactional sex and alcohol use. However our study has several limitations. Only a small number of women participated in the study, limiting generalization of results to this population. Sixty-three percent of ineligible volunteers were excluded for lab abnormalities, with decreased creatinine clearance and proteinuria accounting for 85% of these exclusions. We used a conservative creatinine clearance cut-off of <80 mL/min, which may account in part for the relatively high rate of lab-based exclusions in our study. However the percentage of all screened volunteers who were excluded due to decreased creatinine clearance was only 11%, which should not diminish the generalizability of the results. The duration of follow-up was short and may not reflect safety and adherence outcomes for longer-term use of PrEP. Some volunteers likely had low levels of drug exposure, particularly in the intermittent group, where the median number of days per week of PrEP use was 2.1 [IQR 1.3–2.7]. However, safety outcomes were similar for the intermittent and daily groups, and reflected findings of larger PrEP studies with longer durations of exposure. Volunteers were recruited from existing HIV prevention cohorts in which volunteers received ongoing HIV risk reduction counseling and preventive services, so their behavior may not reflect the larger population at risk for HIV. Although the majority of volunteers had mobile phones and used SMS messaging prior to enrollment, none had experience responding to daily SMS messages for research purposes, particularly questions about sexual activity. It is possible that SMS response rates would be higher once technical outages had been resolved and volunteers had additional familiarity with this technology in a research setting.

Our data suggest that although adherence is lower than with daily regimens, intermittent PrEP dosing with a fixed regimen in this at-risk population is feasible. Additional drug level data, qualitative data on adherence barriers, and better methods to measure sexual activity are necessary to determine whether adherence to post-coital PrEP could be comparable to more standard regimens. Larger studies of intermittent PrEP with longer follow-up in additional at-risk populations can examine whether fixed intermittent PrEP regimens can achieve drug levels comparable to the minimum effective concentration which may be determined by ongoing efficacy trials. Alternate approaches to peri-coital dosing might be more successful. Additional work on using SMS to assess sexual activity may be warranted.

## Supporting Information

Protocol S1 **Trial Protocol.**
(PDF)

Checklist S1 **CONSORT Checklist.**
(DOC)
