# Supplementary material for: Safety and Adherence to Intermittent Pre-Exposure Prophylaxis (PrEP) for HIV-1 in African Men Who Have Sex with Men and Female Sex Workers
Source: PLoS One. 2012 Apr 12;7(4):e33103. doi: 10.1371/journal.pone.0033103 (PMC3325227; doi:10.1371/journal.pone.0033103)
Supplement: Protocol S1 — Trial Protocol. (PDF) [file pone.0033103.s001.pdf]

TITLE PAGE

**Protocol Title:**	A pilot study of Pre-Exposure Prophylaxis (PrEP) to evaluate safety, acceptability, and adherence in at-risk populations in Kenya, Africa
**Protocol Number:**	IAVI E001
**Phase:**	Phase 1/2
**KENYA**	
**Study Chair:**	Prof. Omu Anzala MB.ChB, Phd<br>University of Nairobi
**Principal Investigator, KEMRI-Kilifi:**	E.J. Sanders MD, MPH, PhD<br>Kenya Medical Research Institute-Kilifi
**Principal Investigator, KAVI- Kangemi:**	Dr Gaudensia Mutua MPH, MB.ChB<br>University of Nairobi
**Sponsor:**	International AIDS Vaccine Initiative (IAVI)<br>110 William Street, 27th Floor<br>New York, New York 10038-3901<br>USA
**Investigational Product provider:**	Gilead Sciences, Inc
**Sponsor Status:**	Non-Profit Organization
**Date of Protocol Version:**	09-Mar-2009<br>2.0

The **CONFIDENTIAL INFORMATION** in this document is provided to you as an Investigator, potential Investigator, or Consultant, for review by you, your staff, and applicable institutional review boards (IRBs) and/or independent ethics committees (IECs). It is understood that the information will not be disclosed to others, except to the extent necessary to obtain ethical and regulatory approval from the respective committee's agencies and informed consent from those persons to whom the investigational product may be administered.

SYNOPSIS

**TITLE:**	A pilot study of Pre-Exposure Prophylaxis (PrEP) to evaluate safety, acceptability, and adherence in at-risk populations in Kenya, Africa
**PROTOCOL NUMBER:**	IAVI E001
**PHASE:**	Phase 1/2
**SPONSOR:**	International AIDS Vaccine Initiative (IAVI)<br>110 William Street, 27th Floor<br>New York, New York 10038-3901,<br>USA
**Sponsor Status:**	**Non-Profit Organization**
**OBJECTIVES:**	***Primary:***<br>• To evaluate the safety of daily and intermittent dosing (twice a week and within 2 hours after sex with no more than one dose per day) of emtricitabine/tenofovir disoproxil fumarate (FTC/TDF)<br>• To compare the acceptability of and adherence to daily and intermittent regimens<br>• To evaluate mean intracellular drug levels in the daily and intermittent regimens<br>• To evaluate the relationship between adherence and intracellular drug levels<br>• To evaluate changes in HIV associated risk behaviour<br>***Other:***<br>• To prepare study teams for larger PrEP trials<br>• To evaluate methods for measuring adherence and sexual activity<br>• To evaluate potential for medication sharing<br>• To evaluate HIV-specific immune responses in volunteers randomized to FTC/TDF and to placebo
**ENDPOINTS:**	***Primary**:*<br>*Safety and tolerability:*<br>• The proportion of volunteers with moderate and greater severity clinical adverse events<br>• The proportion of volunteers with mild, moderate and greater severity of renal toxicities, and other moderate and severe laboratory abnormalities.<br>• The proportion of volunteers with serious adverse events.

<table>
<tr><td></td><td>Acceptability:
The proportion of volunteers who report willingness to use the study regimen
Intracellular drug concentrations:
The mean intracellular drug concentration for each group assigned to FTC/TDF
Adherence:
The proportion of volunteers who took, by MEMS (Medication event monitoring system) data, at least 80% of expected doses of the investigational product
The proportion of volunteers assigned to FTC/TDF who have detectable drug plasma levels within 48 hours of reported use.
The relationship between intracellular drug levels and adherence in volunteers assigned to FTC/TDF
Behavioral:
Reported number of steady and casual sex partners
Frequency of unprotected vaginal and/or anal intercourse
Substance use prior to or during sex
Other:
The proportion of volunteers who report somewhat high or high levels of burden in using electronic medication monitoring to measure adherence, and using cell phone communication to measure sexual activity
The proportion of study days with missing sexual activity data
The proportion of volunteers who report sharing medications
The proportion of volunteers assigned to placebo who have detectable intracellular drug levels
The proportion of volunteers with HIV-specific immune responses as measured by analysis of cellular or humoral immune response, or changes in gene regulation as measured by microarray or proteomic techniques</td></tr>
<tr><td>STUDY DESIGN</td><td>Randomized, placebo-controlled blinded study with volunteers assigned to FTC/TDF daily, FTC/TDF intermittent, placebo daily, or placebo intermittent in a 2:2:1:1 ratio

<table><tr><td>Investigational Product</td><td>Dosing Regimen</td><td>Route of Administration</td></tr><tr><td>FTC/TDF</td><td>Daily</td><td>PO</td></tr><tr><td>FTC/TDF</td><td>Intermittent</td><td>PO</td></tr><tr><td>Placebo</td><td>Daily</td><td>PO</td></tr><tr><td>Placebo</td><td>Intermittent</td><td>PO</td></tr></table></td></tr>
</table>

	Daily dosing: 1 tablet orally per day<br><br>Intermittent dosing: 1 tablet orally Monday and Friday and again within 2 hours after sexual intercourse (except on Monday or Friday), not to exceed 1 dose per day.<br><br>PO = Oral
**METHODS:**	See Schedule of Procedures; Appendix A
**STUDY POPULATION:**	Healthy, HIV-uninfected male or female adults 18-49 years of age, at risk for HIV, who are willing to undergo HIV testing, counselling and receive HIV test results, if female to use an effective method of contraception, and who, in the opinion of the principal investigator or designee, understand the study and who provide written informed consent.<br><br>Principal exclusion criteria include confirmed HIV-1 or HIV-2 infection; pregnancy and lactation; chronic disease; confirmed chronic hepatitis B infection; clinically significant abnormal laboratory values.
**NUMBER OF VOLUNTEERS:**	Approximately 72 volunteers (24 FTC/TDF daily; 24 FTC/TDF intermittent; 12 placebo daily; 12 placebo intermittent) will be included An over-enrolment of up to 10% (approximately 7 additional volunteers) will be permitted in the study.
**DESCRIPTION OF INVESTIGATIONAL PRODUCT:**	This study evaluates the use of FTC/TDF (tenofovir disoproxil fumarate 300mg/ emtricitabine 200mg coformulation) or placebo;
**BLINDING:**	Study staff and volunteers will be blinded with respect to the allocation of placebo or FTC/TDF, but dosing regimen, daily or intermittent, will not be blinded.
**DURATION OF STUDY PARTICIPATION:**	Volunteers will be screened up to 42 days before randomization and will be followed for 4 months after the randomization.<br>It is anticipated that it will take approximately 3 months to enrol the study.
**EVALUATION FOR INTERCURRENT HIV INFECTION:**	Volunteers will be clinically evaluated and tested for HIV-1 and HIV-2 antibodies at time points specified in the Schedule of Procedures. Test results will be interpreted according to a pre-determined diagnostic algorithm.
**STATISTICAL CONSIDERATIONS:**	Collected data will be identified only by a volunteer identification number. At the end of the study, a full analysis will be prepared according to a pre-specified statistical analysis plan. All analyses will be based on the modified intention-to-treat cohort and will analyze volunteers according to the group to which they were randomized.<br><br>Safety and tolerability will be addressed by examining the overall rates of adverse events and serious adverse events that might be associated with FTC/TDF daily and FTC/TDF intermittent regimens. All clinical and routine laboratory data will be included in the safety analysis. With 24 volunteers assigned to daily or intermittent

	FTC/TDF, observing no drug-related serious toxicity would result in an exact, two-sided, 95% confidence interval of (0.0000, 0.1425). If one drug-related SAE is observed, the exact, two-sided, 95% confidence interval would be (0.0011, 0.2112).<br><br>The proportion of volunteers who took at least 80% of the study medication recorded using Medication Event Monitoring System (MEMS) data will be estimated. For the intermittent group, since the dosing frequency is dependent on sexual activity, data collection using cell phone communication will be used to determine daily sexual activity in order to determine the number of expected doses. Adherence will be expressed as overall percent adherence over the study interval, longitudinal adherence by month, and for the intermittent regimen, adherence to twice weekly scheduled doses and adherence to doses during days of sexual activity. With 36 volunteers (combining active and placebo for each regimen), this study will have 51% power to detect a true adherence rate of 90% for each regimen. Power increases to 90% if the true adherence rate is 95%.<br><br>The proportion of volunteers assigned to FTC/TDF with detectable plasma FTC/TDF levels within 48 hours of a MEMS event will be estimated. Levels of FTC/TDF in hair samples will be evaluated for exploratory purposes only.<br><br>Assuming intracellular drug level has a log-normal distribution with a between subject standard deviation of 1.0 on the natural logarithm scale, this study should have about 79% power to rule-out a two-fold or greater relative decrease in the geometric mean concentration due to intermittent versus daily FTC/TDF. If the between subject standard deviation is 0.5, then there will be about 80% power to rule-out a 1.5-fold or greater decrease in the geometric mean concentration.<br><br>Intracellular drug levels will be correlated with adherence data for volunteers assigned to FTC/TDF for each regimen.<br><br>Descriptive statistics will be used to describe changes in HIV risk behaviour. Assuming condoms are used always or frequently by 60% of volunteers at baseline, this study has 85% power to detect a decrease in condom usage by treatment group (n=24) if volunteers reduce the rate to 30%. A decrease to a rate of 40% would be detected with 49% power. Power is based on a two-sided, 0.05 level test of a binomial proportion.<br><br>Descriptive statistics will be used to describe acceptability, medication sharing, and HIV-specific immune responses.<br>Assuming a 10% HIV-specific immune response rate in the combined placebo groups, this study will have about 83% power to detect an increased immune response rate due to intermittent PrEP if the true response rate is 50% in the intermittent FTC/TDF group.

TABLE OF CONTENTS

1.0 CONTACT INFORMATION....................10

2.0 SIGNATURE PAGE....................14

3.0 INTRODUCTION AND BACKGROUND INFORMATION....................15

4.0 STUDY OBJECTIVES....................33

4.1 PRIMARY OBJECTIVES....................33
4.2 OTHER OBJECTIVES....................34

5.0 STUDY ENDPOINTS AND STUDY DESIGN....................34

5.1 STUDY ENDPOINTS....................34
5.2 STUDY DESIGN....................35

6.0 STUDY VISITS....................38

6.1 SCREENING PERIOD....................38
6.2 RANDOMIZATION VISIT....................39
6.3 POST-RANDOMIZATION VISITS....................40
6.4 UNSCHEDULED VISITS/CONTACT....................40
6.5 FINAL VISIT/EARLY TERMINATION VISIT....................41

7.0 STUDY PROCEDURES....................42

7.1 INFORMED CONSENT PROCESS....................42
7.2 MEDICAL HISTORY AND PHYSICAL EXAMINATION....................43
7.3 HIV RISK ASSESSMENT, HIV TESTING AND HIV-TEST COUNSELLING....................43
7.4 HIV RISK REDUCTION COUNSELLING....................43
7.5 FAMILY PLANNING COUNSELLING....................44
7.7 BLOOD COLLECTION....................44
7.8 URINE COLLECTION....................45
7.9 VAGINAL AND RECTAL SWAB COLLECTION....................45
7.11 REIMBURSEMENT....................45
7.12 RANDOMIZATION AND BLINDING....................45
7.13 UNBLINDING PROCEDURE FOR INDIVIDUAL VOLUNTEERS....................46

8.0 INVESTIGATIONAL PRODUCT....................47

8.1 DESCRIPTION....................47
8.2 SHIPMENT AND STORAGE....................47
8.3 DISPENSING AND HANDLING....................48
8.4 DISTRIBUTION....................48
8.5 ACCOUNTABILITY AND DISPOSAL....................48

9.0 ASSESSMENTS....................49

10.0 ADVERSE EVENTS....................54

10.1 DEFINITION....................54
10.2 ASSESSMENT OF SEVERITY OF ADVERSE EVENTS....................54
10.3 RELATIONSHIP TO INVESTIGATIONAL PRODUCT....................55
10.4 SERIOUS ADVERSE EVENTS....................56
10.5 CLINICAL MANAGEMENT OF ADVERSE EVENTS....................57
10.6 PREGNANCY....................57
10.7 INTERCURRENT HIV INFECTION....................57

11.0 MANAGEMENT OF HIV ISSUES DURING AND FOLLOWING STUDY....................58

11.1 HIV TESTING....58
11.2 HIV INFECTION....58
11.2.1 Counselling....58
11.2.2 Referral for Support and/or Care....58

12.0 DISCONTINUATION OF ADMINISTRATION OF INVESTIGATIONAL PRODUCT AND/OR WITHDRAWAL FROM STUDY....59

12.1 DISCONTINUATION OF ADMINISTRATION OF INVESTIGATIONAL PRODUCT....59
12.2. FOLLOW UP AFTER DISCONTINUATION OF FURTHER ADMINISTRATION OF INVESTIGATIONAL PRODUCT 60
12.3 WITHDRAWAL FROM THE STUDY (EARLY TERMINATION)....60
12.4. Follow-up Withdrawal from the Study (Early Termination)....60

13.0 DATA HANDLING....61

13.1 DATA COLLECTION AND RECORD KEEPING AT THE STUDY RESEARCH CENTRE....61
13.2 DATA ENTRY AT THE STUDY RESEARCH CENTRE....61
13.3 DATA ANALYSIS....61

14.0 STATISTICAL CONSIDERATIONS....62

14.1 SAMPLE SIZE....62
14.2 STATISTICAL POWER AND ANALYSIS....62

15.0 QUALITY CONTROL AND QUALITY ASSURANCE....65

16.0 DATA AND BIOLOGICAL MATERIAL....66

17.0 ADMINISTRATIVE STRUCTURE....66

18.0 INDEMNITY....68

19.0 PUBLICATION....68

20.0 ETHICAL CONSIDERATIONS....68

REFERENCES....69

APPENDIX A: SCHEDULE OF PROCEDURES....79
APPENDIX B: ADVERSE EVENT GRADING TOXICITY TABLE

ABBREVIATIONS

Abbreviation	Term
AE	Adverse Event
AIDS	Acquired Immunodeficiency Syndrome
ALT	Alanine-Aminotransferase
ART	Anti-retroviral therapy
AST	Aspartate-Aminotransferase
ACASI	Audio-Computer-assisted self-interview
CFC	Cytokine Flow Cytometry
CMI	Cell Mediated Immunity
CMV	Cytomegalovirus
CRF	Case Report Form
CTL	Cytotoxic T Lymphocyte
DCC	Data Coordinating Centre
DNA	Deoxyribonucleic Acid
ELISA	Enzyme Linked Immunosorbent Assay
FTC/TDF	emtricitabine/tenofovir disoproxil fumarate
GCP	Good Clinical Practice
HAART	Highly Active Antiretroviral Treatment
HAV	Hepatitis A Virus
HBV	Hepatitis B virus
HIV	Human Immunodeficiency Virus
HLA	Human Leukocyte Antigen
HSV	Herpes Simplex Virus
IAVI	International AIDS Vaccine Initiative
ICH	International Conference on Harmonization
IEC	Independent Ethics Committee
IVR	Interactive Voice Response

Abbreviation	Term
Kg	Kilogram
MEMS®	Medication event monitoring system
mg	Milligram
MSM	Men who have sex with men
MSW	Male sex worker
NRTI	non-nucleoside reverse transcriptase inhibitors
PCR	Polymerase Chain Reaction
Pfu	Plaque Forming Units
PBMC	Peripheral Blood Mononuclear Cells
PrEP	Pre-Exposure Prophylaxis
iPrEP	Intermittent Pre-Exposure Prophylaxis
RPR	Rapid Plasma Reagin
SAE	Serious Adverse Event
SIV	Simian Immunodeficiency Virus
SHIV	Simian-Human Immunodeficiency Virus
SOP	Standard Operating Procedure
SOM	Study Operations Manual
SRSV	Small Round Structured Virus
SMS	Short Message Service
SRB	Safety Review Board
STI	Sexually Transmitted Infection
TDF	Tenofovir Disoproxil Fumarate
TPHA	Treponema Pallidum Hemagglutination

1.0 CONTACT INFORMATION

1.1 Sponsor Contact Information

Trial Sponsor

International AIDS Vaccine Initiative (IAVI)
Patricia Fast MD PhD, Chief Medical Officer
110 William Street, 27th Floor
New York, NY 10038-3901
USA

Telephone: + 1- 212-847-1068
Fax: + 1- 212-847-1113
Mobile: + 1- 646-206-6611

Medical Monitor

Frances Priddy MD MPH
Senior Director, Medical Affairs
International AIDS Vaccine Initiative (IAVI)
110 William Street, 27th Floor
New York, NY 10038-3901
USA

Telephone: +1-608-203-5141
Fax: +1-608-203-5501
Mobile: +1-646-287-8943
E-mail: fpriddy@iavi.org

Data Coordinating Center

The EMMES Corporation
401 N. Washington Street
Suite 700
Rockville, Maryland
20850,
USA

Telephone: + 1- 301-251-1161
Fax: + 1- 301-251-1355
E-mail: iavitrials@emmes.com

Trial Monitoring

International AIDS Vaccine Initiative (IAVI)
ABC Place, Waiyaki Way
Bulding 2, 3rd floor
P.O. Box 340-00202 KNH
Nairobi

Telephone: +254 (0) 20 44 53596/7
Fax: +254 (0) 20 44 53598

Core Laboratory
Jill Gilmour, Ph.D
Senior Director, Clinical Immunology Laboratory
Chelsea and Westminster Hospital
369 Fulham Road
London SW10 9NH

Telephone: +44 (0)20 8746 8257/8
Fax: +44 (0) 20 8746 5997
Mobile:
E-mail jgilmour@iavi.org

Tony Tarragona, Ph.D.
Lab Manager/Administrator
IAVI Core Lab
Imperial College London, Faculty of Medicine
St. Stephen's Centre, 5th Floor
Chelsea Campus
369 Fulham Road
London SW10 9NH

Telephone: + 44 (0) 20 8846 6300
Mobile: +44 (0) 7940510090
E-mail t.tarragona@imperial.ac.uk

Adherence analyses
David Bangsberg, MD, MPH
Jessica Haberer, MD
Massachusetts General Hospital
Harvard Initiative for Global Health
104 Mt. Auburn
Cambridge, MA 02138

Pharmacology analyses
Craig Hendrix MD
Professor of Medicine
Director (Interim),
Clinical Pharmacology Analytical Laboratory (CPAL)
Johns Hopkins University School of Medicine
Harvey 502
600 N. Wolfe St.
Baltimore, MD 21287
410-955-9707 voice
410-955-9708 fax

Hair sampling for adherence
Monica Gandhi **,** M.D., M.P.H.
Assistant Adjunct Professor II
University of California, San Francisco
Division of Infectious Diseases
Box 1352
405 Irving Street, 2nd floor
San Francisco, CA 94122-1352

Albert Liu, MD, MPH
Director, HIV Prevention Intervention Studies,
San Francisco Department of Public Health;
Assistant Clinical Professor of Medicine, UCSF

1.2 Collaborating Research Centre Contact Information

Study Chair

Professor Omu Anzala MB.ChB, Ph.D	Telephone:	+254 20 2725404
Kenya AIDS Vaccine Initiative	Fax:	+254 20 2714613
Kenyatta National Hospital	Mobile:	+254 727555279
Dept of Medical Microbiology	Email:	oanzala@kaviuon.org
University of Nairobi		
PO Box 19676		
Nairobi, Kenya		

Principal Investigator – Nairobi

Dr. Gaudensia Mutua MB.ChB, MPH		
Kenya AIDS Vaccine Initiative	Telephone:	+254 724256131
University of Nairobi	Fax:	+254 202712007
PO Box 19676 – 00202	Mobile:	+254 733826815
Nairobi, Kenya	E-mail:	gmutua@kaviuon.org

Principal Investigator - Kilifi

E.J. Sanders MD, MPH, PhD	Telephone:	+254-41-522133
Kenya Medical Research Institute,	Fax:	+254-41-522390
Ctr for Geographic Medicine Research – Coast	Mobile:	+254-72-359762
PO Box 230		
Kilifi, Kenya	Email:	ESanders@kilifi.kemri-wellcome.org

2.0 SIGNATURE PAGE

The signatures below constitute the approval of this protocol and the appendices and provide the necessary assurances that this study will be conducted in compliance with the protocol, GCP and the applicable regulatory requirement(s).

Sponsor:

Signed: ______________________________ Date: ______________

Dr. Patricia Fast
Chief Medical Officer, IAVI

Principal Investigator:

Signed: ______________________________ Date: ______________

Name (please print): ______________________________

Name of institution (please print): ______________________________

3.0 INTRODUCTION AND BACKGROUND INFORMATION

The ongoing worldwide epidemic of the human immunodeficiency virus type 1 (HIV-1) remains one of the major global health challenges. HIV-1 causes the acquired immunodeficiency syndrome (AIDS), which is responsible for tremendous human suffering and economic loss throughout the world. According to the Joint United Nations Programme on HIV/AIDS and the World Health Organization, as of the end of 2007, over 33 million people [30.6–36.1 million], were estimated to be living with HIV/AIDS, with 95% residing in the developing world [1]. It is estimated that in 2007 alone, 2.5 million [1.8–4.1 million] were newly infected with HIV. AIDS is the leading killer in Africa, with over 28 million Africans living with HIV/AIDS. Sub-Saharan Africa has been affected most; in 7 Sub-Saharan African countries, over 20% of adults (aged 15-49) are living with HIV/AIDS[1]. Unlike other regions, the majority of people living with HIV in sub-Saharan Africa (61%) are women, as the explosive spread of HIV in sub-Saharan Africa has occurred primarily through heterosexual contact [1,2].

Since 1996, potent new antiretroviral therapies (ART), including combination regimens with protease inhibitors, have created the possibility that HIV-1 infection might become a chronic, manageable disease among individuals with access to these medications. In the late 1990s, AIDS deaths in the US fell to 16,000 per year as a result of the new antiretroviral therapies [3]. However, for the developing world, these drugs are not yet widely accessible despite several years of increased funding and reduced drug prices, due to many logistical challenges associated with their use. Only an estimated 31% of those needing ART in resource-poor countries are currently receiving it [4].

The need for better education, better treatment access, better prevention programs, and better prevention technologies is therefore clear. While an AIDS vaccine may hold the best hope for ending the HIV epidemic, the development of an effective AIDS vaccine has proved to be extremely challenging. Only three HIV vaccines have advanced into efficacy testing. Two were shown not to be effective, while the third is currently being tested in a phase 3 trial [5]. Topical microbicides are also under development to prevent HIV infection, but to date none has been shown to be effective [6]. In contrast, antiretroviral therapy for HIV has been very successful with increases in life expectancy on par with uninfected populations, at least in regions where ART is widely available [7]. Antiretroviral drugs have also been used successfully in prevention of mother to child transmission (MTCT) and post-exposure prophylaxis (PEP) [4]. These successes led researchers to question the potential for antiretroviral drugs to prevent HIV transmission.

Using antiretroviral drugs as chemoprophylaxis to prevent HIV has been termed ‘pre-exposure prophylaxis’ or PrEP. PrEP would entail HIV-uninfected persons, who are at-risk for exposure to HIV, taking a regular dose of one or more antiretroviral drugs to reduce their risk for acquiring HIV. There are several lines of evidence to suggest that PrEP may be effective in preventing HIV. First, some newer antiretroviral drugs in the nucleoside reverse transcriptase inhibitor class (NRTI) have favorable safety profiles, good drug levels in the genital tract and long intracellular half-lives, all of which could make prevention of sexually transmitted HIV feasible. Second, mother to child transmission rates can be dramatically reduced when antiretroviral drugs are given to the mother prior to labor and continued in the infant. Third, observational studies of post-exposure prophylaxis after occupational exposure to HIV suggest that antiretroviral drugs can reduce the risk of HIV transmission. Fourth, animal models using antiretroviral drugs to prevent simian immunodeficiency virus (SIV) or simian-human

immunodeficiency virus (SHIV) infection have shown efficacy. These supporting rationales for PrEP are discussed in detail below.

3.1 Rationale for Pre-exposure Prophylaxis (PrEP) to Prevent HIV

3.1.1 Prevention of mother-to-child transmission (PMTCT) of HIV

Antiretrovirals have been used successfully to prevent vertical transmission of HIV from mother to child. A single dose of nevirapine given to the mother at the onset of labor and another dose given to the infant at birth resulted in a 13.1% transmission rate compared to 25.1% transmission with a regimen of intrapartum zidovudine followed by neonatal zidovudine for one week [8]. In addition, newborns who received zidovudine within 48 hours of birth had an HIV incidence of 9.2% compared to 25.2% in an untreated control group [9]. These findings were duplicated in several other studies of MTCT, providing a rationale to explore antiretroviral therapies for prevention of sexual HIV transmission [10].

3.1.2 PEP to prevent HIV infection

Post-exposure prophylaxis (PEP) with antiretrovirals has been shown in observational studies to prevent occupational transmission of HIV and is widely recommended [11]. In a case control study, zidovudine after occupational needlestick exposure reduced HIV acquisition by 81% [12]. In many countries including the US, PEP is also recommended to prevent non-occupational transmission in selected circumstance [13-19]. However, there are no controlled clinical trials of PEP to prevent sexual transmission of HIV, and PEP failure in this setting is not uncommon most likely due to treatment delay and not having systemic levels of antiretrovirals soon enough after HIV exposure [20].

3.1.3 Animal studies

Animal models have shown efficacy of both PEP and PrEP regimens. Macaque models using SIV or chimeric SHIV challenges have been used to evaluate the potential for PEP with various antiretroviral drugs at various doses. The success of PEP in these models depended on the challenge virus, route of challenge, the antiretroviral agent, dose and timing of treatment after exposure [21-25]. For example, tenofovir partially prevented SIV_{mne} infection by intravenous challenge when given within 24 hours of exposure and continued for 10 days. However fewer macaques were protected with a 10 day course compared with a longer 28 day course. [22]. Tenofovir protected macaques exposed to a vaginal SIV challenge when initiated within 36 hours, but delaying treatment to 72 hours led to one failure [24].

Several studies in macaque models have shown that ART administration before exposure prevented infection [21,26,27]. However, similar to the PEP animal models, the success of PrEP in animal models varies depending on the challenge virus and route, and the antiretroviral drugs and doses used. Repeated low-dose vaginal and rectal mucosal challenge models using lower viral titers and multiple challenges have been developed to better reflect the biology of human HIV exposure.

Initial studies of the rectal challenge model showed that oral tenofovir disproxil fumarate (TDF) delayed $SHIV_{SF162P3}$ infection, but after repeated exposure only 1 of 4 animals was left uninfected [28]. A subsequent study investigated the efficacy of single or combination antiretrovirals in the repeat-exposure model over a 14 week period, with weekly rectal challenges. Macaques were treated once daily with either (1) emtricitabine (FTC) subcutaneously, (2) FTC and TDF orally, or (3) FTC and TDF subcutaneously but with tenofovir at higher than the equivalent human dose. The risk of infection in groups 1 and 2 was lower than in untreated animals, however 100% protection was seen in group 3.. This study also compared daily PrEP to an intermittent regimen. A fourth group of animals was given the same regimen as group 3 but only at 2 hours before and 24 hours after each weekly virus challenge. All 6 animals in this group were also protected [29]. The study suggests that while single drug daily PrEP reduced infection rates, higher levels of protection may require combination drugs and higher doses. It also showed that intermittent PrEP protects as well as daily PrEP in this animal model. Despite the limitations of the animal models and small numbers of animals in these studies, the data support further evaluation of PrEP regimens in humans for prevention of HIV.

3.1.4 Favorable properties of tenofovir and emtricitabine

Favorable pharmacologic properties of more recently developed antiretrovirals have also contributed to the rationale for PrEP in humans. Several criteria for ideal PrEP drugs have been described, including a good safety profile, ease of use, a low rate of drug-drug interactions, a long intracellular half-life, mechanism of action prior to HIV integration, high levels in genital tissues, high genetic barrier to drug resistance and unique resistance profile, and cost-effectiveness [30]. The two drugs currently under study for PrEP in human trials are the nucleotide reverse transcriptase inhibitor tenofovir disoproxil fumarate alone and in combination with the nucleoside reverse transcriptase inhibitor emtricitabine as FTC/TDF. FTC/TDF will be evaluated in this study. Both drugs fullfill most of the following criteria:

Safety profile, ease of use, drug-drug interactions, intracellular half life
Both TDF and FTC have minimal side effects when used in HIV-infected subjects [31]. FTC and the closely related drug lamivudine are considered to be by far the most tolerable of current antiretrovirals [31]. TDF has been associated with renal dysfunction in some HIV-infected subjects, which is detailed further in section 3.3 and 3.4. Both drugs are formulated for once daily oral dosing as individual drugs and together as a combination fixed-dose single tablet for daily oral dosing (FTC/TDF). Neither drug has significant drug-drug interactions with non-antiretroviral drugs, and neither interacts with hormonal contraceptives [31-33]. Both have long half-lives in plasma and peripheral blood mononuclear cells (PBMCs). FTC is phosphorylated by intracellular enzymes to its active form FTC 5'-triphosphate which has an intracellular half-life in PBMCs of 39 hours [34]. Tenofovir is also phosphorylated intracellularly to its active form tenofovir diphosphate which has an intracellular half-life in PBMCs of ≥60 hours [35]. In addition, tenofovir diphosphate inhibits HIV replication

in macrophages and other non-dividing cells, which may be important in preventing HIV infection in mucosal tissues.

Mechanism of action, levels in genital tissues

Both drugs are competitive inhibitors of HIV-1 reverse transcriptase that inhibit HIV replication before integration of the proviral DNA into the host cell genome [33]. This mode of action may be important in preventing HIV as integration of viral genome into the host cell allows future expression of infectious virions causing infection. Both drugs also have high concentrations in the genital compartment at the sites of virus entry into the body. In men, genital tract TDF levels are 5 times higher than levels seen in plasma, and levels of lamivudine, which is structurally almost identical to FTC, are 6 times those in plasma [36,37]. In women, TDF and FTC genital fluid levels are at least 4 times greater than in plasma [37]. This concentration in the genital tract is in contrast to other NRTIs and most non-nucleoside reverse transcriptase inhibitors and protease inhibitors which have poor genital tract penetration. In addition, the active drug, the phosphorylated intracellular form of tenofovir, has been measured in cells in semen. Tenofovir diphosphate concentrations are 5 times higher here than in PBMCs [38]. Data are not available for FTC levels in semen, but the closely related drug lamivudine has intracellular concentrations in semen similar to PBMCs [39]. Intracellular concentration data for the female genital tract are currently under investigation [40].

Genetic barrier to drug resistance, unique resistance profile

Mutations in the HIV RNA develop over time when the virus replicates in the presence of an antiretroviral drug. Different mutations are associated with different drugs, and resistance depends on the types and patterns of mutations. A high genetic barrier to drug resistance means that multiple mutations would be required to cause drug resistance and virologic failure. Although FTC and TDF are both NRTIs, they have unique resistance profiles. The mutation M184V causes drug resistance to FTC [41]; however, this mutation does not confer significant cross-resistance to other drugs in the NRTI class except the closely related drug lamivudine. Moreover, the activity of some other drugs in the NRTI class, including TDF, is slightly enhanced in the presence of M184V [42]. In contrast, HIV develops resistance to TDF primarily through the K65R and T215Y mutations [42]. These mutations are associated with cross-resistance to other drugs in the NRTI class, but typically develop more slowly than the M184V mutation. TDF therefore has a higher genetic barrier to drug resistance, but with a less unique resistance profile. Combining FTC and TDF will increase the genetic barrier to resistance and increase the probability of preventing HIV infection.

3.2 Preclinical data

Data on the efficacy of PrEP in animal models, including intermittent dosing with TDF and FTC, are described in section 3.1.3. Preclinical toxicology information on TDF and FTC alone and in combination as FTC/TDF is detailed in the Investigational Brochures. In brief, bone toxicity was observed in rats, dogs and monkeys when TDF was administered to animals at exposures >= 6 fold human

exposures. Renal toxicity was also seen in 4 animal species at TDF exposures 2-20 times that of humans [43]. There were no significant effects seen in carcinogenesis, mutagenesis and fertility studies of TDF, and mutagenesis and fertility studies of FTC [41,43]. Long-term carcinogenesis studies of FTC are still ongoing. Reproduction studies of both drugs in animals found no evidence of fetal harm [41,43]. No exacerbation of target organ toxicity or mutagenicity was apparent when both drugs were administered together, compared with each agent alone [33]. Studies in rats have demonstrated that TDF is secreted in milk. It is not known whether TDF or FTC are excreted in human milk [33].

To further investigate the impact of TDF on bone health, researchers gave daily TDF to 32 rhesus macaques for prolonged periods, from 1 -13 years. At a TDF exposure several fold higher than seen in humans taking the usual oral TDF dose, 300mg, prolonged TDF was not associated with renal toxicity or bone toxicity as measured by urinalysis, serum chemistry, and bone mineral density. At this dosage, plasma and intracellular concentrations were similar to those seen in TDF-treated humans. No new toxicities were observed [44].

Other drugs in the NRTI class have been associated in humans with lactic acidosis and hepatitis thought to be caused by drug-induced mitochondrial dysfunction. To address the potential for TDF and FTC to cause mitochondrial dysfunction in vitro assays were conducted. Both drugs, whether administered alone or in combination, demonstrated a low potential for interfering with mitochondrial function [33].

3.3 Clinical data

3.3.1 Clinical data in HIV-infected persons

TDF was approved by the U.S. Food and Drug Administration in 2001 as a treatment for HIV infection. FTC/TDF, the TDF plus FTC combination pill, was approved for use as an HIV treatment in 2004. More than 200,000 HIV-infected people around the world have now used these drugs. In HIV-infected persons, TDF and FTC have both been studied as components of multi-drug regimens and were found to be safe and effective in treating HIV infection by lowering HIV viral load [41,43]. They have relatively low levels of side effects and slow development of associated drug resistance, compared with other antiretroviral drugs. They are also among the most convenient antiretrovirals, dosed orally once a day with or without food. Because TDF and FTC were developed for treatment of HIV infection, the large majority of clinical safety data is from HIV-infected patients, who may have symptoms due to their HIV-infection or associated conditions, and who use the drug(s) in combination with other antiretrovirals and HIV-related medications with their own side effect profiles. Therefore, safety data from HIV-infected patients may not represent the safety profile in HIV-uninfected persons.

FTC adverse reactions

FTC is generally well-tolerated in HIV-infected patients. Adverse events were reported with similar frequency in FTC treated HIV-infected groups and control treatment groups, except for skin discoloration which was more common in the FTC treated group. This skin discoloration was

generally mild and asymptomatic hyperpigmentation of the palms and/or soles. Very rare adverse events include lactic acidosis with hepatic steatosis which can occur with use of any NRTI. The following laboratory abnormalities were reported in 1-12% of HIV-infected patients receiving FTC: elevations of ALT, AST, bilirubin, creatine kinase, pancreatic amylase, serum amylase, serum glucose, serum lipase and triglycerides; decreased neutrophils [41].

TDF adverse reactions
TDF is generally well-tolerated by HIV-infected patients. Adverse events that occurred in >5% of HIV-infected patients receiving TDF with other antiretroviral agents in clinical trials included: headache, nausea, diarrhea, vomiting, rash and depression. The following laboratory abnormalities were reported in 2-12% of patients receiving TDF: elevated ALT, AST, creatine kinase, serum amylase, urine glucose, serum glucose, serum triglycerides, total and cholesterol; hematuria; decreased neutrophils. Very rare adverse events include lactic acidosis with hepatic steatosis with use of any NRTI [43].

FTC/TDF adverse reactions
No new patterns of adverse events were identified with the FTC/TDF combination. There was no increase in the frequency of previously established toxicities [33].

Bone effects of TDF in HIV-infected patients
Both osteopenia and osteoporosis are associated with HIV infection, more so among patients being treated with ART [45]. In a study comparing a TDF-based regimen with another ART regimen, TDF was more likely to be associated with small decreases in bone mineral density and increased biochemical markers of bone turnover. However these changes were small, non-progressive and of uncertain clinical significance. The effect of these changes on long-term bone health and future fracture risk are unknown. Almost all bone fractures in the study were due to trauma, and were less common in the TDF arm than the placebo arm [46]. Another study documented 455,392 patient-years of exposure to TDF from the manufacturers post marketing safety database and safety profiles from 10,343 patients enrolled the manufacturer's expanded access program. Bone fractures were rarely reported (<0.1%) [47]. The manufacturer recommendations for HIV-infected patients taking TDF are to consider bone monitoring in patients who have a history of pathologic fractures or are at risk for osteopenia [43]. FTC has no known effect on bones.

Renal toxicity of TDF in HIV-infected patients
TDF is generally well-tolerated; however the potential for renal toxicity has been suggested by case reports of proximal renal tubular dysfunction with or without a Fanconi-like syndrome in HIV-infected patients taking TDF. These cases had combinations of increased creatinine and decreased creatinine clearance, hypophosphatemia, proteinuria, and glycosuria without hyperglycemia starting from 5 to 16 months after starting TDF in combination with other antiretrovirals. In 11 of 13 cases

described, renal toxicity either improved or resolved after stopping the drug [48]. In a case series of 19 patients, the renal dysfunction appeared to be reversible and was associated with pre-existing renal insufficiency [49]. However, data from controlled clinical trials and cohort studies have shown little or no toxicity clearly associated with TDF in HIV-infected patients with normal baseline renal function. In 4 manufacturer-sponsored controlled clinical trials, there were no significant differences in renal abnormalities between the TDF and control groups over 44 weeks, 48 weeks, 96 weeks, and 144 weeks of therapy [50-55]. In the post marketing safety database and expanded access program for TDF, serious renal adverse events were observed in 0.5% of patients. Risk factors for development of renal toxicity were pre-existing renal dysfunction, concomitant nephrotoxic medications, low body weight, advanced age and low CD4 cell count [47]. Data from non-US, non-European populations also show low absolute rates of renal dysfunction in HIV-infected adults taking TDF. The Development of Antiretroviral Therapy in Africa (DART) trial, which enrolled 3314 antiretroviral naïve, HIV-infected adults in Africa, found that rates of severe renal impairment were low through 96 weeks of therapy and similar between TDF and non-TDF regimens. Sixty-five percent of patients were women [56]. A study of Thai HIV-infected patients on TDF found no significant changes in renal function through 21 weeks of therapy [57].

These data show that the incidence of clinically significant renal dysfunction in HIV-infected patients taking TDF is likely to be very low. Current recommendations from the manufacturer and expert groups for TDF management in HIV-infected patients are: TDF dose reduction for patients with renal dysfunction (creatinine clearance ≤50 mL/min), avoidance of TDF co-administration with other nephrotoxic agents, and for patients with impaired creatinine clearance (≤90 mL/min) twice yearly monitoring of renal function, serum phosphate, and urine protein and glucose [43,58].

Safety in pregnancy

There are no well-controlled clinical studies on the safety of TDF, FTC or FTC/TDF during pregnancy. All drugs are categorized as United States FDA pregnancy category B: animal reproduction studies have failed to demonstrate a risk to the fetus and there are no adequate and well-controlled studies in pregnant women. They are recommended to be used during pregnancy in HIV-infected women only if clearly needed [33,41,43]. However data from the international registry of pregnancy outcomes in 9889 HIV-infected women taking TDF indicate that tenofovir use during pregnancy does not increase the risk of fetal anomalies and has a lower risk than many other antiretrovirals [59]. The overall birth defect prevalence in the registry (2.8%) was comparable to general population-based surveillance data (2.72%). The birth defect prevalence with exposure to TDF in the first trimester was 2.2% compared to 3.0% for all antiretrovirals.

Drug resistance
FTC resistance has been documented in some HIV-infected patients treated with FTC alone or in combination with other antiretroviral agents. One study found that 6/16 (37.5%) of treatment-naïve patients started on an FTC-containing regimen with subsequent virologic failure had the M184V mutation in the HIV reverse transcriptase gene. FTC-resistance confers cross-resistance to lamivudine but not to other NRTIs and NNRTIs in current use. The activity of some other drugs in the NRTI class, including TDF, is slightly enhanced in the presence of the M184V mutation [33].

TDF resistance has also been documented in some HIV-infected patients treated with TDF in combination with other antiretroviral agents. Eight of 47 (17%) of treatment-naïve patients started on a TDF containing regimen with subsequent virologic failure had the K65R substitution mutation in the HIV RT gene. In treatment-experience patients, 14/304 isolates from patients failing a TDF-containing regimen showed the K65R substitution mutation [33]. TDF-resistance with the K65R mutation confers some degree of cross-resistance to lamivudine, FTC, didanosine, stavudine and abacavir, all in the NRTI class.

Resistance to the combination of FTC and TDF has been selected in vitro. The viral isolates had the M184V and/or K65R substitutions in the HIV RT gene. These mutations are associated with cross-resistance to other antiretroviral drugs as described above [33].

Two aspects of antiretroviral drug resistance are relevant for studies of PrEP. First, if drug resistance to TDF and/or FTC is common in HIV-infected persons in the region, then PrEP regimens using these drugs may fail to prevent transmission of HIV. Second, if HIV-uninfected persons using PrEP unknowingly become HIV-infected and continue taking PrEP, there is potential for development of drug resistance mutations that will compromise their ability to use certain antiretrovirals for HIV treatment in the future.

The rates of drug resistance to TDF and FTC in circulating viruses in African populations are not well-known. Published reports have suggested drug resistance in transmitted viruses, termed primary resistance, in ART-naïve Africans has been uncommon [60-64]. However access to ART in the developing world is rapidly increasing, and studies in the US and Europe showed an increase incidence in primary drug resistance mutations following the introduction of ART [65-67]. Data from a recent study of 211 acute or recent HIV infections in antiretroviral naïve subjects in Kenya, Rwanda, Uganda, Zambia and South Africa found 5% with drug resistance mutations. However, no M184V and K65R mutations were identified (unpublished data, IAVI).

The development of drug resistance mutations in individuals taking PrEP who become HIV-infected will likely depend on: (1) the rate of resistance in circulating virus, described above; (2) the potency, duration and intracellular and mucosal distribution of the drugs selected for PrEP; (3)

the number of active drugs in the PrEP regimen; and (4) the duration which the individual continues taking PrEP after becoming HIV-infected. FTC/TDF containing two active drugs may reduce the risk of developing drug resistance mutations. It is also important to also consider the efficacy of the PrEP regimen; more efficacious regimens will lead to fewer HIV infections and therefore reduce the risk of drug resistance developing. The impact of using a single agent, such as TDF, for PrEP on the acquisition or development of drug resistance has been modelled for the Botswana PrEP trial [68]. In this trial design, if 600 participants receive PrEP with TDF, 45 can be expected to become HIV infected. Of these, less than one participant would be expected to either acquire or develop the K65R tenofovir resistance mutation. The large majority of K65R mutations would be generated within the HIV treatment program in the country, not from the potentially small number of seroconvertors in effective PrEP programs.

Effect on hepatitis B co-infection
Both TDF and FTC have activity against hepatitis B virus (HBV). TDF is approved in many countries for treatment of chronic HBV infection, as is lamivudine. However FTC is not yet approved for this indication. Treatment of HIV and HBV co-infected patients with TDF, FTC or FTC/TDF typically lowers HBV viral load. Discontinuation of these drugs may cause acute exacerbation of hepatitis with or without hepatic decompensation. In addition, single drug treatment of hepatitis B infection can lead to drug resistance, particularly with lamivudine, and can also cause an acute exacerbation of liver disease. To avoid development of HBV resistance, it is recommended not to use a single agent for treatment of HBV in HIV co-infected patients. In addition, upon discontinuation of these drugs, liver function should be monitored and treatment with another agent active against HBV could be initiated to prevent flares [31].

3.3.2 Clinical data in HIV-uninfected persons

Safety and efficacy data on TDF, FTC and FTC/TDF in HIV-uninfected persons are limited. One phase 2 clinical trial of PrEP has been completed in Ghana, Cameroon and Nigeria. 936 high-risk women were randomized in a 1:1 ratio to TDF 300 mg daily or placebo for 12 months of follow-up [69]. No difference in adverse events was seen between groups. Laboratory safety data were analyzed from Ghana and Cameroon only with 210 person-years of follow-up. No difference between groups in grade 3 or 4 laboratory abnormalities was seen. Among the 56 women who tested positive for chronic HBV infection, there was no difference between groups in liver transaminase levels before or after discontinuation of study drug. Patients known to have chronic HBV infection were followed for 3 months after study drug discontinuation. The rate of liver transaminase abnormalities during this period was not increased in the TDF group. However, only women with normal baseline liver transaminase levels and no signs of symptoms of advanced liver disease were enrolled in the study. These data in HIV-uninfected African volunteers suggest no increased safety risk with daily TDF treatment.

Due to premature closures of the Cameroon site and the Nigeria site, the planned duration of follow-up and study power was not achieved to evaluate efficacy. Fewer HIV seroconversions were seen in the TDF group than placebo, 2 (0.86 per 100 person-years) vs. 6 (2.48 per 100 person-years), for a rate ratio of 0.35 (95% confidence interval 0.03-1.93) which was not statistically significant. Of the 2 seroconversions in the TDF group, specimens were available from one for genotypic analysis which showed no drug resistance mutations. After study drug was discontinued in Cameroon, another 6 seroconversions were observed, 4 in the TDF group and 2 in placebo.

Behavioural risk did not increase during the trial. The average number of coital partners per week declined from 21 to 14 during the trial. Self-reported condom used increased from 52% to 95%.

Several trials are currently investigating the safety and efficacy of daily TDF or FTC/TDF for PrEP in various populations. Since 2005, the US Centers for Disease Control and Prevention (CDC) has been conducting an extended safety trial in US to assess the clinical and behavioral safety of once-daily TDF among 400 HIV-uninfected men-who-have-sex-with-men (MSM). This trial will not be large enough to evaluate efficacy. Results are expected in 2009 [70]. The CDC is also conducting a safety and efficacy trial of TDF in 2000 HIV-uninfected intravenous drugs users in Thailand. The trial started in 2005 and results are expected in 2009 [71]. Another CDC-sponsored safety and efficacy study of PrEP started in Botswana in 2007. This study is evaluating FTC/TDF in 1200 HIV-uninfected heterosexual men and women, age 18-29. Results are expected in 2010 [71].

The Pre-Exposure Prophylaxis Initiative (iPREX) study, sponsored by the US National Institutes of Health (NIH) and the Bill and Melinda Gates Foundation (BMGF), is evaluating daily FTC/TDF to prevent HIV in MSM populations in Peru, Ecuador, South Africa, Thailand and the US [71]. The trial started in 2008 and results are expected in 2010. The Partners PrEP study, also sponsored by the NIH and BMGF, is evaluating daily TDF or daily FTC/TDF in 3900 HIV-discordant heterosexual couples in Uganda and Kenya. The trial started in 2008 and results are expected in 2012 [71]. The FEMPrEP trial, sponsored by Family Health International and BMGF, is evaluating daily FTC/TDF in 3900 at-risk women in Kenya, Malawi, South Africa and Tanzania. The study is expected to start in 2008 and results are expected in 2012 [71]. Finally, the Microbicide Trials Network of the NIH is evaluating daily oral TDF or daily FTC/TDF or daily TDF vaginal gel in 4200 sexually-active women in Southern Africa in the VOICE study. The study is expected to start in 2009 and results are expected in 2012 [71]. Of note, all of these trials are evaluating daily dosing only.

3.3.3 Adherence to PrEP

Successful PrEP will likely require sustained adherence. Whether the high levels of ART treatment adherence seen in resource-limited settings [72-79] will be replicated in a prevention setting is unclear. Incomplete or poorly characterized adherence has been a major challenge in recent HIV prevention trials. In the absence of clear and unequivocal results, it has been impossible to determine if incomplete protection of recent biomedical interventions is due to “user failure” or “method failure.” Unlike adherence to HIV treatment, little is known about the level, pattern, and correlates of adherence to HIV biomedical prevention strategies such as PrEP. A better understanding of PrEP adherence behaviour will be essential to interpret clinical trial findings and maximize the effectiveness of PrEP. In particular, understanding adherence to an intermittent PrEP regimen and its relationship to the timing of sexual activity will be important to evaluate the feasibility and potential efficacy of intermittent dosing schedules.

3.3.3.1 Adherence data from biomedical HIV prevention trials

Recent microbicide trials suggest that adherence to coitally-dependent topical gels is sub-optimal. Adherence to the microbicide carageenan was 65% of sex acts by self-report and 30% of sex acts using a dye test of returned applicators [80]. In a study by Padian, the female diaphragm demonstrated no reduction in HIV acquisition when compared to condoms alone. The interpretation of the efficacy of this intervention is complicated by the fact that only 70% of women reported using the diaphragm during their last sex act [81].

Early data suggest that adherence to oral preventive therapy is also challenging. In the phase II trial of PrEP among female sex workers in West Africa, adherence to daily TDF adherence was 74% [69]. This first PrEP trial illustrates the challenges of adherence to a daily oral medication and the need for a better understanding of PrEP adherence.

Recent results from completed trials of acyclovir suppressive therapy against genital herpes as a method for HIV prevention similarly suggest substantial variability in adherence. Among high-risk women in Tanzania, only 50% of participants took >90% of pills, as assessed by 3-monthly clinic-based pill counts [82]. Overall, there was no difference in genital ulcer disease between the acyclovir and placebo groups, a marker of genital herpes reactivation, which should be substantially suppressed by acyclovir. This poor response suggests that adherence may have been even less than recorded by clinic-based pill count. HSV DNA (detected by periodic genital sampling) was less common in the acyclovir than the placebo group in those reporting at least 90% adherence to treatment during the preceding 3-month period (5.0% for placebo vs. 1.7% for acyclovir), suggesting that adherence was an important determinant of treatment effectiveness. Self-reported adherence was considerably better (mean of 87%) in the multi-center HPTN 039 trial among African women and MSM in the Americas [83]. In that trial, participants received

monthly adherence counselling, including clinic-based pill counts and weekly reminder pill boxes which may have aided adherence. Similarly, high self-reported adherence to the study drug taken twice daily (acyclovir or matching placebo) has been observed among the HIV-infected partners within HIV-discordant couples in the trial of herpes suppression to prevent HIV transmission ("Partners in Prevention", led by Dr. Connie Celum in 14 African sites) with 81% having taken ≥90% of dispensed study drug, based on monthly pill counts and self-reported adherence data [84]. While these levels of adherence are encouraging, clinic-based pill counts are unreliable [85-89] and self-report predictably overestimates adherence by as much as 20% [90,91]. Participant reported measures may be especially problematic in highly structured clinical trial settings where there is a high level of overreporting due to social desirability bias.

In summary, results from HIV prevention trials collectively demonstrate the need for accurate and objective adherence measurement to interpret biologic efficacy. In the absence of multiple objective measures, the true level of adherence is uncertain and the extent to which adherence contributes to negative study findings is unknown. This pilot study will use two objective measures of adherence to intermittent PrEP: electronic medication monitoring using the medication event monitoring system (MEMS TrackCaps) and intracellular and plasma drug levels (see 3.3.3.2.).

MEMS TrackCap is a medication bottle cap with an embedded microchip to detect and record the date and time when the bottle is opened. Data can be uploaded wirelessly from the MEMS TrackCap to a MS-Windows based computer using a palm-sized reader. Each MEMS TrackCap has a unique serial number, can store up to 3800 events, and has a 36 month battery life. MEMS has been used successfully in ART trials in various types of resource settings. The MEMS time/date stamps create an electronic record of pill bottle–opening behaviour that is closely associated with HIV viral suppression and drug resistance [92-94].

Limitations of MEMS are that it records bottle opening and not actual medication dosing. In addition, patients may take out more than one dose at a time, or "pocket doses." At other times, patients may open the pill bottle without taking the medication, called "curiosity events" [95,96]. These behaviors lead to an underestimation or overestimation of actual adherence, respectively. The MEMS-based adherence rate can be adjusted to account for pocket doses and curiosity event, if the individual records and conveys such information to the study staff. MEMS also precludes the use of medication pill-box organizers, which have been associated with modest (4%) but highly cost-effective increases in adherence in observational studies [97,98].

3.3.3.2 Antiretroviral drug levels to measure adherence

Several investigators have examined antiretroviral drug levels as a measure of adherence [99-102]. The interpretation of random drug levels to most ARTs is complicated, however, because concentrations reflect drug absorption and metabolism as well as adherence to recent doses.

Drug levels for shorter half-life medications reflect only recent adherence, which may be heavily influenced by the patient improving his/her adherence just prior to a scheduled blood draw, otherwise known as "white coat compliance [103]. Drug levels in longer half-life medications such as tenofovir and emcitrabine may be less sensitive to "white coat compliance" and may better reflect long-term adherence. The half-life of tenofovir in plasma is 17 hours and levels remain detectable out to 48 hours after a single dose [43]. The half-life of intracellular tenofovir diphosphate in blood cells is estimated to be at least 60 hours [35]. Because few studies have combined both objective behavioural adherence measures and drug levels [104], the relationship between adherence and drug level, especially to longer half-life medications is largely unknown. We propose to determine to what extent intracellular tenofovir diphosphate and emtricitabine drug levels are associated with objectively measured adherence both for daily PrEP and for intermittent PrEP. Specimen collection for drug levels poses fewer logistical challenges than objective measures of adherence behaviour. Characterizing the relationships between objectively measured adherence and drug levels could provide future studies with valid and feasible approaches to better understand how adherence impacts prevention efficacy.

3.3.3.3 Measuring adherence in an African setting

Obtaining accurate measurements of adherence in HIV prevention studies has been challenging. Most prevention clinical trials use self-reports of adherence behaviour and/or counts of returned pills/products to the study site to indirectly measure adherence. Patient report suffers from both social desirability bias and the simple imprecision in "remembering what is forgotten" [79, 90, 91, 105-110]. Counts of returned pills to clinic or study settings have been notoriously inaccurate [86, 105, 101, 106-110]. While there is no "gold standard" adherence measure, much has been learned about ART adherence using multiple objective measures of adherence [7, 91-98,110,117-126]. In Uganda, Bangsberg and colleagues have studied MEMS, unannounced home pill count and structured self-report to measure ART adherence in HIV-infected patients [72, 79, 94, 127]. This experience studying adherence is among the most extensive in Africa. Among 97 HIV-infected patients purchasing generic ART, all three adherence measures were closely correlated with each other (R=0.81), and adherence of >80% by all measures was associated with viral load suppression [77]. In this study there was no significant difference between patient-reported and objective measures of adherence, however the extremely high adherence rate, 91-94%, suggests the study population may be highly motivated to treat their HIV disease. Similarly, in a study of 174 patients on ART in Uganda, there was a high degree of longitudinal correspondence between MEMS, unannounced home pill count and self-report, and complete viral load suppression was more common in those with high levels of adherence by all measures [94].

3.3.3.4 Relationship between sexual activity and adherence to HIV biomedical prevention methods.

Biologic efficacy will require that PrEP recipients have therapeutic ART drug levels at the time of sexual exposure or immediately afterwards. Studies of post-exposure prophylaxis suggest that prevention efficacy declines with the time between exposure and ART initiation [127]. Therefore, a detailed understanding of patterns of PrEP adherence and specifically how PrEP is used with respect to sexual behavior will be important to understand PrEP efficacy and guide behavioral counselling regarding optimal use.

Interactive voice response (IVR) systems have been developed to measure daily health behaviours [128-130] including alcohol use [131-133], depression [134, 135], obsessive compulsive disorder [136], smoking cessation [137, 138], gambling [139] and sexual behaviour [140]. With IVR, an automated system calls the participant's cell phone daily. The participant answers a brief set of questions by touching numbers on the telephone keypad. The lack of a human interviewer may decrease the social desirability bias inherent in person-to-person interactions, and allows for frequent data collection.IVR is currently under evaluation in Uganda to measure self-reported ART adherence [Bangsberg, unpublished]. Cell phone text messaging (SMS) has also been used successfully in an ART adherence program in the US to enhance adherence, although not to collect data [Dimagi, Charlestown MA]. The reliability and high penetration of cell phone use in Kenya makes IVR and SMS feasible.

This study will determine the relationship between sexual activity and PrEP adherence by mapping sexual behavior with daily IVR or SMS self-report and PrEP adherence with daily MEMS data. The method used, IVR or SMS, will be determined based on availability in each country. Daily assessment of sexual behavior combined with daily electronic adherence behavior is required to determine which individuals 1) sustain PrEP adherence per protocol, 2) only use preventive antiretroviral therapy according to the fixed intermittent schedule, 3) only use it just prior to sex, or 4) use it only after sex as post-exposure prophylaxis.

Because deployment of IVR or SMS to collect data in Kenya is a new procedure and not without risk of technical, logistical, or cultural obstacles, this pilot study will assess feasibility and participant burden. Feasibility will be assessed by percentage of study days with missing data and participant burden will be assessed by 5 point Likert scale. It will also determine if IVR is more sensitive at detecting sexual behavior than a 30-day timeline follow-back procedure. In the event that IVR is not practical, the 30-day timeline follow-back interviewing can be used as the primary sexual behavior measure [141, 142].

Impact of daily measurements on sexual behaviour. Daily measurements may change sexual behaviour. In a recent study of IVR in 44 Hispanic students, there was a modest temporary decrease in sexual behavior

self-reports over time from an average of 0.375 sexual episodes per day during the first three weeks of IVR reporting to 0.25 episodes per day over 9 weeks and an increase towards baseline at the end of the 13-week period [140]. Similar temporary reductions have been reported with IVR measurement of alcohol consumption. Helzer, for example, reported an average decline in alcohol consumption of about 19% over a two year period in a study of daily self-reporting using IVR [137]. In sum, daily self-reports bear the risk of reactivity either in form of socially desirable responding or as a true effect on behaviour. However, these changes may be temporary, and the increase in measurement precision in knowing the extent to which PrEP adherence is associated with sexual behaviour likely outweighs the potential impact on behaviour.

3.4. Preclinical and clinical data on immune response with PrEP

Several animal studies using PMPA, tenofovir monophosphate, as PEP to prevent SIV infection found higher rates of SIV-specific immune responses in the PMPA treated macaques that were protected from SIV or that controlled their infection [144-150]. In some studies, protected animals were more likely to be protected or to control infection when rechallenged with a homologous or heterologous virus later after a drug-free period, than animals that had not been treated with PMPA [144-149]. Most of these studies used less virulent SIV strains such as SIVsmE660, SIV8980 and SIVmac055 [144-148]. Investigators hypothesized that drug suppression of viral replication during acute infection allowed an effective immunologic sensitization and that cytotoxic T lymphocytes led to either protection on rechallenge or sustained host control after drug withdrawal [151]. In a study of topical tenofovir to prevent rectal transmission of SIV in animals, SIV gag-specific IFN-γ T cell responses were seen in 4 of 7 protected animals tested [152]. However the numbers of animals in these studies were small. In addition, in a subsequent experiment using PMPA as PEP to prevent SIV and using a highly virulent strain of SIV (SIV_{mac239}), there were no measurable anti-SIV cellular responses and no animals were protected from rechallenge [153].

Very few clinical data exist to support the concept that effective suppression of viral replication by PrEP during exposure/acute infection may allow the development of effective immune responses leading to either protection or control of HIV infection. In a case report, a patient who used FTC/TDF as PEP and seroconverted was found to have an attenuated clinical course with delayed seroconversion, persistently low levels of plasma viremia and sparing of gut mucosal CD4+ T cells which are a primary target for HIV [154]. The patient, an HIV-uninfected male who reported MSM risk behaviour, was given a course of daily FTC/TDF therapy as PEP the day after reporting multiple unprotected sexual contacts. The course was extended to 6 weeks after he reported additional unprotected contacts. One month later, a second 4 week course of FTC/TDF was given after another episode of unprotected sexual contact. Midway through the second 4 week course, the patient tested HIV-positive by EIA. Initial HIV viral loads were very low, 213 and 647 copies/mL, probably reflecting viral suppression by FTC/TDF. Once off drug, viral load remained low with a peak below 10,000 copies/mL and set point viral load at 6 months of approximately 3,000 copies/mL. The degree of immune dysfunction also

appeared to be attenuated with CD4+ count of 800 cells/mm^3 at 6 months, and relative sparing of GI mucosal CD4+ cells on biopsy. No patient or viral factors were found to explain the attenuated course of acute infection. HLA typing did not show B57 or B27, and the patient was not heterozygous for the CCR5-delta 32 deletion. Significantly, despite continuing FTC/TDF after the point of HIV infection, no drug resistance mutations were identified on genotyping. Although FTC/TDF prophylaxis did not prevent HIV infection, this case suggests attenuated acute infection due to FTC/TDF prophylaxis and perhaps delayed disease progression.

3.5 Mitigation of risks

Potential risks of adverse reactions, renal toxicity, bone toxicity, reproductive toxicity, exacerbation of hepatitis B infection, and drug resistance are described above. These risks will be minimized as follows: Individuals will be followed closely during the trial for development of clinical or laboratory abnormalities. Individuals with baseline renal dysfunction will be excluded, and renal function will be closely monitored, using estimate creatinine clearance, during the trial. The risk of bone toxicity will be minimized by the short duration of follow-up on drug: 4 months. Women who are pregnant, lactating or desiring pregnancy in the next 4 months will be excluded. Women of reproductive capacity must agree to use an effective form of contraception during the study to minimize the risk of pregnancy. Individuals with evidence of chronic hepatitis B (HBsAg-positive) will be excluded. The potential for development of drug resistance in volunteers who become HIV-infected during the trial will be minimized by frequent HIV testing and immediate cessation of investigational product if HIV infection is diagnosed. Very few HIV infections are expected given the small sample size and short follow-up period. If the annual HIV incidence is at most 10% in Kenyan volunteers, we could expect <2 incident HIV infections among 72 volunteers enrolled.

3.6 Other potential risks

Study volunteers may increase their risk-taking behaviour, including decreasing condom use, if they believe the study drug is protecting them, despite education that the trial includes a placebo and that the study drug is not known to be effective. As described above, behavioural disinhibition was not seen in the daily PrEP trial in women in West Africa. The average number of coital partners per week declined from 21 to 14 during the trial. Self-reported condom used increased from 52% to 95% [69]. The concern for behavioural disinhibition has been raised not only in PrEP research, but in other biomedical HIV prevention trials – such as vaccine and microbicide studies. Data from microbicide studies in similar populations suggest that the risk of behavioural disinhibition is not significant enough to impact HIV transmission risk and, in general, self-reported condom use increases during trial participation. For example, in a recent phase 3 microbicide trial in Africa and India, condom use increased from approximately 60% before the trial to 96% during the trial with all partners, and 75% with regular partners. The median number of partners did not change [155]. In a microbicide trial in South Africa in which women were instructed to use both condoms and gel during sex, there was an equal number of HIV infections in each arm. During the trial condom use increased to more than 50% of sex acts, and the number of reported partners was unchanged [156].

3.7 Study Rationale

Almost 18,000 people are expected to be enrolled in trials of daily PrEP with TDF or FTC/TDF over the next 3 years. However, if daily PrEP is shown to be effective, it is highly probable that uptake may be limited due to cost and acceptability of daily dosing in various populations. In addition, even in populations with access to daily PrEP, at-risk persons may choose to use PrEP on an intermittent basis, such as prior to high-risk or unprotected sex. This practice is already being seen in certain US and European at-risk populations. The pharmacokinetics of TDF and FTC support less than daily dosing, as does the efficacy of intermittent TDF and FTC in the low-dose repeat rectal macaque challenge model. Intermittent PrEP regimens should be evaluated in key at-risk populations. Data on adherence and intracellular drug levels achieved with intermittent PrEP regimens should be explored. If daily PrEP shows some level of efficacy in current trials, evaluating the equivalence of intermittent regimens could be the next step in PrEP development. However, a direct comparison of daily and intermittent regimens for efficacy may be difficult due to very large trial size requirements for equivalence or non-inferiority trials. Direct comparison could be carried out in smaller trials, and bridging data showing equivalent intracellular drug levels with daily and intermittent regimens could provide important data to advance intermittent PrEP.

This pilot study will compare daily to intermittent PrEP with FTC/TDF in MSM and female sex workers (FSW) at two research centres in Kenya. Safety, adherence and drug levels, both plasma and intracellular, will be compared. The intermittent regimen will be one pill every Monday and Friday and within 2 hours after sex, with no more than one dose per day. This drug combination was shown to be 100% effective in preventing infection in an animal model when given 2 hours before and within 24 hours after exposure, and was more effective than either single drug alone [29]. In addition, a two drug regimen may be more likely to prevent the development of drug resistance mutations in the event that a volunteer becomes HIV-infected during the study.

Study population

The participating research centres in Kenya, located in Nairobi and near Mombasa, have experience working with MSM and FSW populations. These groups are potentially appropriate populations in which to evaluate intermittent PrEP. Work conducted in MSM and female sex worker populations in anticipation of larger scale HIV prevention trials has demonstrated annual HIV incidence greater than 5% making these risk populations suitable to test biomedical interventions [85]. In Kilifi, near Mombasa, the first longitudinal study including MSM in East Africa was initiated in 2005 as part of a multi-centre effort to prepare for HIV vaccine efficacy trials [157-159]. This cohort study (IAVI Open B protocol) currently follows about 150 MSM and 60 FSW near Mombasa, with 60 MSM and 15 FSW in monthly follow-up and the remainder in quarterly follow-up. The observed annual HIV incidence was 9.6% (95% CI 6.4-14.3) for MSM, and 4.7% (95% CI 2.2-9.8) in FSWs. This cohort study is also conducted by Kenya AIDS Vaccine Initiative (KAVI) of the University of Nairobi, in Nairobi,

Kenya. The observed annual HIV incidence was 11.6% (95% CI 6.4-21.0) for MSM and 0.6% (95% 0.2-1.9) in FSWs.

For an intermittent PrEP regimen with a post-coital dose to be feasible as an intermittent regimen, sexual intercourse needs to occur less frequently than every day, otherwise the dosing would be equivalent to daily dosing. Behavioural data from 81 MSM in the Mombasa cohort revealed a median of 3 different days of sexual activity per week (IQR 2-4), and median of 2 different days on which unprotected anal or vaginal sex occurred per week (IQR 1-3) [160]. Only 3/81 (3.7%) participants reported at least one week wherein unprotected anal or vaginal sex took place on more than 5 days over a 6 week period. These data suggest that the majority of the high risk MSM could potentially be protected by an intermittent drug regimen that matches their sexual activity.

Currently no published data exist on whether PrEP in any dosing regimen is likely to be acceptable to at-risk populations in Africa. A brief formative study to evaluate knowledge, acceptability of various PrEP regimens, and willingness to participate in a PrEP study is being planned in Kilifi. In addition to obtaining data on willingness to participate in a planned intermittent PrEP study, the formative study will collect interview and focus group data from prospective study volunteers and will inform the procedures and data collection instruments in the pilot study.

Relationship between adherence, plasma drug levels and intracellular drug levels
A critical determinant of PrEP efficacy will be how varying adherence behavior causes varying drug levels over time. There is substantial variation in pharmacokinetics between individuals. The combination of daily MEMS adherence data, combined with an individual's peak-trough drug levels (plasma and intracellular) after an observed dose (enhanced with population-based estimates), provide a relatively more precise estimation of drug concentration at any point in time. Such a longitudinal pharmacokinetic model based on observed drug levels and daily adherence behavior can reveal specific periods of subtherapeutic drug exposure that places individuals at risk for HIV transmission. After achieving steady state, specimens for plasma and intracellular drug levels will be collected at the Week 4 visit. Serum will be collected immediately prior to a scheduled witnessed dose to determine Cmin and again approximately 2 hours and 24 hours after a witnessed dose to determine Cmax. This will establish the typical range of drug plasma concentrations in each volunteer defined by the peak-trough relationship.

Study summary
The study will allow for collection of important data on the feasibility of an intermittent/daily PrEP regimen in MSM and FSW populations, and it will directly compare adherence and intracellular drug levels in daily and intermittent PrEP recipients. It will also evaluate the relationship between drug adherence, sexual behaviour and intracellular drug levels with an intermittent PrEP regimen. Specifically, this study will use timed plasma and intracellular drug levels after a witnessed dose and MEMS adherence data to model drug levels. This model can explore relationships between adherence and intracellular drug levels to determine if intracellular drug levels can be used as a surrogate adherence

measure. In addition it will evaluate the relationship between adherence to an intermittent PrEP regimen and timing of sexual activity to estimate what proportion of sexual events may be 'protected' by intermittent PrEP. The pilot will use objective MEMS adherence measurement and evaluate the feasibility of newer adherence measurements such as hair sampling and plasma drug levels. The pilot will also evaluate the feasibility of using either IVR or SMS to collect sexual activity data in an African setting. It will allow study teams and communities to prepare for potential subsequent larger trials of intermittent PrEP. The pilot study is not sized to evaluate efficacy. If the pilot shows this intermittent PrEP regimen to be safe, feasible in terms of adherence, and to achieve intracellular drug levels similar to daily PrEP, these data could be used to design a larger phase 2 study with one or more intermittent PrEP regimens. The goal of such a trial would be to provide bridging data if daily PrEP regimens are found to be effective or to prepare for efficacy or non-inferiority trials of intermittent versus daily PrEP.

Investigation of immune responses associated with FTC/TDF will also be evaluated in the pilot study. The proportion of volunteers on FTC/TDF with HIV-specific immune responses will be assessed at 2-3 time points and compared to responses in volunteers assigned to placebo. Immune responses may be correlated with risk behaviour and host factors, such as HLA type. As noted above, very few HIV infections are expected to occur during the study, so correlation of HIV-specific immune responses and protection from infection or attenuation of disease progression will not be possible until a larger study is conducted.

4.0 STUDY OBJECTIVES

4.1 Primary Objectives

To evaluate the safety of daily and intermittent dosing (twice a week and within 2 hours after sex with no more than one dose per day) of FTC/TDF

To compare the acceptability of and adherence to daily and intermittent regimens

To evaluate mean intracellular drug concentrations in the daily and intermittent regimens

To evaluate the relationship between adherence and intracellular drug levels

To evaluate changes in HIV-associated risk behaviour

4.2 Other Objectives

To prepare study teams for larger PrEP trials

To evaluate methods for measuring adherence and sexual activity

To evaluate potential for medication sharing

To evaluate HIV-specific immune responses in volunteers randomized to FTC/TDF and to placebo

5.0 STUDY ENDPOINTS AND STUDY DESIGN

5.1 Study Endpoints

5.1.1 Primary Endpoints

Safety and tolerability:

- The proportion of volunteers with moderate and greater severity clinical adverse events
- The proportion of volunteers with mild, moderate and greater severity of renal toxicities, and other moderate and severe laboratory abnormalities
- The proportion of volunteers with serious adverse events

Acceptability:

- Willingness to use the study regimen, if the results from this study are shown to be effective

Intracellular drug concentrations:

- The mean intracellular drug concentration for each group assigned to FTC/TDF

Adherence:

- The proportion of volunteers, who by MEMS data, took at least 80% of expected doses of the investigational product
- The proportion of volunteers assigned to FTC/TDF who have detectable drug plasma levels within 48 hours of reported use
- The relationship between intracellular drug levels and adherence in volunteers assigned to FTC/TDF

Behavioural:

- Reported number of steady and casual sex partners
- Frequency of unprotected vaginal and/or anal intercourse
- Substance use prior to or during sex

5.1.2 Other Endpoints

- The proportion of volunteers who report somewhat high or high levels of burden in using electronic medication monitoring to measure adherence, and IVR or SMS to measure sexual activity
- The proportion of study days with missing sexual activity data
- The proportion of volunteers who report sharing study medication.
- The proportion of volunteers assigned to placebo that have detectable intracellular drug levels
- The proportion of volunteers with HIV-specific immune responses as measured by analysis of cellular or humoral immune response, or changes in gene regulation as measured by microarray or proteomic techniques

5.2 Study Design

The study is a randomized, placebo-controlled blinded study with volunteers assigned to FTC/TDF daily, FTC/TDF intermittent, placebo daily, or placebo intermittent in a 2:2:1:1 ratio. Dosing regimen (daily or intermittent) will not be blinded.

Table 1
Study Design

Investigational Product	Dosing Regimen	Route of Administration
FTC/TDF	Daily	PO
FTC/TDF	Intermittent	PO
Placebo	Daily	PO
Placebo	Intermittent	PO

This study evaluates the use of FTC/TDF (FTC 200mg / TDF 300mg) or placebo;

Daily dosing: 1 tablet orally per day

Intermittent dosing: 1 tablet orally Monday and Friday and again within 2 hours after sexual intercourse (except on Monday or Friday), not to exceed 1 dose per day.

PO = Oral

5.2.1 Duration of the Study

Volunteers will be screened up to 42 days before randomization and will be followed for 4 months after the randomization.

It is anticipated that it will take approximately 3 months to enrol the study.

5.2.2 Study Population

Healthy male or female adults 18-49 years of age who are willing to undergo HIV testing, counselling and receive HIV test results, if female to use an effective method of contraception, and who, in the opinion of the principal investigator or designee, understand the study and who provide written informed consent.

Principal exclusion criteria include confirmed HIV-1 or HIV-2 infection; pregnancy and lactation; chronic disease; confirmed chronic hepatitis B infection; clinically significant abnormal laboratory values.

Approximately 72 volunteers (24 FTC/TDF daily, 24 FTC/TDF intermittent, 12 placebo daily,12 placebo intermittent recipients) who meet all eligibility criteria will be included in the study.

5.2.3 Inclusion Criteria

1. Healthy male or female, as assessed by a medical history and physical exam
2. At least 18 years of age on the day of screening and not older than 49 years on the day of randomization;
3. Willing to comply with the requirements of the protocol and available for follow-up for the planned duration of the study;
4. In the opinion of the Principal Investigator or designee, has understood the information provided and has provided written informed consent before any study-related procedures are performed;
5. Willing to undergo HIV testing, STI screening, HIV counselling and receive HIV and STI test results;
6. At risk for HIV infection as defined by at least one of the following:
    a. Current STI or STI in the previous 3 months
    b. In the past 3 months had multiple episodes of unprotected vaginal sex
    c. In the past 3 months had multiple episodes of unprotected, receptive anal sex
    d. In the past 3 months engaged in sex work for money or drugs
7. If a female of childbearing potential (i.e., not post-menopausal or surgically sterile), using an effective method of non-barrier contraception (hormonal contraceptive; intrauterine device (IUD);

surgical sterility) from 7 days prior to randomization until the end of the study. All female volunteers must be willing to undergo urine pregnancy tests at time points as indicated in the Schedule of Procedures (Appendix A).

5.2.4 Exclusion Criteria

1. Confirmed HIV-1 or HIV-2 infection

2. Any clinically significant acute or chronic medical condition that is considered progressive or in the opinion of the investigator would make the volunteer unsuitable for the study, including severe infections requiring treatment such as tuberculosis, and alcohol or drug abuse.

3. Any of the following abnormal laboratory parameters:
   - Haemoglobin <9.0 g/dL
   - Creatinine clearance <80mL/min, as calculated by Cockcroft-Gault equation:

     CrCl in mL/min =

     Males:
     $$\frac{(140 - \text{age in years}) \times (\text{wt in kg})}{72 \times (\text{serum creatinine in mg/dL})}$$

     Females:
     $$\frac{(140 - \text{age in years}) \times (\text{wt in kg}) \times 0.85}{72 \times (\text{serum creatinine in mg/dL})}$$
   - AST: >2.5 x ULN
   - ALT: >2.5 x ULN
   - Total bilirubin >1.5 x ULN
   - Serum amylase >1.5 x ULN
   - Serum phosphorus <2.4 mg/dL
   - Urinalysis: Two abnormal dipsticks showing any of the following:
     - blood = 2+ or more (not due to menses);
     - protein = 1+ or more
     - leucocytes = 2+ or more
     - glucose= 1+ or more

4. Confirmed diagnosis of chronic hepatitis B infection (HbsAg positive)

5. If female, pregnant or planning a pregnancy within 4 months after enrolment or lactating.

6. Participation in another clinical study of an investigational product currently, within the 3 months prior to enrolment or expected participation during this study

5.2.7 Recruitment of Volunteers

Healthy adult male and female volunteers will be recruited from existing Open B study cohorts at the study research centres. Volunteers may continue to remain enrolled in Open B study while participating in this study. Study visits will be combined, when possible, and duplication of procedures, tests and data collection will be minimized. If other recruitment strategies are used, the sponsor must be informed in advance. During the recruitment process it is important to ensure full counselling and full informed consent.

6.0 STUDY VISITS

6.1 Screening Period

During Screening, research centre personnel will perform the following procedures:

- Ensure that each potential volunteer is educated about the study to ensure his/her trial comprehension. This education includes information about randomization, double-blinding, use of placebo, possible side-effects of the study drug, adherence, study duration, risks and benefits and informed consent.
  At the Kilifi centre, this education will be provided through an audio-computer assisted trial comprehension module, followed by a computer-based test. The test is designed to assess trial comprehension and produce a report of any misunderstandings and areas that need further attention and explanation. A printed report will be provided to the potential volunteer for discussion with the counsellor during the informed consent process. Face-to-face informed consent counselling and administration of the Assessment of Understanding must also occur.
- Provide and/or review the Informed Consent Document and answer any questions about the study prior to obtaining written informed consent.
- Administer Assessment of Understanding prior to obtaining written informed consent
- Obtain written informed consent prior to conducting any study procedures.

If the volunteer agrees to participate, research centre personnel will:

- Provide a screening questionnaire to the volunteer for completion
- Perform an HIV risk assessment.
- Perform a complete medical history (including concomitant medication and STI screening, see section 7.2.)
- Perform a general physical examination including height, weight, vital signs (pulse, respiratory rate, blood pressure and temperature), examination of skin, respiratory, cardiovascular and abdominal systems, an assessment of cervical and axillary lymph nodes.
- Conduct HIV pre-test counselling
- Conduct HIV risk reduction counselling and family planning counselling (for women only)

- Collect blood and specimens for all tests as indicated in the Schedule of Procedures (Appendix A).
- Perform a pregnancy test for all female volunteers.
- Conduct HIV post-test counselling

The volunteer must demonstrate that he or she is able to swallow a pill the size of FTC/TDF by swallowing a multivitamin of the same size in the presence of the study counsellor.

Screening laboratory test(s) may be repeated once at the discretion of the principal investigator or designee to investigate any isolated abnormalities.

If the screening visit occurs more than 42 days prior to the date of randomization, all screening procedures must be repeated. The complete medical history may be replaced by an interim medical history and the Volunteer Information Sheet should be reviewed.

If a volunteer has signed the informed consent form but does not meet the eligibility criteria, the records must be kept at the research centre and an aggregate report presented to IAVI.

6.2 Randomization Visit

Prior to the randomization, research centre personnel will:

- Answer any questions about the study
- Review interim medical history (including concomitant medications)
- Review screening safety laboratory data.
- Review the Informed Consent Document with the volunteer.
- Perform a symptom directed physical examination including vital signs (pulse, respiratory rate, blood pressure and temperature), and any further examination indicated by history or observation.
- Conduct HIV pre-test counselling.
- Collect blood and specimens for all tests as indicated in the Schedule of Procedures (Appendix A).
- Perform a pregnancy test for all female volunteers and obtain results prior to randomization.
- Conduct HIV risk reduction counselling and family planning counselling (for women)
- Conduct HIV post-test counselling

After the randomization, research centre personnel will:

- Collect Adverse Events and Serious Adverse Events
- Distribute investigational product with MEMS TrackCap
- Conduct adherence counselling
- Distribute SIM card or cell phone, if needed, for IVR or SMS
- Conduct IVR or SMS instruction

The volunteer will be assigned to a treatment arm (FTC/TDF or placebo) in a blinded fashion according to the instructions specified in the Study Operations Manual.

The investigational product will be distributed as specified in section 8.4 Distribution of Product.

6.3 Post-randomization Visits

The volunteer will be asked to return to the clinic on the days specified in Appendix A. Procedures will be conducted at these visits as specified in Appendix A.

During these visits, routine safety laboratory parameters from the previous visit will be reviewed. If a volunteer had an abnormal laboratory value, specified guidelines (Section 12.0) will be followed.

HIV pre-test counselling will be conducted if an HIV test is required (Appendix A) and post-test counselling will be provided if the results of a prior HIV test are given to the volunteer.

At the Week 4 visit only, a single-dose timed pharmacokinetic study will be conducted. In addition to the procedures listed above, the following procedures will be conducted:

- To reduce the variability in test results, volunteers will be asked to eat a standard meal.
- After eating, collection of blood specimen for plasma and intracellular drug levels
- Witnessed dosing of investigational product immediately after specimen collection
- Repeat collection of blood specimen approximately 2 hours after dosing
- Repeat collection of blood specimen approximately 24 hours after dosing

6.4 Unscheduled Visits/Contact

Unscheduled Visits/contacts are visits/contacts that are not described in the Schedule of Procedures (Appendix A). They may be performed at any time during the study. Unscheduled visits may occur:

- For administrative reasons, e.g., the volunteer may have questions for study staff or may need to re-schedule a follow-up visit.
- To obtain laboratory test results from a previous visit.
- For intercurrent illnesses
- For other reasons, as requested by the volunteer or research centre investigator.

All unscheduled visits will be documented in the volunteers' study records and on applicable source documents.

6.5 Final Visit/Early Termination Visit

Assessments and procedures will be performed according to the Schedule of Procedures (Appendix A).

Research centre personnel will:

- Review interim medical history and use of concomitant medications
- Perform a general physical examination including weight, vital signs (pulse, respiratory rate, blood pressure and temperature), examination of skin, respiratory, cardiovascular and abdominal systems, and external genital exam.
- Collect blood and specimens for tests as specified in the Schedule of Procedures (Appendix A).
- Perform a pregnancy test for all female volunteers.
- Conduct HIV pre-test counselling or provide post-test counselling if the results of a prior HIV test are being provided to the volunteer.
- Collect data on Adverse Events and Serious Adverse Events
- Upload MEMS data
- Collect self-report information on adherence, sexual risk behaviour, investigational product acceptability and medication sharing (i.e., follow back interview)
- Collect unused pills and MEMS TrackCap

6.6 After Final Visit/Early Termination Visit

6.6.1. Focus Group Discussion

Focus group discussions (FGD) will be held for volunteers after the week 16 visit. FGD will be held separately for men and women. A trained counsellor will facilitate discussion among 5-10 volunteers in a private place, using a written interview guide. Topics will include likes and dislikes of the study drug, study drug dosing schedule, adherence measures used during the study and recommendations to improve the feasibility of PrEP. The discussion will be tape recorded and transcribed, but volunteers will not be identified by name. Volunteers are free to decline participation in the focus group.

6.6.2. Individual Interviews

Volunteers who discontinue the study prematurely or who report less than 80% adherence may be asked to participate in an individual interview soon after their final visit. A trained counsellor will conduct a semi-structured interview in a private place, using a written interview guide. Topics will include likes and dislikes of the study drug, study drug dosing schedule, study procedures, and reasons for early discontinuation. The discussion will be tape recorded and transcribed, but volunteers will not be identified by name. Volunteers are free to decline participation in the individual interview.

6.7 HIV Follow-up Visits 1 and 2

Study volunteers who become HIV-infected during the study will discontinue investigational product and return for 2 additional study visits, HIV follow-up visits 1 and 2. Assessments and procedures will be performed according to the Schedule of Procedures (Appendix A).

The following procedures will be conducted at these visits:

- Review interim medical history and use of concomitant medications.
- Conduct a general physical exam (Follow-up visit 1) or conduct a symptom-directed physical exam (Follow-up visit 2)
- Review the routine safety laboratory parameters from the previous visit. If a volunteer has an abnormal laboratory value, follow the specified guidelines (Section 12.0)
- Collection of blood and specimens for all tests as indicated in the Schedule of Procedures (Appendix A).
- Conduct confirmatory HIV testing as needed to confirm HIV diagnosis
- Conduct HIV pre-test counselling if an HIV test is required or provide post-test counselling if the results of a prior HIV test are being provided to the volunteer.
- Conduct HIV risk reduction counselling and family planning counselling (for women)
- Provide referral for HIV care and treatment

7.0 STUDY PROCEDURES

7.1 Informed Consent Process

A sample Informed Consent Document consisting of a volunteer information sheet and an informed consent form is provided by the sponsor to the research centre. This document needs to be made research centre-specific and translated, submitted and approved by the IEC/ERB before it can be used at the research centre.

Volunteer Information Sheet
A qualified member of the research centre study staff can obtain informed consent only after reviewing the Volunteer Information Sheet with the volunteer.

Informed Consent Form
All volunteers will give their written informed consent to participate in the study on the basis of appropriate information and with adequate time to consider this information and ask questions.

The volunteer's consent to participate must be obtained by his/her signing or marking, and dating the informed consent form. The research centre personnel who are involved in conducting the informed consent discussions must also sign and date the informed consent form.

If the volunteer is functionally illiterate, the complete Informed Consent Document must be read to him/her in the language that the volunteer best understands and in the presence of an impartial witness not affiliated with the study, who will also sign and date the consent form as an impartial witness.

The signed/marked and dated Informed Consent Document must remain at the study research centre. A copy of the signed and dated Informed Consent

Document will be offered to the volunteer to take home. Those volunteers who do not wish to take a copy will be required to document that they declined to do so.

Family members, sexual partner(s) or spouse(s) will be offered education and counselling regarding a volunteer's participation in the study ONLY with the written consent of the participating volunteer.

7.2 Medical History and Physical Examination

At screening, a comprehensive medical history will be collected including history of sexually transmitted diseases and contraceptive practices.

A general physical examination includes the following: weight, height (at screening visit only), vital signs, and examination of skin, respiratory, cardio-vascular, central nervous and abdominal systems and external genital exam. Volunteers will be assessed for STI symptoms and provided or referred for appropriate management.

STI screening for males should include a history of STI symptoms, an ano-genital exam, blood test for syphilis, collection of urine specimen for gonorrhoea and chlamydia testing and, for males collection of a rectal swab for gonorrhoea and Chlamydia testing.

STI screening for females should include a history of STI symptoms, an external genital exam, blood test for syphilis, collection of urine specimen for gonorrhoea and Chlamydia testing, collection of a vaginal swab for trichomonas, candida vaginitis and bacterial vaginosis testing and collection of a rectal swab for gonorrhoea and Chlamydia testing.

At each study visit, an interim medical history and symptom directed physical examination will be performed. A symptom directed physical examination will include vital signs and any further examination indicated by history or observation.

7.3 HIV Risk Assessment, HIV Testing and HIV-test Counselling

Research centre personnel will assess volunteers for past and current risk of HIV infection. Additionally, research centre personnel will perform HIV pre-test counselling (prior to collecting blood for an HIV test) and HIV post-test counselling (when HIV test results are available) according to the Schedule of Procedures (Appendix A). For more information on HIV testing and HIV-test counselling, see Section 11.1.

7.4 HIV Risk Reduction Counselling

Research centre personnel will provide individual HIV risk reduction counselling, including free condoms, at every visit. The procedures for risk reduction counselling will be detailed in research centre-specific SOPs.

7.5 Family Planning Counselling

Research centre personnel will counsel female volunteers about the importance of preventing pregnancies and use of condoms as well as effective family planning methods. Condoms will be provided. Volunteers will be provided contraceptive counselling and provision at the research centre or referred to a family planning clinic if a contraceptive prescription is required.

The family planning counselling will be performed at time points according to the Schedule of Procedures (Appendix A).

7.6 Adherence Counselling

Adherence counselling will be performed at time points according to the Schedule of Procedures (Appendix A). At each follow-up visit, research centre personnel will assess volunteer adherence through a structured interview (follow back timeline) and pill count.

The structured interview will focus on self-reported adherence to the study medication, self-reported sexual activity, drug sharing, and challenges to adherence since the last visit (e.g., travel, illness, etc.). Research centre personnel will provide individualized adherence counselling at each follow-up visit, using the answers to the structured interview and pill count to guide the counselling messages.

Adherence counselling should include the following elements:

- Basic education about study medications such as mechanism of action, dose and dosing schedule, potential side effects, and storage
- Discussion of the volunteer's beliefs and attitudes about taking the investigational product
- Encouragement to talk to study personnel about medication questions or concerns
- Identifying and overcoming barriers to adherence, such as scheduling and organizational skills, reminder devices, social support, and disclosure.
- Discussion about reasons not to engage in pill-sharing and how to handle requests from friends and family to borrow doses

Complete information on procedures for adherence counselling is described in the Study Operations Manual.

7.7 Blood Collection

Up to 20 mL of blood will be collected at the Screening Visit and up to 70 mL of blood will be collected at each later visit, usually from the antecubital fossa, according to the Schedule of Procedures (Appendix A). Less blood may be collected if lab results can be obtained from specimens collected on the same date for the Open B study.

All specimens will be handled according to the procedures specified in the Study Operations Manual.

In the event of an abnormal laboratory value, volunteers may be asked to have an additional sample collected at the discretion of the principal investigator or designee.

7.8 Urine Collection

Up to 50 mL of urine will be collected at the specified timepoints in the Schedule of Procedures (Appendix A). All specimens will be handled according to the procedures specified in the Study Operations Manual. In the event of an abnormal laboratory value, volunteers may be asked to have an additional sample collected at the discretion of the principal investigator or designee.

7.9 Vaginal and Rectal Swab Collection

Vaginal and rectal swabs will be collected by the volunteers, after instruction, at the screening visit (see Section 7.2.). All specimens will be handled according to the procedures specified in the Study Operations Manual. In the event of an abnormal laboratory value, volunteers may be asked to have an additional sample collected at the discretion of the principal investigator or designee.

7.10 Hair Collection

Hair samples will be collected from volunteers at the specified time points in the Schedule of Procedures (Appendix A). Details on hair collection are provided in the Study Operations Manual.

7.11 Reimbursement

Volunteers will be reimbursed for their time, effort and for costs to cover their travel expenses to the study research centre and any inconvenience due to study participation. Reimbursement will be made after the completion of each study visit. Research centre specific reimbursement amounts will be documented in the research centre-specific Volunteer Information Sheet approved by the Ethics Committee.

7.12 Randomization and Blinding

Volunteers will be identified by a unique volunteer identification number.

The randomization schedule(s) will be prepared by the statisticians at the Data Coordinating Center (DCC) prior to the start of the study. Volunteers will be assigned a specific allocation number. An unblinding list will be provided to the research centre by the DCC for use in case of medical emergency as described below.

This study is double-blinded. Research centre personnel (investigator and clinical personnel monitoring the safety and laboratory assay results) and volunteers will be blinded with respect to the allocation of investigational product (placebo or FTC/TDF) but dosing regimen, daily or intermittent, will not be blinded.

Volunteers will be informed about their group assignment once the database is locked. If a study volunteer is unblinded and becomes aware of treatment group assignment, further administration of the investigational product (FTC/TDF or

placebo) will be discontinued. The study volunteer will be followed up until the end of the study.

7.13 Unblinding Procedure for Individual Volunteers

Unblinding of an individual volunteer may be indicated in the event of a medical emergency when the clinical management/medical treatment of the volunteer would be altered by knowledge of the group assignment.

The unblinded information should be restricted to only a small group of individuals involved in clinical management/medical treatment of the volunteer (e.g., treating physician) and the blind should be maintained for those responsible for the study assessments.

The reasons for unblinding should be documented and the IAVI Medical Monitor and the Data Coordinating Centre should be notified. The procedures and contact numbers for unblinding are outlined in the Study Operations Manual.

8.0 INVESTIGATIONAL PRODUCT

8.1 Description

This study evaluates the use of FTC/TDF (FTC 200mg /tenofovir disoproxil fumarate 300mgFTC co formulation) or placebo. The placebo will appear identical to FTC/TDF.

FTC/TDF tablets are fixed dose combination tablets containing 200 mg FTC and 300 mg TDF disoproxil fumarate as active ingredients.

FTC is a nucleoside reverse transcriptase inhibitor (NRTI) approved as once-a-day capsule (200 mg) in combination with other antiretroviral agents, for the treatment of HIV-1 infection. FTC is a synthetic analogue of the naturally occurring pyrimidine nuceloside, 2'-deoxy cytidine. Intracellularly, FTC is phosphorylated by cellular enzymes to form FTC triphosphate, the active metabolite. Tenofovir disoproxil fumarate (Viread, also known as TDF) the oral prodrug of tenofovir, is a nucleotide reverse transcriptase inhibitor (NRTI) approved as a once-a-day tablet (300 mg), in combination with other antiretroviral agents, for the treatment of HIV-1 infection. After absorption, tenofovir DF is rapidly converted to tenofovir, which is metabolized intracellularly to the active metabolite, tenofovir diphosphate (PMPApp).

FTC/TDF tablets are for oral adminstration. Each blue, film-coated, capsule-shaped tablet is labelled with 'GILEAD' on one side. Each tablet contains 200 mg of FTC and 300 mg of TDF (which is equivalent to 245 mg of tenofovir disoproxil), as active ingredients. The tablets also include the following inactive ingredients: croscarmellose sodium, lactose monohydrate, magnesium stearate, microcrystalline cellulose, and pregelatinizaed starch (gluten free). The tablets are coated with Opadry II Blue Y-30-10701, which contains FD&C Blue #3 aluminium lake, hypromellose, lactose monohydrate, titanium dioxide, and triacetin.

The placebo tables match the FTC/TDF tablets in appearance. Each tablet contains the following inactive ingredients: microcrystalline cellulose, croscarmellose sodium, pregelatinized starch, lactose monohydrate, and magnesium stearate. The tablets are film-coated with FD&C Blue #2 aluminum lake, hypromellose, lactose monohydrate, titanium dioxide, and triacetin.

See the Investigators Brochure for details of the chemical structure and properties of both drugs.

8.2 Shipment and Storage

Authorization to ship the investigational product to the research centre will be provided in writing by the sponsor (IAVI), upon confirmation that all required critical documents for shipment authorization are completed. The investigational product will be shipped to the research centres at 25°C and stored there at 25°C. Temperature excursions will be allowed between 15-30°C.

8.3 Dispensing and Handling

The investigational product will be dispensed as specified in the Study Operations Manual. Designated research centre personnel will ensure that the allocation number matches the allocation number assigned to the volunteer. The investigational product will be used as supplied by the manufacturers with no further preparation.

Daily dosing: 1 tablet orally daily

Intermittent dosing: 1 tablet orally Monday and Friday and again within 2 hours after sexual intercourse (except on Monday and Friday), not to exceed 1 dose per day.

Study volunteers will be instructed to store investigational product below 40°C.

8.4 Distribution

Investigational product will be distributed according to the Schedule of Procedures (Appendix A).

Investigational product will be distributed approximately every 4 weeks in a pill bottle fitted with a MEMS TrackCap. Volunteers will receive sufficient pills to allow for the daily or intermittent regime i.e., twice weekly doses, post-coital doses up to one total dose per day (except on the days of the weekly doses, Monday and Friday) for the 4 weeks between study visits. Unused pills will be accounted and collected at the next visit prior to dispensation of the next month's supply.

Further information on the distribution of the investigational product is supplied in the Study Operations Manual.

8.5 Accountability and Disposal

At the research centre, investigational product will be stored in a locked area with limited access. During the study, the investigational product accountability form, the dispensing log and the log of returned drug will be kept and monitored.

All returned drug will be returned to the investigational product dispenser or pharmacy at the end of each visit. At the end of the study, the unused drug will be destroyed after the approval of the sponsor and according to the research centre's SOP for destruction of investigational product.

9.0 ASSESSMENTS

9.1 Safety and Tolerability Assessments

9.1.1 Clinical adverse events

Occurrence of clinical adverse events (including Serious Adverse Events) will be collected following an open question to volunteers on the time points according to the Schedule of Procedures (Appendix A). The adverse events will be graded using the Appendix B, Adverse Event Grading Toxicity Table as a guideline. For more information regarding adverse events refer to Section 10.0, Adverse Events.

9.1.2 Concomitant Medications

During the study, information regarding concomitant medications and reasons for their use will be solicited from the study volunteers at each visit and recorded. Concomitant receipt of investigational products is prohibited during the study.

9.1.3 Routine laboratory parameters

Table 3 shows the laboratory parameters that will be measured routinely. These tests will include haematology and clinical chemistry. The samples for these tests will be collected at the time points indicated in the Schedule of Procedures (Appendix A).

Table 3
Laboratory Parameters

Laboratory Parameter	Test
Haematology	Full blood count (haemoglobin, leucocytes, platelets)<br>Differential count (absolute neutrophil count, absolute lymphocyte count)
Clinical Chemistry	Creatinine, creatinine clearance, serum phosphate,<br>Liver function tests: aspartate transferase (AST), alanine transferase (ALT), total bilirubin

9.1.4 Specific screening tests:

Volunteers will be screened to exclude or detect the following diseases:

- Pancreatitis: Serum amylase
- Hepatitis B: positive for hepatitis B surface antigen (HbsAg)
- Syphilis: confirmed diagnosis of active syphilis
- Chlamydia: urine nucleic acid probe for chlamydia
- Gonorrhoea: urine nucleic acid probe for gonorrhoea
- In male volunteers: Chlamydia and gonorrhoea nucleic acid probe on rectal swab
- In females, vaginal swab for trichomonas, candidiasis and bacterial vaginosis

9.1.5 Specific tests in the event of HIV infection

Volunteers who are found to be HIV-infected during the study will be tested for:

- CD4+ T cell count
- HIV viral load
- P24 antigen and other assays for early seroconversion and virus detection
- HIV genotyping for drug resistance mutations
- Viral tropism assays
- HIV sequencing

9.2 Immunology Assessments

9.2.1 Antibody Responses

Serum antibodies against HIV antigens will be measured by ELISA at the time points indicated on the Schedule of Procedures (Appendix A). Sponges/Swabs will be used to collect mucosal secretions (vaginal and/or anal) for antibody secretion. SOPs for collection, processing and storage of these specimens will be developed in collaboration with IAVI collaborators and investigators. Immunoglobins such as IgA will be extracted from thawed secretions and assayed by ELISA. If IgA and/or IgG responses are detected in mucosal secretions and/or serum, these specimens may be further evaluated in functional antibody assays such as HIV neutralization assays at suitable testing facilities.

9.2.2 Cellular Responses

Blood for immunogenicity assays will be taken at time points indicated in the Schedule of Procedures. PBMC will be processed, cryopreserved at 10 million PBMC/vial and stored in accordance with established IAVI SOPs as outlined in the Laboratory Analytical Plan. As the immune response in individuals on PrEP has not previously been evaluated systematically, in this protocol some exploratory evaluations of immune responses will be undertaken. As new information from pre-clinical studies and human studies becomes available on what immune responses develop and/or which assays might detect such immune responses during PrEP treatment, this knowledge will be applied to the IAVI studies. Cell mediated immune responses will be evaluated using PBMC and population-based HIV overlapping peptide pools or optimized HIV peptide sets, as described in the Laboratory Analytical Plan. The first tier assay may include ELISPOT (standard and more sensitive cultured assay format) for monitoring the number of circulating T-cells that can be stimulated to produce cytokines such as IFN-γ and Il-2. The ELISPOT assay will be conducted at the research centres where feasible. If ELISPOT responses are detected, further characterization of phenotype and functional properties of responding T-cells will be performed using multi-parameter flow cytometry and functional assays, including a viral inhibition assay (VIA), which measures the composite CD8+ T lymphocyte antiviral activity. Proliferative capacity and activation status of

circulating and mucosal mononuclear cells will also be evaluated using flow cytometry techniques. An algorithm may be applied to prioritize assays based on available specimens from each of the FTC/TDF daily, FTC/TDF intermittent, placebo daily, or placebo intermittent treatment groups.

Where possible the immunology studies will be conducted at the research centres. However the multi-parameter flow cytometry requires sophisticated equipment which is currently only available at the Core Lab and the Uganda Virus Research Institute (UVRI). The VIA assay requires containment level three tissue culture facilities. Attempts will be made to transfer assays and reagents to participating laboratories. Further studies may be carried out using a) individual peptides in a matrix or other format to determine the specific epitopes recognized and b) peptides from different HIV-subtypes. Selected T-cell responses may be further characterized for HLA restriction and additional markers on the responding cells, such as markers for activation or homing to mucosal tissues.

9.2.3 PBMC, Serum and Plasma Storage

Further details regarding the type of collection tube, the volume of blood, and specimen handling procedures can be found in the Laboratory Analytical Plan, and in the research centre and laboratory SOPs.

Plasma and serum stored samples will be archived in two locations for potential future testing and quality control, with one portion at the research centre's laboratory and the other portion at a central, quality controlled laboratory. Samples will be catalogued and stored in conditions that ensure appropriate power supply back-up and accurate sample retrieval. PBMCs will be used for assessments of immune functions, HLA mapping, genetic analyses, virus isolation and other HIV and immune system-related tests. Genetic analyses will be limited to those factors that may influence HIV acquisition and disease in population based studies or metabolism of the study drug. PBMCs will be separated at local laboratories using sponsor-approved SOPs and stored at one central laboratory (IAVI Core Laboratory) under Good Clinical Laboratory Practices (GCLP) conditions.

Ethics Review Committee notification or approval will be obtained for all sample requests and testing performed on archived samples that are not described in the list above. Samples may be stored indefinitely at selected outside laboratories under the current Ethics Committee (EC) approval. All specimens will be shipped in accordance with International Air Transport Association specimen shipping regulations for infectious materials.

Stored samples will be shipped routinely from each research centre to the IAVI Core Laboratory or other designated contract laboratories or collaborating institutions.

The immunological testing will be performed at the research centres where feasible and at the IAVI Core Laboratory in accordance with IAVI standard operating procedures and standard reagents.

9.3 Acceptability Assessments

Acceptability of the investigational product and study regimen will be assessed through follow-back interview and focus group/individual interview at the time point indicated in Appendix A.
Reported willingness to use the study regimen, if shown to be effective will be the main measure of acceptability.

The acceptability of certain study procedures will also be assessed, including IVR or SMS to record sexual activity data, MEMS TrackCaps and hair sampling to measure adherence and the use of ACASI to collect study information.

9.4. Adherence Assessments

Adherence to and dosing of investigational product will be assessed through MEMS data, follow-back interview and plasma/intracellular drug levels at the time points indicated in Appendix A.

MEMS electronic medication-monitoring pill bottle caps. Daily medication adherence data will be collected using the AARDEX Medication Event Monitoring System (slightly larger than a standard pharmacy-sized bottle cap with an electronic chip that clocks bottle openings in a 24-hour period). This measure provides complete information on patterns of adherence, including treatment interruptions. Each volunteer will be assigned a MEMS TrackCap to be used on his or her study drug pill bottles. MEMS data for all volunteers regardless of assignment group will be uploaded from the device at monthly visits as indicated in Appendix A.

Self-reported adherence. At timepoints indicated in the Schedule of Procedures (Appendix A) self-reported adherence to PrEP will be collected using a follow-back interview which is a structured self-report of number of doses missed. A 30 day self-report was shown to be closely associated with MEMS adherence in Uganda [77,110]. In Kilifi, the interview will be administered via ACASI.

Plasma drug levels. Plasma from all volunteers will be stored at multiple visits as indicated in Appendix A for retrospective assessment of drug plasma levels in those volunteers assigned to FTC/TDF. The plasma half-life of tenofovir after a single oral dose is approximately 17 hours, but drug remains detectable (>10ng/mL) out to 48 hours [43]. At the end of the study after treatment assignment has been unblinded, samples will be selected from up to 4 visits when the volunteer's MEMS data indicate dosing with FTC/TDF within 48 hours of the visit and tested for plasma drug levels.

Two additional adherence measurements will be explored. Intracellular drug levels will be measured in volunteers assigned to FTC/TDF. PBMCs will be collected from all volunteers at the time points indicated in Appendix A. After

treatment assignments are unblinded, samples from volunteers assigned to FTC/TDF will be tested for intracellular drug levels. The relationship between adherence pattern and intracellular drug level will be explored to see if these drug levels could be used as a proxy for adherence in future studies. Similarly, hair samples will be collected from all volunteers, tested for drug levels in volunteers assigned to FTC/TDF and correlated with adherence patterns. Hair collection will take place at the time points indicated in Appendix A.

9.5. Behavioural Assessments

Behavioural Assessments will be assessed through IVR or SMS and follow-back interview at the time points indicated in Appendix A.

Interactive voice response or SMS measurement of daily sexual behavior. Based on methods of Schroder[140], volunteers will be provided with a SIM card or a cell phone for the daily measurement of sexual behaviour using either IVR or SMS. Participants will be contacted daily to digitally answer questions similar to the following questions: 1) Did you have anal or vaginal sex with your main partner yesterday? 2) (if yes) Did you use a condom? 3) Did you have anal or vaginal sex with any other partner yesterday? 4) (if yes) Did you use a condom? Data will be recorded via entering the numerical response via cell phone keypad (press 1 for yes, 2 for no). Total interview time will be under 60 seconds. To encourage daily reporting, individuals will be reimbursed with 3 minutes of air time for each call. Individuals not responding will be contacted again by cell phone to elicit a response. Breach in confidentiality via unauthorized use will be prevented through automatic confirmation of the assigned cell phone number and asking the volunteer to enter a password to confirm his/her identity prior to the questions. All volunteers will have an instructive session on the use of the system.

Follow-back interview. The timeline follow-back interview is a calendar-aided face-to-face interviewing method during which participants are asked to retrospectively report daily sexual behaviour. This approach has shown strong retest reliability [141, 163]. Interviewers will be trained according to the method developed by Sobell and Sobell [164]. Interviewer gender will be matched to the gender of the volunteer. Volunteers will be given a customized calendar. Volunteers will be asked to mark personal ‘‘anchor’’ days or ‘‘holidays’’ on the calendar such as a market days, church attendance, national holidays, or weddings [164]. Volunteers will mark each day they had anal and vaginal intercourse, the number of sexual partners, and code whether a condom was used. Additional risk data will be collected, such as substance use before or during sex. In Kilifi, this method will be adapted for ACASI.

9.6 Medication Sharing Assessments

Potential for medication sharing will be assessed through the follow-back interview at the time points indicated in Appendix A.
It will also be assessed by testing intracellular drug levels in placebo recipients after the study is unblinded. Hair samples from placebo recipients may also be tested for drug levels but only for exploratory data.

9.7. Other Assessments

9.7.1 Drug Levels

Plasma samples to determine drug levels and PBMC samples to determine intracellular drug levels and hair samples to determine drug levels will be collected a the time points indicated in the Schedule of Procedures (Appendix A)

9.7.2 HLA Typing and other genetic analysis

Samples for HLA typing and other genetic tests related to HIV susceptibility will be collected at the time point indicated in the Schedule of Procedures (Appendix A).

9.7.3 HIV Test

Samples will be tested at the time points indicated in the Schedule of Procedures (Appendix A). Further information is specified in Section 11.1 HIV Testing.

9.7.4 Pregnancy Test

A urine pregnancy test for all female volunteers will be performed by measurement of Human Chorionic Gonadotrophin (βhCG) at the time points indicated in the Schedule of Procedures (Appendix A).

The results of the pregnancy test must be negative prior to randomization and prior to subsequent distribution of investigational product.

10.0 ADVERSE EVENTS

10.1 Definition

An adverse event (AE) is any untoward medical occurrence in a volunteer administered investigational product (including Placebo); it does not necessarily have a causal relationship with the investigational product. An AE can therefore be any unfavourable or unintended sign (including an abnormal laboratory finding), symptom, or disease, temporally associated with the use of the investigational product whether or not related to the investigational product.

10.2 Assessment of Severity of Adverse Events

Assessment of severity of all AEs is ultimately the responsibility of the principal investigator.

The following general criteria should be used in assessing adverse events as mild, moderate, severe or potentially life-threatening:

- Mild: Mild discomfort; Minimal or no limitation of daily activities; Medical intervention not required;

- Moderate: Moderate discomfort; Some limitation of daily activities but able to work part-time or full-time with some assistance; May require minimal or no medical intervention;
- Severe: Severe discomfort; Marked limitation of daily activities, unable to work; Requires medical intervention;
- Very severe (listed as “potentially life threatening” in Appendix B): Extreme limitation in activity, significant assistance required, significant medical intervention therapy required, hospitalization required

Guidelines for assessing the severity of specific adverse events and laboratory abnormalities are listed in Appendix B, Adverse Event Severity Assessment Table.

10.3 Relationship to Investigational Product

The relationship of an AE or serious adverse event (SAE) is assessed and determined by the Principal Investigator or designee. All medically indicated and available diagnostic methods (e.g., lab, blood smear, culture, X-ray, etc.) should be used to assess the nature and cause of the AE/SAE. Best clinical and scientific judgment should be used to assess relationship of AE/SAEs to the investigational product and/or other cause.

The following should be considered for the assessment of relationship of adverse events to the investigational product:

- Presence/absence of a clear temporal (time) sequence between administration of the investigational product and the onset of AE/SAE

- Presence/absence of another cause that could more likely explain the AE/SAE (concurrent disease, concomitant medication, environmental or toxic factors, etc.)

- Whether or not the AE/SAE follows a known response pattern associated with the investigational product

The relationship assessment should be reported as one of the following:
Not Related: clearly explained by another cause (concurrent disease, concomitant medication, environmental or toxic factors, etc.).

Unlikely: more likely explained by another cause (concurrent disease, concomitant medication, environmental or toxic factors, etc.).

Possibly: equally likely explained by another cause but the possibility of the investigational product relationship cannot be ruled out (e.g., reasonably well temporally related and/or follows a known investigational product response pattern but equally well explained by another cause).

Probably: more likely explained by the investigational product (e.g., reasonably well temporally related and/or follows a known investigational product response pattern and less likely explained by another cause).

Definitely: clearly related and most likely explained by the investigational product

For the purpose of expedited safety reporting, all possibly, probably or definitely related SAEs are considered investigational product related SAEs.

10.4 Serious Adverse Events

An adverse event is reported as a "Serious Adverse Event" if it meets any the following criteria (as per ICH GCP Guidelines):

- Results in death
- Is life threatening
- Results in persistent or significant disability/incapacity.
- Requires in-patient hospitalization or prolongs existing hospitalization.
- Is a congenital anomaly/birth defect or spontaneous abortion.
- Any other important medical condition that requires medical or surgical intervention to prevent permanent impairment of a body function or structure.

SAEs must be reported to IAVI within 24 hours of the research centre becoming aware of the event. All SAEs must be reported using the designated SAE Report Form and sent to the sponsor according to SAE Reporting Guidelines (see Study Operations Manual).

To discuss investigational product related SAEs or any urgent medical questions related to the SAE, the research centre investigator should contact one of the IAVI medical monitors directly (see the Contact List).

The IAVI SAE Report Form should be completed with all the available information at the time of reporting. The minimum data required in reporting an SAE are the volunteer identification number, date of birth, gender, event description (in as much detail as is known at the time), onset date of event (if available), reason event is classified as Serious, reporting source (name of principal investigator or designee), relationship assessment to the investigational product by the investigator.

The Principal Investigator or designee is required to write a detailed written report with follow up until resolution or until it is judged by the principal investigator or designee to have stabilized.

The Principal Investigator or designee must notify the local IRB/IEC of all SAEs as appropriate. In case of investigational product related SAEs, the sponsor and/or the investigational product provider will notify local regulatory authorities, Safety Review Board and other study research centres where the same investigational product is being tested.

More details on SAE definitions and reporting requirements are provided in the SAE Reporting Guidelines (see Study Operations Manual).

10.5 Clinical Management of Adverse Events

Adverse events (AEs) will be managed by the clinical study team who will assess and treat the volunteer as appropriate, including referral. If any treatment/medical care is required as a result of the harm caused by the investigational product or study procedures, this care will be provided free of charge.

If a volunteer has an adverse event and/or abnormal laboratory value that is known while the volunteer is taking the investigational product, the specifications of Section 12.0 will be followed.

Volunteers will be followed until the adverse event resolves or stabilizes or up to the end of the study, whichever comes last. If at the end of the study, an adverse event (including clinically significant lab abnormality) that is considered possibly, probably or definitely related to the investigational product is unresolved, follow-up will continue until resolution if possible and the volunteer will be referred.

10.6 Pregnancy

Although not considered an adverse event, if a female volunteer becomes pregnant during the study, it is the responsibility of the Principal Investigator or designee to report the pregnancy promptly to IAVI using the designated forms. However, serious complications of pregnancy that meet SAE criteria specified in the Section 10.4 of this Protocol (e.g., eclampsia, spontaneous abortion, etc.) should be reported as SAEs.

If a female volunteer becomes pregnant during the study, the administration of the investigational product will be discontinued and the volunteer followed until the end of pregnancy. Approximately 2–4 weeks after delivery, the baby will be examined by a physician to assess its health status and the results will be reported to IAVI.

10.7 Intercurrent HIV Infection

If a volunteer is found to be HIV-infected through exposure in the community, the administration of the investigational product must be discontinued and the volunteer followed according to procedures described in Section 12.2.

Intercurrent HIV infection in study volunteers, although not considered an SAE must be reported promptly to IAVI using the designated forms. However, serious medical conditions associated with the HIV infection that meet SAE criteria specified in the Section 10.4 of this Protocol (e.g., sepsis, PCP pneumonia, etc.) should be reported as SAEs using SAE Report Form.

11.0 MANAGEMENT OF HIV ISSUES DURING AND FOLLOWING STUDY

11.1 HIV Testing

Volunteers will be tested for HIV-1 and HIV-2 antibodies as indicated in the Schedule of Procedures (Appendix A) or as needed, if medical or social circumstances arise. Frequent HIV testing is an important component of this protocol, so investigational product will be stopped immediately after HIV infection is detected. This process may help avoid the risk of developing drug resistant viral mutations.

All volunteers will receive HIV prevention counselling and pre-HIV-test and post-HIV-test counselling as specified in Section 11.2.

11.2 HIV infection

Volunteers who are found to be HIV infected at screening and volunteers who acquire HIV infection during the study will be provided the following:

11.2.1 Counselling

The volunteer will be counselled by the study counsellors. The counselling process will assist the volunteer with the following issues:

- Psychological and social implications of HIV infection
- Whom to inform and what to say
- Implications for sexual partners
- Implications for child-bearing
- Avoidance of transmission to others in future

11.2.2 Referral for Support and/or Care

Volunteers will be referred to a patient support centre or institution of his/her choice for a full discussion of the clinical aspects of HIV infection. Referral will be made to a designated physician or centre for discussion of options of treatment of HIV-infection.

For those individuals who become HIV infected after enrolment in the study, antiretroviral therapy will be provided when clinically indicated according to accepted treatment guidelines. If not available through another program, antiretroviral therapy will be provided at no charge for up to 5 years after treatment is initiated.

HIV-infected pregnant women will be referred for prenatal care and to a program for the Prevention of Mother to Child Transmission (PMTCT). The pregnant volunteer will be followed according to timeline as specified in Section 10.6.

11.2.3 Follow-up of HIV-Infected Volunteers

If a volunteer becomes HIV-infected during the study, they will be followed for an additional two visits as detailed in Appendix A to collect blood to characterize the infection, including CD4+ T cell count, HIV viral

load, p24 antigen and other assays for early seroconversion and virus detection, Western Blot, HIV genotyping for drug-resistance mutations, viral tropism assays, HIV sequencing and other testing to characterize the infection. Then the volunteer will be offered enrollment into a separate long-term follow-up protocol to follow their HIV infection.

12.0 DISCONTINUATION OF ADMINISTRATION OF INVESTIGATIONAL PRODUCT AND/OR WITHDRAWAL FROM STUDY

12.1 Discontinuation of administration of investigational product

Any volunteer discontinuing from investigational product or being considered for discontinuation of investigational product will be discussed with the sponsor. Volunteers will be discontinued from investigational product for any of the following reasons:

1. Pregnancy
2. Intercurrent HIV infection
3. A disease or condition or an adverse event that may develop, regardless of relationship to the investigational product, if the principal investigator or designee is of the opinion that further investigational product is not in the best interest of the volunteer.
4. Use of antiretroviral medications or other medications that may interact with investigational product
5. Volunteer request to discontinue further participation.
6. Participating in another clinical study of an investigational product
7. Creatinine clearance (CrCl) ≤50 mL/min, as calculated by Cockcroft-Gault formula
8. Any of the other following abnormal laboratory parameters that occur after randomization:

For all laboratory values except creatinine elevations:

Grade 1 or 2

Continue study medication at the discretion of the study physician.

Grade 3 or 4

May continue study medication for up to 3 days while values are confirmed

Confirmed Grade 3

- If unrelated or unlikely related to study drug by above definitions, may continue dosing
- If causal relationship to study drug possible by above definitions (e.g., possibly, probably, or definitely related), withhold drug until toxicity returns to < grade 3 then restart study drug
- If toxicity ≥ grade 3 recurs, permanently discontinue dosing follow as frequently as clinically indicated (up to study closure) until toxicity resolves

Confirmed Grade 4

Permanently discontinue study drug

Follow as frequently as clinically indicated (up to study closure) until toxicity resolves

Serum Creatinine Elevation

All Grades

Continue study medication for up to 3 days while values are confirmed

Confirmed Grade 1

Continue study medication at the discretion of the study physician
Monitor weekly until return to within 0.3 mg/dL of the baseline value

Confirmed Grade 2-4

Permanently discontinue study drug
Monitor as clinically indicated (generally at least monthly up to 3 months) until return to within 0.3 mg/dL of the baseline value

12.2. Follow Up After Discontinuation of Further Administration of Investigational Product

Volunteers, in whom further administration of investigational product are discontinued due to adverse events, will be followed until the adverse event resolves or stabilizes or up to the end of the study, whichever comes last. Volunteers who are discontinued for any reason will not be replaced.

HIV infected individuals who have received investigational product will be followed for an additional two visits after discontinuing investigational product to assess for the development of drug resistance mutations. If drug resistance is detected and determined to be related to study participation, further follow-up will be determined by the Safety Review Board.

Follow-up of pregnant volunteers will be done as specified in Section 10.6.

12.3 Withdrawal from the Study (Early Termination)

Volunteers may be withdrawn from the study permanently for the following reasons:

1. Volunteers may withdraw from the study at any time if they wish, for any reason.
2. The principal investigator or designee has reason to believe that the volunteer is not complying with the protocol.
3. If the sponsor decides to terminate or suspend the study.

12.4. Follow-up Withdrawal from the Study (Early Termination)

If the volunteer withdraws from the study, all termination visit procedures will be performed according to the Schedule of Procedures (Appendix A) when possible. Every effort will be made to determine and document the reason for withdrawal from the study.

13.0 DATA HANDLING

13.1 Data Collection and Record Keeping at the Study Research centre

Data Collection: All study data will be collected by the clinical study staff using designated source documents and entered onto the appropriate case report forms (CRFs). CRFs will be provided by IAVI and should be handled in accordance with the instructions from IAVI. All study data must be verifiable to the source documentation. A file will be held for each volunteer at the clinic(s) containing all the source documents. Source documentation will be available for review to ensure that the collected data are consistent with the CRFs.

All CRFs and laboratory reports will be reviewed by the clinical team, who will ensure that they are accurate and complete.

Source documents and other supporting documents will be kept in a secure location and remain separate from volunteer identification information (name, address, etc.) to ensure confidentiality.

Standard GCP practices will be followed to ensure accurate, reliable and consistent data collection.

Source documents include but are not limited to:

- Signed Informed Consent Documents
- Dates of visits
- Documentation of any existing conditions or past conditions relevant to eligibility
- Reported laboratory results
- All adverse events
- Concomitant medications
- Questionnaires

13.2 Data Entry at the Study Research Centre

The data collected at the research centre will be entered onto the CRFs by the research centre personnel. To provide for real time assessment of safety, data should be entered as soon as reasonably feasible (e.g., within one week) of a visit.

13.3 Data Analysis

The data analysis plan will be developed and agreed upon by the sponsor, the principal investigators and Study Chair prior to unblinding of the study. The statistician at the Data Coordinating Centre, in collaboration with the Principal Investigators and the Sponsor, will create tables according to this data analysis plan.

The DCC will conduct the data analysis and will provide interim and final study reports for the sponsor, principal investigator, the SRB and the regulatory authorities as appropriate. Senior statisticians at the collaborating research centre may participate in data analysis.

14.0 STATISTICAL CONSIDERATIONS

14.1 Sample Size

Approximately 72 volunteers (24 daily FTC/TDF, 24 intermittent FTC/TDF, and 24 combined placebo recipients) who meet all eligibility criteria will be enrolled in the study.

14.2 Statistical Power and Analysis

Prior to the analysis of safety, adherence, risk behaviour, and HIV-specific immune response data, integrity of study conduct and study group comparability will be examined to assure the validity of study results. All analyses will be based on the modified intention-to-treat cohort defined as all randomized individuals who are HIV-1 negative at the time of randomization. Volunteers will be analyzed according to the group to which they were randomized.

14.2.1 Safety and tolerability

Adverse events will be classified according to the MedDRA body system and preferred terms. The number and percentage of subjects experiencing a given AE will be tabulated by treatment group, grade (severity/relationship), and time since randomization.

Safety and tolerability will be assessed by examining the overall rates of adverse events and serious adverse events that are possibly, probably, or definitely associated with FTC/TDF. With 24 volunteers assigned to daily or intermittent FTC/TDF, observing no drug-related serious toxicity would result in an exact, two-sided, 95% confidence interval of (0.0000, 0.1425). If one drug-related SAE is observed, the exact, two-sided, 95% confidence interval would be (0.0011, 0.2112).

This study will have limited power to rule out all but very large relative increases in the risk of an adverse event due to FTC/TDF. For example, if 5% of the volunteers in the combined control group are expected to experience a given AE, this study has only 55% power to rule out a 10-fold increase (i.e., 5% vs. 50%) if there is no real increase. It has 63% power to rule-out a 15-fold increase (5% vs. 75%). For known FTC/TDF toxicities in HIV-infected persons, the observed AE rate among FTC/TDF recipients will be compared to historical data.

All clinical and routine laboratory data will be included in the safety analysis. Rate of study discontinuation and reasons for discontinuation will be determined by treatment group.

14.2.2 Acceptability

At the Week 12 and Week 16 visit, volunteers will be asked about their willingness to use the drug regimen in the future if it is proven to be effective. The most recent response will be used for analysis. Volunteers who drop-out of the study prior to Week 12 will be assumed to be not

willing to use the drug regimen. Volunteers who become HIV infected will be excluded. The proportion of volunteers in the daily FTC/TDF group who express their willingness will be calculated along with the exact, two-sided, 95% confidence interval for the true proportion. Likewise, the proportion of volunteers in the intermittent truvada group will be calculated.

14.2.3 Days per week on which study treatment is used

For each study month, the average number of days per week on which study treatment was used will be calculated. The proportion of volunteers using study treatment 5 or more days a week will be calculated. The frequency of study treatment use on each day of the week will be calculated.

14.2.4 Adherence

The mean, median and range of adherence to PrEP will be determined using MEMS data for volunteers. Previous work has shown that the accuracy of MEMS asssessment can be improved with a 3-day structured self-report of the number of extra cap openings and the number of times multiple doses are taken out of the bottle [119]. The *adjusted MEMS adherence* is calculated by the interview adjusted number of bottle openings/number of doses prescribed over the prior three days. The *unadjusted MEMS* measure is the number of electronically recorded bottle openings divided by the number of expected doses based on IVR sexual activity data, since the last interview[48]. MEMS adherence will be classified by the schema proposed by Vrijens where adherence is organized in terms of persistence and execution [161,162]. Persistence is time to treatment discontinuation. Treatment discontinuation will be defined as the first day of 30 consecutive days without a MEMS cap event. Treatment execution is defined as the number of MEMS caps events divided by the number of expected doses prior to treatment discontinuation. We also will use MEMS to characterize treatment interruptions and whether patterns of adherence are temporally related to sexual behavior. These approaches have been successfully piloted in Uganda [77,94].

For volunteers assigned to intermittent regimens, a combination of MEMS and IVR/SMS data will be used. Each day will be classified as an adherent or nonadherent day. Adherent days will be defined as: any day on which sexual activity was reported and a MEMS event occurred, any Monday or Friday on which a MEMS event occurred, and any other day of the week on which neither sexual activity nor a MEMS event occurred. Adherence will be calculated as the number of adherent days since the last visit divided by the number of days since the last visit.

We will examine the degree of correspondence between MEMS and 30 day recall data using a linear model, taking repeated measures into account, to compare predicted adherence from MEMS and IVR/SMS with 30 day recall data. Similarly adherence from MEMS will be compared with plasma and intracellular drug levels for correspondence. We will determine if PrEP adherence declines over time by comparing median

adherence each month using a linear model, testing for a non-zero slope and proportion >90% adherent using logistic regression. Finally we will describe time to PrEP discontinuation (or non-persistence), defined by >30 days without a MEMS event, with Kaplan-Meier methods. Volunteers who discontinued study treatment due to intolerance, toxicity, or pregnancy will be censored at the time of treatment discontinuation.

With 24 volunteers per treatment group, a two-sided, 0.05 level binomial test of 75% adherence will have only 29% power to detect a true adherence rate of 90%. Power increases to 66% if the true adherence rate is 95%.

If we combine the intermittent FTC/TDF and intermittent placebo groups (n=36), then a two-sided, 0.05 level binomial test of 75% adherence will have 51% power to detect a true adherence rate of 90%. Power increases to 90% if the true adherence rate is 95%. Likewise, if we combine the daily FTC/TDF and the daily placebo groups (n=36), we will have reasonable power to detect high rates of adherence.

The relationship between adherence and intracellular drug level will be evaluated in the volunteers assigned to active regimens FTC/TDF. Determining the relationship between adherence and intracellular drug levels may provide adherence estimates for future studies which do not include detailed objective adherence measures. The correlation between mean adherence execution over the prior month and intracellular drug level will be tested using Pearson’s correlation coefficient. Mean drug levels in individuals with ≥90% adherence will be compared with those with <90% adherence using a student’s T test.

14.2.5 Intracellular drug concentration

Assuming Week 16 intracellular drug concentration has a log-normal distribution with a between subject standard deviation of 1.0 on the natural logarithm scale, this study should have about 79% power to rule-out a two-fold or greater relative decrease in the geometric mean concentration due to intermittent versus daily FTC/TDF. If the between subject standard deviation is 0.5, then there will be about 80% power to rule-out a 1.5-fold or greater decrease in the geometric mean concentration.

Pharmacokinetic (PK) modelling using plasma and intracellular drug concentrations will be performed to estimate individual PK parameters based on 2 drug levels following an observed dose, body weight, and creatinine clearance, and informed by population-PK parameter estimates. The MEMS data will be combined with the individual PK estimates to simulate concentration-time relationships for the study. The PK-MEMS simulated concentrations will be compared to observed concentrations to evaluate plausibility of the reported dosing behaviour in comparison to that which is observed. Individual estimates of mean trough concentrations (plasma and intracellular) will be estimated. Comparisons between intermittent and daily regimen will be compared using actual and simulation-based values.

Intracellular drug levels will be correlated with pill count and self report data for those volunteers reporting drug use in the past 48 hours. Levels of drug in hair samples will be evaluated for exploratory purposes only.

14.2.6 HIV risk behaviour change

HIV risk behaviour will be measured at each visit by the number of steady and casual partners reported in the previous month, frequency of unprotected sex acts, and substance use prior to or during sex. Methods for repeated measurements, such as linear mixed effects models, will be applied to these data to assess both individual and group level changes in HIV risk behaviour. These models will incorporate time on and off study treatment.

Assuming condoms are used always or frequently by 40% of volunteers at baseline, this study has 46% power to detect a decrease in condom usage by treatment group (n=24) if volunteers reduce the rate to 20%. Power is based on a two-sided, 0.05 level test of a binomial proportion.

14.2.7 Medication sharing

Medicine sharing will be assessed by follow-back interview. Medicine sharing between study volunteers will also be assessed by intracellular drug levels in the placebo recipients.

Levels of TDF in hair samples of study volunteers assigned to FTC/TDF groups will be evaluated for exploratory purposes only.

14.2.8 HIV-specific immune response

HIV-specific immune responses will be measured at timepoints as specified in the Schedule of Procedures (Appendix A). A volunteer will be considered to have a positive response if he/she has at least one positive test result prior to HIV infection. Fisher's exact test will be used to test for any difference in the response rate between intermittent or daily FTC/TDF and placebo recipients. Assuming a 10% HIV-specific immune response rate in the combined control group, this study will have about 83% power to detect an increased response rate due to intermittent PrEP if the true response rate is 50% in the intermittent FTC/TDF group.

15.0 QUALITY CONTROL AND QUALITY ASSURANCE

To ensure the quality and reliability of the data gathered and the ethical conduct of this study, a Study Operations Manual has been developed.

Regular monitoring will be performed according to ICH-GCP as indicated in Section 17.3.

An independent audit of the study may be performed, at the discretion of the sponsor.

By signing the protocol, the Principal Investigators agree to facilitate study related monitoring, audits, IRB/IEC review and regulatory inspection(s) and direct access to source documents. Such information will be treated as strictly confidential and under no circumstances be made publicly available.

16.0 DATA AND BIOLOGICAL MATERIAL

All data and biological material collected through the study shall be managed in accordance with the Clinical Trial Agreement. Distribution and use of those data will be conducted by agreement of both parties.

The computerized raw data generated will be held by the DCC on behalf of the sponsor. The study research centre will also hold the final data files and tables generated for the purpose of analysis. Principal investigators or designees will have access to the clinical study database with appropriate blinding.

17.0 ADMINISTRATIVE STRUCTURE

The Principal Investigator will be responsible for all aspects of the study at the study research centre.

17.1 Safety Review Board (SRB)

The SRB will oversee the progress of the study. The SRB will consist of independent clinicians/scientists/statisticians who are not involved in the study. Investigators responsible for the clinical care of volunteers or representative of the sponsor may not be a member of the SRB.

However, the SRB may invite the Principal Investigators or designees and a sponsor representative to an open session of the meeting to provide information on study conduct, present data or to respond to questions.
The review of study data by the SRB will take place after approximately 25% of volunteers have been followed for 2 months and may also be specifically requested (see Section 17.1.2 Indications for discontinuation of administration of investigational product in all volunteers). Enrolment will continue during this review.

17.1.1 Content of Interim Review

The SRB will be asked to review the following data:

- A blinded listing of all severe clinical adverse events, independent of relationship to the Investigational Product, and
- A blinded listing of all severe laboratory adverse events confirmed on retest, independent of relationship to the Investigational Product, and
- A blinded listing of all Serious Adverse Events, independent of relationship to the Investigational Product.

17.1.2 Indications for discontinuation of administration of investigational product in all Volunteers

If 2 or more of the volunteers participating in this study develop an SAE judged definitely or probably related to the investigational product, the Principal Investigators or designees and the sponsor will request a review by the SRB. The study will be suspended pending a review of all safety data by the SRB. The study may be unblinded at the discretion of the SRB.

Following this review, the SRB will make a recommendation to the Sponsor and the Principal Investigators regarding the continuation of the study.

17.2 Study Supervision

The investigators and the IAVI Medical Monitor will be provided progress report(s) of this study. Close cooperation between members of the study team will be necessary to track study progress, respond to queries about proper study implementation and management, address issues in a timely manner, and assure consistent documentation, and effective information sharing. Rates of accrual, retention, and other parameters relevant to the research centre's performance will be regularly and closely monitored by the study team.

17.3 Study Monitoring

Monitoring will be conducted to ensure that the study is conducted in compliance with human subjects and other research regulations and guidelines, recorded and reported in accordance with the protocol, is consistent with locally-accepted HIV counselling practices, standard operating procedures, Good Clinical Practice (GCP) and applicable regulatory requirements.

The monitor will confirm the quality and accuracy of data at the research centre by validation CRFs against the source documents such as clinical records and against the database when applicable. The Investigators and volunteers, by giving consent, agree that the monitor may inspect study facilities and source records (e.g., informed consent forms, clinic and laboratory records, other source documents), as well as observe the performance of study procedures. Such information will be treated as strictly confidential and will under no circumstances be made publicly available.

The monitoring will adhere to Good Clinical Practice guidelines. The Principal Investigator will permit inspection of the facilities and all study-related documentation by authorized representatives of IAVI, and Government and Regulatory Authorities relevant to this study.

17.4 Investigator's Records

Study records include administrative documentation—including reports and correspondence relating to the study—as well as documentation related to each

volunteer screened for and/or enroled in the study—including informed consent forms, case report forms, and all other source documents. The investigator will maintain and store, in a secure manner, complete, accurate, and current study records for a minimum of 2 years after marketing application approval or the study is discontinued and applicable national and local health authorities are notified or the product development is discontinued. IAVI will notify the Principal Investigator of these events.

18.0 INDEMNITY

The Sponsor and collaborating research centre are responsible to have appropriate liability insurance. For research-related injuries and/or medical problems determined to result from receiving the investigational product, treatment including necessary emergency treatment and proper follow-up care will be made available to the volunteer free of charge at the expense of the Sponsor.

19.0 PUBLICATION

A primary manuscript describing safety and immune responses in this trial will be prepared promptly after the data analysis is available, based on the data compiled by the IAVI statistical centre. Authors will be representatives of each trial research centre, the statistical centre, the laboratories and IAVI, subject to the generally accepted criteria of contributions to the design, work, analysis and writing of the study. Precedence will be given to authors from the research centre enroling the greatest number of volunteers. Manuscripts will be reviewed by representatives of each participating group as specified in the clinical trial agreement (CTA).

20.0 ETHICAL CONSIDERATIONS

The Principal Investigator will ensure that the study is conducted in compliance with the protocol, Standard Operating Procedures in accordance with guidelines laid down by the International Conference on Harmonisation for Good Clinical Practice in clinical studies, the ethical principles that have their origins in the Declaration of Helsinki and applicable regulatory requirements.

In addition to IEC/IRB and regulatory approvals, all other required approvals will be obtained before recruitment of volunteers.

REFERENCES

1. UNAIDS. AIDS epidemic update: December 2007
2. UNAIDS. Implications of HIV variability for transmission: scientific and policy issues. Expert Group of the Joint United Nations Programme on HIV/AIDS. AIDS 1997; 11: UNAIDS1-15.
3. Centers for Disease Control and Prevention 2001. HIV/AIDS surveillance report. *MMWR*:1-48.
4. Towards universal access; scaling up priority HIV/AIDS interventions in the health sector: progress report, April 2007. World Health Organization, UNAIDS, UNICEF.
5. Barouch DH. Challenges in the development of an HIV-1 vaccine. *Nature* 2008; 455:613-9.
6. Grant RM, Hamer D, Hope T, Johnston R, Lange J, Lederman MM et al. Whither or wither microbicides? *Science* 2008; 321:532-4.
7. Antiretroviral Therapy Cohort Collaboration. Life expectancy of individuals on combination antiretroviral therapy in high-income countries: a collaborative analysis of 14 cohort studies. *Lancet* 2008; 372:293-9.
8. Guay LA, Musoke P, Feling T el al. Intrapartum and neonatal single-dose nevirapine compared with zidovudine for prevention of mother-to-child transmission of HIV-1 in Kampala, Uganda: HIVNET 012 randomized trial. *Lancet* 1999; 354:795-802.
9. Dabis F, Leroy V, Castetbon K, Spira R, Newell NL, Salamon R. Preventing mother-to-child transmission of HIV-1 in Africa in the year 2000. *AIDS* 2000;14:1017-26.
10. Scarlatti G. Mother-to-child transmission of HIV-1: advances and controversies of the twentieth centuries. *AIDS Rev* 2004;6:67-78.
11. Panlilio AL, Cardo DM, Grohskopf LA, Heneine W, Ross CS. Updated US Public Health Service guidelines for the management of occupational exposures to HIV and recommendations for postexposure prophylaxis. *MMWR Recomm Rep* 2005;54:1-17.
12. Cardo DM, Culver DH, Srivastava PU, Marcus R, Abiteboul D et al. A case-control study of HIV seroconversion in health care workers after percutaneous exposure. Centers for Disease Control and Prevention Needlestick Surveillance Group. *N Eng J Med* 1997;337:1485-90.
13. Smith DK, Grohskopf LA, Black RJ, Auerbach JD, Veronese F, Struble KA et al. Antiretroviral postexposure prophylaxis after sexual, injection-drug use, or other nonoccupational exposure to HIV in the United State: recommendations from the US Department of Health and Human Services. *MMWR Recomm Rep* 2005;54:1-20.
14. Giele CM, Maw R, Carne CA, Evans BG. Post-exposure prophylaxis for non-occupational exposure to HIV: current clinical practice and opinions in the UK. *Sex Transm Infect* 2002;78:130-2.
15. Almeda J, Casabona J, Allepuz A, Garcia-Alcaide F, del Romero J, Tural C, et al. [Recommendations for non-occupational postexposure HIV prophylaxis. Spanish Working Group on Non-Occupational Postexposure HIV Prophylaxis of the Catalonian Center for Epidemiological Studies on AIDS and the AIDS Study Group]. *Enferm Infecc Microbiol Clin* 2002;20:391-400.
16. European Project on Non Occupational Post Exposure Prophylaxis for HIV. Management of non-occupational post exposure prophylaxis to HIV (NONOPEP): sexual, injecting drug user or other exposures. Accessed at www.inmi.it/news/LineeGuida/RecommendationsNONOCC.htm on 20 October 2008.
17. Rey D, Bendiane MK, Moatti JP, Wellings K, Danziger R, MacDowall W: European Study Group on HIV Testing Policies and Practices in Europe. Post-expsoure prophylaxis after occupational and non-occupational exposures to HIV: an overview of the policies implemented in 27 European countries. AIDS Care 2000;12:695-701.

18. Bernasconi E, Jost J, Ledergerber B, Hirschel B, Francioli P, Sudre P. Antiretroviral prophylaxis for community exposure to the human immunodeficiency virus in Switzerland, 1997-2000. *Swiss Med Wkly* 2001;131:433-7.
19. Vitoria M, Beck E, Madalia S, Diepart M, Stint T, Crowle S, et al. Guidelines for post-exposure prophylaxis for HIV in developing countries [Abstract]. Presented at the 13th Conference on Retroviruses and Opportunistic Infections, Denver, Colorado, 5–8 February 2006: abstract no. 151.
20. Roland ME, Neilands TB, Krone MR, Katz MH, Franses K, Grant RM, et al. Seroconversion following nonoccupational postexposure prophylaxis against HIV. *Clin Infect Dis* 2005; 41:1507-13.
21. Tsai CC, Follis KE, Sabo A, Beck TW, Grant RF, Bischofberger N, et al. Prevention of SIV infection in macaques by (R)-9-(2-phosphonylmethoxypropyl) adenine. *Science* 1995;270:1197-9.
22. Tsai CC, Emau P, Follis KE, Beck TW, Benveniste RE, Bischofberger N, et al. Effectiveness of postinoculation (R)-9-(2-phosphonylmethoxypropyl) adenine treatment for prevention of persistent simian immunodeficiency virus SIVmne infection depends critically on timing of initiation and duration of treatment. *J Virol* 1998;72:4265-73.
23. Le Grand R, Vaslin B, Larghero J, Neidez O, Thiebot H, Sellier P, et al. Post-exposure prophylaxis with highly active antiretroviral therapy could not protect macaques from infection with SIV/HIV chimera. *AIDS.* 2000;14:1864-6.
24. Otten RA, Smith DK, Adams DR, Pullium JK, Jackson E, Kim CN, et al. Efficacy of postexposure prophylaxis after intravaginal exposure of pig-tailed macaques to a human-derived retrovirus (human immunodeficiency virus type 2). *J Virol* 2000;74:9771-5.
25. Cohen MS, Hosseinipour M, Kashuba A, Butera S. Use of antiretroviral drugs to prevent sexual transmission of HIV. *Curr Clin Top Infect Dis* 2002;22:214-51.
26. Van Rompay KK, Schmidt KA, Lawson JR, Singh R, Bischofberger N, Marthas ML. Topical administration of low-dose tenofovir disoproxil fumarate to protect infant macaques against multiple oral exposures of low doses of simian immunodeficiency virus. *J Infect Dis* 2002;186:1508-13.
27. Van Rompay KK, McChesney MB, Aguirre NL, Schmidt KA, Bischofberger N, Marthas ML. Two low doses of tenofovir protect newborn macaques against oral simian immunodeficiency virus infection. *J Infect Dis* 2001;184:429-38.
28. Subbarao S, Otten RA, Ramos A, Kim C, Jackson E, Monsour M, et al. Chemoprophylaxis with tenofovir disoproxil fumarate provided partial protection against infection with simian human immunodeficiency virus in macaques given multiple virus challenges. *J Infect Dis* 2006;194:904-11.
29. Garcia-Lerma J, Otten R, Qari S, Jackson E, Cong M, Masciotra S Prevention of rectal SHIV transmission in macaques by daily or intermittent prophylaxis with FTC and tenofovir, *PLoS Med* 2008;5:e28.
30. Derdelinckx I, Wainberg MA, Lange JM, Hill A, Halima Y, Boucher CA. Criteria for drugs used in pre-exposure prophylaxis trials against HIV infection. *PLoS Med* 2006;3:e454.
31. Panel on Antiretroviral Guidelines for Adults and Adolescents. Guidelines for the use of antiretroviral agents in HIV-1-infected adults and adolescents. Department of Health and Human Services. January 29, 2008; 1-128. Available at http://www.aidsinfo.nih.gov/ContentFiles/AdultandAdolescentGL.pdf. Accessed 20 October 2008.
32. Kearney BP, Isaacson E, Sayre J, Cheng AK. Tenofovir DF and Oral Contraceptives: Lack of a Pharmacokinetic Drug Interaction. Presented at Intersci Conf Antimicrob Agents Chemother 2003 Sep 14-17; 43: abstract no. A-1618.

33. Truvada Investigator's Brochure. Second Edition, 17 Nov 2005. Gilead Sciences Inc., South San Francisco.
34. Wang LH, Begley J, St Claire RL 3rd, Harris J, Wakeford C, Rousseau FS. Pharmacokinetic and pharmacodynamic characteristics of FTC support its once daily dosing for the treatment of HIV infection. *AIDS Res Hum Retroviruses* 2004;20:1173-82.
35. Kearney BP, Flaherty JF, Shah J. Tenofovir Disoproxil Fumarate Clinical Pharmacology and Pharmacokinetics. *Clin Pharmacokinet* 2004;43:595-612.
36. Vourvahis M, Tappouni H, Patterson K, Chen Y, Rezk N, Fiscus S et al. The pharmacokinetics and viral activity of tenofovir in the male genital tract. *J Acquir Immune Defic Syndr* 2008; 329-333.
37. Cohen MS, Gay C, Kashuba A, Blower S, Paxto*n* L. Antiretroviral Therapy to Prevent the Sexual Transmission of HIV-1. *Ann Int Med* 2007;146:591-601.
38. Vourvahis M, Tappouni H, Patterson K, Chen YC, Rezk N, Fiscus S et al. A pharmacologic basis for the use of tenofovir in pre- and post-exposure prophylaxis: intra and extracellular genital tract pharmacokinetics and pharmacodynamics from first dose to steady state in HIV-1-infected men and women [Abstract]. Presented at the 13th Conference on Retroviruses and Opportunistic Infections, Denver, Colorado, 5–8 February 2006: abstract no. 569.
39. Reddy S, Troiani L, Kim J, Rodriguez J, Fiscus S, Kempen AV et al. Differential phosphorylation of zidovudine and lamivudine between semen and blood mononuclear cells in HIV-1 infected men [Abstract]. Presented at the 10th Conference on Retroviruses and Opportunistic Infections, Boston, Massachusetts,10–14 February 2003: abstract no. 530.
40. Angela Kashuba, personal communication.
41. Emtriva Investigator's Brochure. Tenth Edition, 26 Apr 2007. Gilead Sciences Inc., South San Francisco.
42. Whitcomb JM, Parkin NT, Chappey N, Hellman N, Petropolous CJ. Broad nucleoside cross-resistance in human immunodeficiency virus type 1 clinical isolates. J Infect Dis 188:992-1000.
43. Viread Investigator's Brochure. Eleventh Edition, 25 Sept 2006. Gilead Sciences Inc., South San Francisco.
44. Van Rompay K, Durand-Gasselin L, Brignolo LL, Ray AS, Abel K, Cihlar T et al. Chronic administration of tenofovir to rhesus macaques from infancy through adulthood and pregnancy: Summary of pharmacokinetics and biological and virological effects. *Antimicrob Agents Chemo* 2008;52:3144-3160.
45. Mondy K and Tebas P. Emerging bone problems in patients infected with human immunodeficiency virus. *CID* 2003;36 (supple2):S101-105
46. Gallant JE, Staszewski S, Pozniak AL et al.Efficacy and safety of tenofovir DF vs. stavudine in combination therapy in antiretroviral-naïve patients. A 3-year randomized trial. *JAMA* 2004;292:191-201.
47. Nelson M, Katlama C, Montaner J, Cooper D, Gazzard B, Clotet B et al. The safety of tenofovir disoproxil fumarate for the treatment of HIV infection in adults: the first 4 years. *AIDS* 2007;21:1273–1281.
48. Sax PE, Gallant JE, Klotman PE. Renal safety of tenofovir disoproxil fumarate. *AIDS Read* 2007;17:90-C3.
49. Izzedine H, Isnard-Bagnis C, Hulot JS et al. Renal safety of tenofovir in HIV treatment-experienced patients. *AIDS* 2004;18:1074-6.
50. Schooley RT, Ruane P, Myers RA et al; Study 902 Team. Tenofovir DF in antiretroviral-experienced patients: results from a a 48-week, randomized, double-blind study. *AIDS* 2002;16:1257-63.

51. Squires K, Pozniak AL, Pierone G et al; Study 907 Team. Tenofovir disoproxil fumarate in nucleoside-resistance HIV-1 infection. *Ann Intern Med* 2003;139:313-21.
52. Pozniak AL, Gallant JE, DeJesus E et al; Study 934 Group. Tenofovir disoproxil fumarate, FTC, and efavirenz versus fixed dose zidoviudine/lamivudine and efavirenz in antiretroviral-naïve patients: virologic, immunologic, and morphologic changes – a 96-week analysis. *J Acquir Immune Defic Syndr* 2006;43:535-40.
53. Izzedine H, Hulot JS, Vittecoq D et al; Study 903 Team. Long-term renal safety of tenofovir disoproxil fumarate in antiretroviral-naïve HIV-1 infected patients. Data from a boule-blind randomized active-controlled multicentre study. *Nephrol Dial Transplant* 2005;20:743-6.
54. Staszewski S, Pozniak AL, Lu B et al. Similar renal safety profile between tenofovir DF and stavudine using Modification of Diet in Renal Disease (MDRD) and Cockcroft-Gault (CG) estimation of glomerular filtration rate in antiretroviral-naïve patients through 144 weeks. 7th International Workshop on Adverse Drug Reactions and Lipodystrophy in HIV; November 13-16, 2005;Dublin. Abstract 93.
55. Gallant JE, Winston JA, DeJesus E, Pozniak AL, Chen SS, Cheng AK, Enejosa JV. The 3-year renal safety of a tenofovir disoproxil fumarate vs. a thymidine analogue-containing regimen in antiretroviral-naïve patients. *AIDS* 2008;22:2155-63.
56. Reid A, Stohr W, Walker S, Williams IG, Kityo C, Hughes P et al. Severe renal dysfunction and risk factors associated with renal impairment in HIV-infected adults in Africa initiating antiretroviral therapy. *Clin Infect Dis* 2008;46:1271-81.
57. Gayet-Ageron A, Ananworanich J, Jupimai T, Chetchotisakd P, Prasithsirikul W, Ubolyam S, et al. No change in calculated creatinine clearance after tenofovir initiation among Thai patients. *J Antimicrob Chemother* 2007; 59:1034–1037.
58. Gupta SK, Eustace JA, Winston JA et al. Guidelines for the management of chronic kidney disease in HIV-infected patients: recommendations of the HIV Medicine Association of the Infectious Disease Society of America. *Clin Infect Dis* 2005;1559-85.
59. Olmscheid B, Zhang S. Use of Tenofovir Disoproxil Fumarate (TDF) in Pregnancy: Findings from the Antiretroviral Pregnancy Registry (APR). ICAAC/IDSA 46th Annual Meeting. October 2008, Washington DC. (Abstract H-456)
60. Galluzzo CM, Germinario EA, Bassani L, Mancini MG, Okong P et al. Antiretroviral resistance mutations in untreated pregnant women with HIV infection in Uganda and Rwanda. *AIDS Res Hum Retroviruses* 2007;23:1449-1451.
61. Vessiere A, Nerrienet E, Kfutwah A, Menu E, Tejiokem M et al. HIV-1 pol gene polymorphism and antiretroviral resistance mutations in drug-naive pregnant women in Yaounde, Cameroon. *J Acquir Immune Defic Syndr* 2006;42: 256-258.
62. Bussmann H, Novitsky V, Wester W, Peter T, Masupu K et al. HIV-1 subtype C drug-resistance background among ARV-naive adults in Botswana. *Antivir Chem Chemother 2005;*16:103-115.
63. Toni T, Masquelier B, Minga A, Anglaret X, Danel C et al. HIV-1 antiretroviral drug resistance in recently infected patients in Abidjan, Cote d'Ivoire: A 4-year survey, 2002-2006. *AIDS Res Hum Retroviruses* 2007;23:1155-1160.
64. Bellocchi MC, Forbici F, Palombi L, Gori C, Coelho E et al. Subtype analysis and mutations to antiviral drugs in HIV-1-infected patients from Mozambique before initiation of antiretroviral therapy: results from the DREAM programme. *J Med Virol* 2005;76:452-458.
65. UK Collaborative Group on Monitoring the Transmission of HIV Drug Resistance. Analysis of prevalence of HIV-1 drug resistance in primary infections in the United Kingdom. *BMJ* 2001;322:1087-1088.
66. Grant RM, Hecht FM, Warmerdam M, Liu L, Liegler T et al. Time trends in primary HIV-1 drug resistance among recently infected persons. *JAMA* 2002;88:181-188.

67. Little SJ, Holte S, Routy JP, Daar ES, Markowitz M, et al. Antiretroviral-drug resistance among patients recently infected with HIV. *N Engl J Med* 2002;347:385-394.
68. Smith D, Kebaabetswe P, Disasi K, Fleming D, Paxton L, Davis M. Antiretroviral resistance is not an important risk of the oral tenofovir prophylaxis trial in Botswana: a simple mathematical modelling approach. 2006. Abstract THAX0105. AIDS 2006 - XVI International AIDS Conference. Toronto.
69. Peterson L, Taylor D, Roddy R, Belai G, Phillips P, Nanda K et al. Tenofovir Disoproxil Fumarate for Prevention of HIV Infection in Women: A Phase 2, Double-Blind, Randomized, Placebo-Controlled Trial. *PLoS Clin Trials* 2(5): e27.
70. CDC Trials of Pre-exposure Prophylaxis for HIV Prevention. July 2008. Available at: http://www.cdc.gov/HIV/resources/factsheets/prep.htm. Accessed on 20 October 2008.
71. AIDS Vaccine Advocacy Coalition. Fact sheet: Pre-exposure prophylaxis (PrEP) for HIV prevention. August 2008. Available at: http://www.avac.org/pdf/factsheet_PrEP.pdf. Accessed on 20 October 2008.
72. Byakika-Tusiime J, Oyugi JH, Tumwikirize WA, Katabira ET, Mugyenyi PN, Bangsberg DR. Adherence to HIV antiretroviral therapy in HIV+ Ugandan patients purchasing therapy. *Int J STD AIDS* 2005,16:38-41.
73. Laurent C, Diakhate N, Gueye NF*, et al.* The Senegalese government's highly active antiretroviral therapy initiative: an 18-month follow-up study. *Aids* 2002,16:1363-1370.
74. Laurent C, Kouanfack C, Koulla-Shiro S*, et al.* Effectiveness and safety of a generic fixed-dose combination of nevirapine, stavudine, and lamivudine in HIV-1-infected adults in Cameroon: open-label multicentre trial. *Lancet* 2004,364:29-34.
75. Nachega JB, Hislop M, Dowdy DW, Chaisson RE, Regensberg L, Maartens G. Adherence to nonnucleoside reverse transcriptase inhibitor-based HIV therapy and virologic outcomes. *Ann Intern Med* 2007,146:564-573.
76. Orrell C, Bangsberg D, Badri M, Wood R. Adherence is not a barrier to delivery of HIV antiretroviral therapy in resource-poor countries. *AIDS* 2003,17:1369-1375.
77. Oyugi JH, Byakika-Tusiime J, Charlebois ED*, et al.* Multiple Validated Measures of Adherence Indicate High Levels of Adherence to Generic HIV Antiretroviral Therapy in a Resource-Limited Setting. *J Acquir Immune Defic Syndr* 2004,36:1100-1102.
78. Mills EJ, Nachega JB, Buchan I*, et al.* Adherence to antiretroviral therapy in sub-Saharan Africa and North America: a meta-analysis. *Jama* 2006,296:679-690.
79. Weidle PJ, Wamai N, Solberg P*, et al.* Adherence to antiretroviral therapy in a home-based AIDS care programme in rural Uganda. *Lancet* 2006,368:1587-1594.
80. Trial Shows Anti-HIV Microbicide Is Safe, but Does Not Prove It Effective. *POP Council News Release* 2008.
81. Padian NS, van der Straten A, Ramjee G*, et al.* Diaphragm and lubricant gel for prevention of HIV acquisition in southern African women: a randomised controlled trial. *Lancet* 2007,370:251-261.
82. Watson-Jones D, Weiss HA, Rusizoka M*, et al.* Effect of Herpes Simplex Suppression on Incidence of HIV among Women in Tanzania. *N Engl J Med* 2008,358:1560-1571.
83. Celum C, Wald A, Hughes J, et al. HSV-2 Suppressive Therapy for Prevention of HIV Acquisition: Results of HPTN 039. 2008.
84. Celum C. personal communication. 2008.
85. Grymonpre RE, Didur CD, Montgomery PR, Sitar DS. Pill count, self-report, and pharmacy claims data to measure medication adherence in the elderly. *Ann Pharmacother* 1998,32:749-754.
86. Lee JY, Greene PG, Douglas M*, et al.* Appointment attendance, pill counts, and achievement of goal blood pressure in the African American Study of Kidney Disease and Hypertension Pilot Study. *Control Clin Trials* 1996,17:34S-39S.

87. Lee JY, Kusek JW, Greene PG*, et al.* Assessing medication adherence by pill count and electronic monitoring in the African American Study of Kidney Disease and Hypertension (AASK) Pilot Study. *Am J Hypertens* 1996,9:719-725.
88. Matsuyama JR, Mason BJ, Jue SG. Pharmacists' interventions using an electronic medication-event monitoring device's adherence data versus pill counts. *Ann Pharmacother* 1993,27:851-855.
89. Waterhouse DM, Calzone KA, Mele C, Brenner DE. Adherence to oral tamoxifen: a comparison of patient self-report, pill counts, and microelectronic monitoring [see comments]. *J Clin Oncol* 1993,11:1189-1197.
90. Bangsberg. D, Hecht F, Clague H*, et al.* Provider assessment of adherence to HIV antiretroviral therapy. *J Acquir Immune Defic Syndr* 2001,26:435-442.
91. Arnsten JH, Demas PA, Farzadegan H*, et al.* Antiretroviral therapy adherence and viral suppression in HIV-infected drug users: comparison of self-report and electronic monitoring. *Clin Infect Dis* 2001,33:1417-1423.
92. Bangsberg DR, Hecht FM, Charlebois ED*, et al.* Adherence to protease inhibitors, HIV-1 viral load, and development of drug resistance in an indigent population. *AIDS* 2000,14:357-366.
93. Gross R, Yip B, Wood E, et al. Boosted PI are more forgiving of suboptimal adherence than non-boosted PI or NNRTI. CROI. Denver 2006.
94. Oyugi JH, Byakika-Tusiime J, Ragland K, et al. Treatment interruptions predict resistance in HIV-positive individuals purchasing fixed-dose combination antiretroviral therapy in Kampala, Uganda. AIDS 2007,21:965-971.
95. Petersen ML, Wang Y, van der Laan MJ, Guzman D, Riley E, Bangsberg DR. Pillbox organizers are associated with improved adherence to HIV antiretroviral therapy and viral suppression: a marginal structural model analysis. *Clin Infect Dis* 2007,45:908-915.
96. Arnsten JH, Demas PA, Grant RW*, et al.* Impact of active drug use on antiretroviral therapy adherence and viral suppression in HIV-infected drug users. *J Gen Intern Med* 2002,17:377-381.
97. Howard A, Arnsten J, Gardner L*, et al.* Lack of multi-drug resistance in nonresponders to antiretroviral therapy with poor adherence. *1st International AIDS Society Conference on HIV Pathogenesis and Treatment.* Beunos Aires 2001.
98. Howard AA, Arnsten JH, Lo Y*, et al.* A prospective study of adherence and viral load in a large multi-center cohort of HIV-infected women. *Aids* 2002,16:2175-2182.
99. Nieuwkerk PT, Sprangers MA, Burger DM, Hoetelmans RM, Hugen PW, Danner SA, et al. Limited patient adherence to highly active antiretroviral therapy for HIV-1 infection in an observational cohort study. Arch Intern Med 2001, 161: 1962–1968.
100. Hugen PW, Langebeek N, Burger DM, Zomer B, van Leusen R, Schuurman R, et al. Assessment of adherence to HIV protease inhibitors: comparison and combination of various methods, including MEMS (electronic monitoring), patient and nurse report, and therapeutic drug monitoring. J Acquir Immune Defic Syndr 2002, 30:324–334.
101. Gandhi M, Ameli N, Bacchetti P, Ponath C, Benet LZ, Baluom M, et al. Poor correlation between self-reported adherence to antiretrovirals and plasma drug levels. In: XIVth International AIDS Conference. Barcelona, Spain, 2002 [Abstract 5917].
102. van Rossum AM, Bergshoeff AS, Fraaij PL, Hugen PW, Hartwig NG, Geelen SP, et al. Therapeutic drug monitoring of indinavir and nel.navir to assess adherence to therapy in human immunodeficiency virus-infected children. Pediatr Infect Dis J 2002, 21:743–747.
103. Podsadecki TJ, Vrijens BC, Tousset EP, Rode RA, Hanna GJ. "White coat compliance" limits the reliability of therapeutic drug monitoring in HIV-1-infected patients. HIV Clin Trials. 2008 Jul-Aug;9(4):238-46. PMID: 18753118 [PubMed - indexed for MEDLINE]
104. Liechty CA, Alexander CS, Harrigan PR, Guzman JD, Charlebois ED, Moss AR,

Bangsberg DR. Are untimed antiretroviral drug levels useful predictors of adherence behavior? AIDS. 2004 Jan 2;18(1):127-9. PMID: 15090840 [PubMed - indexed for MEDLINE]
105. Antinori A, Cozzi-Lepri A, Ammassari A, et al. Relative prognostic value of self-reported adherence and plasma NNRTI/PI concentrations to predict virological rebound in patients initially responding to HAART. Antivir Ther 2004,9:291-296.
106. Gifford AL, Bormann JE, Shively MJ, Wright BC, Richman DD, Bozzette SA. Predictors of self-reported adherence and plasma HIV concentrations in patients on multidrug antiretroviral regimens [In Process Citation]. *J Acquir Immune Defic Syndr* 2000,23:386-395.
107. Miller LG, Hays RD. Measuring adherence to antiretroviral medications in clinical trials. *HIV Clin Trials* 2000,1:36-46.
108. Miller LG, Liu H, Hays RD*, et al.* How well do clinicians estimate patients' adherence to combination antiretroviral therapy? *J Gen Intern Med* 2002,17:1-11.
109. Simoni JM, Kurth AE, Pearson CR, Pantalone DW, Merrill JO, Frick PA. Self-report measures of antiretroviral therapy adherence: A review with recommendations for HIV research and clinical management. *AIDS Behav* 2006,10:227-245.
110. Walsh JC, Mandalia S, Gazzard BG. Responses to a 1 month self-report on adherence to antiretroviral therapy are consistent with electronic data and virological treatment outcome. *Aids* 2002,16:269-277.
111. Lee JY, Kusek JW, Greene PG, et al. Assessing medication adherence by pill count and electronic monitoring in the African American Study of Kidney Disease and Hypertension (AASK) Pilot Study. Am J Hypertens 1996,9:719-725.
112. Arnet I, Haefeli WE. Overconsumption detected by electronic drug monitoring requires subtle interpretation. *Clin Pharmacol Ther* 2000,67:44-47.
113. Farmer KC. Methods for measuring and monitoring medication regimen adherence in clinical trials and clinical practice. *Clin Ther* 1999,21:1074-1090; discussion 1073.
114. Fletcher SW, Pappius EM, Harper SJ. Measurement of medication compliance in a clinical setting. Comparison of three methods in patients prescribed digoxin. *Arch Intern Med* 1979,139:635-638.
115. Kastrissios H, Suarez JR, Katzenstein D, Girard P, Sheiner LB, Blaschke TF. Characterizing patterns of drug-taking behavior with a multiple drug regimen in an AIDS clinical trial. *Aids* 1998,12:2295-2303.
116. Matsui D, Hermann C, Klein J, Berkovitch M, Olivieri N, Koren G. Critical comparison of novel and existing methods of compliance assessment during a clinical trial of an oral iron chelator. *J Clin Pharmacol* 1994,34:944-949.
117. Liu H, Golin CE, Miller LG*, et al.* A comparison study of multiple measures of adherence to HIV protease inhibitors. *Ann Intern Med* 2001,134:968-977.
118. Golin CE, Liu H, Hays RD*, et al.* A prospective study of predictors of adherence to combination antiretroviral medication. *J Gen Intern Med* 2002,17:756-765.
119. Bangsberg D, Hecht F, Charlebois E, Chesney M, Moss A. Comparing Objectives Measures of Adherence to HIV Antiretroviral Therapy: Electronic Medication Monitors and Unannounced Pill Counts. *AIDS and Behavior* 2001,5:275-281.
120. Bangsberg D, K R, Hahn JA*, et al.* No Difference in Adherence or Viral Suppression to Ritonavir Boosted Protease Inhibitor(r-PI) and Non-Nucleoside Reverse Transcriptase Inhibitor (NNRTI) Regimens. *2nd International Conference on HIV Treatment Adherence*. Jersey City, New Jersey 2007.
121. Bangsberg D, Kroetz D, Deeks S. Adherence-resistance Relationships to Combination HIV Antiretroviral Therapy. *Current HIV/AIDS Reports* 2007,4:65-72.
122. Bangsberg DR. Less than 95% adherence to nonnucleoside reverse-transcriptase inhibitor therapy can lead to viral suppression. *Clin Infect Dis* 2006,43:939-941.

123. Bangsberg DR, Bronstone A, Hofmann R. A computer-based assessment detects regimen misunderstandings and nonadherence for patients on HIV antiretroviral therapy. *AIDS Care* 2002,14:3-15.
124. Bangsberg DR, Charlebois ED, Grant RM*, et al.* High levels of adherence do not prevent accumulation of HIV drug resistance mutations. *Aids* 2003,17:1925-1932.
125. Giordano T, Guzman D, Clark R, Charlebois E, Bangsberg D. Measuring Adherence to Antiretroviral Therapy in a Diverse Population Using a Visual Analogue Scale. *HIV Clin Trials* 2004,5:74-79.
126. Gross R, Yip B, Lo Re V, 3rd*, et al.* A simple, dynamic measure of antiretroviral therapy adherence predicts failure to maintain HIV-1 suppression. *J Infect Dis* 2006,194:1108-1114.
127. Emau P, Jiang Y, Agy MB, Tian B, Bekele G, Tsai CC. Post-exposure prophylaxis for SIV revisited: animal model for HIV prevention. AIDS Res Ther 2006,3:29.
128. Mundt JC. Interactive voice response systems in clinical research and treatment. *Psychiatr Serv* 1997,48:611-612.
129. Corkrey R, Parkinson L. A comparison of four computer-based telephone interviewing methods: getting answers to sensitive questions. *Behav Res Methods Instrum Comput* 2002,34:354-363.
130. Corkrey R, Parkinson L. Interactive voice response: review of studies 1989-2000. *Behav Res Methods Instrum Comput* 2002,34:342-353.
131. Perrine MW, Mundt JC, Searles JS, Lester LS. Validation of daily self-reported alcohol consumption using interactive voice response (IVR) technology. *J Stud Alcohol* 1995,56:487-490.
132. Searles JS, Perrine MW, Mundt JC, Helzer JE. Self-report of drinking using touch-tone telephone: extending the limits of reliable daily contact. *J Stud Alcohol* 1995,56:375-382.
133. Searles JS, Helzer JE, Walter DE. Comparison of drinking patterns measured by daily reports and timeline follow back. *Psychol Addict Behav* 2000,14:277-286.
134. Turvey CL, Willyard D, Hickman DH, Klein DM, Kukoyi O. Telehealth screen for depression in a chronic illness care management program. *Telemed J E Health* 2007,13:51-56.
135. Mundt JC, Kobak KA, Taylor LV*, et al.* Administration of the Hamilton Depression Rating Scale using interactive voice response technology. *MD Comput* 1998,15:31-39.
136. Greist JH, Marks IM, Baer L*, et al.* Self-treatment for obsessive compulsive disorder using a manual and a computerized telephone interview: a U.S.-U.K. study. *MD Comput* 1998,15:149-157.
137. Burke A. Examining the use of a fully-automated interactive voice response tobacco cessation support line. *Am J Health Promot* 1993,8:93-94, 100.
138. Toll BA, Cooney NL, McKee SA, O'Malley SS. Do daily interactive voice response reports of smoking behavior correspond with retrospective reports? *Psychol Addict Behav* 2005,19:291-295.
139. Gee P, Coventry KR, Birkenhead D. Mood state and gambling: using mobile telephones to track emotions. *Br J Psychol* 2005,96:53-66.
140. Schroder KE, Johnson CJ, Wiebe JS. Interactive Voice Response Technology applied to sexual behavior self-reports: a comparison of three methods. *AIDS Behav* 2007,11:313-323.
141. Weinhardt LS, Carey MP, Maisto SA, Carey KB, Cohen MM, Wickramasinghe SM. Reliability of the timeline follow-back sexual behavior interview. *Ann Behav Med* 1998,20:25-30.
142. Crosby GM, Stall RD, Paul JP, Barrett DC, Midanik LT. Condom use among gay/bisexual male substance abusers using the timeline follow-back method. Addict Behav 1996,21:249-257.

143. Helzer JE, Badger GJ, Rose GL, Mongeon JA, Searles JS. Decline in alcohol consumption during two years of daily reporting. *Journal of Studies on Alcohol* 2002,63:551-558.
144. Lifson JD, Rossio JL, Arnaout R et al. Containment of SIV infection: cellular immune responses and protection from rechallenge following transient post inoculation antiretroviral treatment. *J Virol* 2000; 74:2584-93.
145. Lifson JD, Rossio JL, Piatak M et al. *J Virol* 2001;75:10187-99
146. Rosenwirth B. et al. Antiretroviral therapy during primary immunodeficiency virus infection can induce persistent suppression of virus load and protection from heterologous challenge in rhesus macaques. *J Virol* 2000; 74:1704-11
147. Tsai CC et al. Post-exposure chemoprophylaxis (PECP) against SIV infection of macaques as a model for protection from HIV infection. *J Med Primatol* 2000;29:248-58.
148. Metzner KJ et al. Tenofovir treatment augments anti-viral immunity against drug-resistant SIV challenge in chronically infected rhesus macaques. *Retrovirology* 2006; 21:97
149. Mori K et al. Suppression of acute viremia by short-term post exposure prophylaxis of simian/human immunodeficiency virus SHIV-RT-infected monkeys with a novel reverse transcriptase inhibitor (GW420867) allows for development of potent antiviral immune responses resulting in efficient containment of infection. *J Virol* 2000;74:5747-53.
150. Spring M et al. Enhanced cellular immune response and reduced CD8(+) lymphocyte apoptosis in acutely SIV-infected rhesus macaques after short-term antiretroviral treatment. *Virology* 2001;279:221-32.
151. Wodarz D, Arnaout RA, Nowak MA, Lifson JD Transient antiretroviral treatment during acute SIV infection facilitates long term control of the SIV infection facilitates long term control of the virus. *Philos Trans R Soc Lond B Biol Sci* 2000; 355:1021-9.
152. Cranage M, Sharpe S, Herrera C, Cope A, Dennis M, Berry N et al. Prevention of SIV Rectal Transmission and Priming of T Cell Responses in Macaques after Local Pre-exposure Application of Tenofovir Gel. *PLoS Med* 2008;5(8): e157.
153. Lifson JD et al. Transient early post-inoculation anti-retroviral treatment facilitates controlled infection with sparing of CD4+ T cells in gut-associated lymphoid tissues in SIVmac239-infected rhesus macaques, but not resistance to rechallenge. *J Med Primatol* 2003;32:201-10.
154. Prada N, Davis B, Jean-Pierre P, LaRoche M, Duh F, Carrington M et al. Drug-Susceptible HIV-1 Infection Despite Intermittent Fixed-Dose Combination Tenofovir/FTC as Prophylaxis Is Associated With Low-Level Viremia, Delayed Seroconversion, and an Attenuated Clinical Course. *J Acquir Immune Defic Syndr* 2008;49:117-22.
155. Van Damme L, Govinden R, Mirembe FM, Guédou F, Solomon S, Becker ML, et al. Group.Lack of effectiveness of cellulose sulfate gel for the prevention of vaginal HIV transmission. *N Engl J Med* 2008;359(5):463-72
156. Coetzee N, Hoosen A, Blanchard K, et al. Safety of a lambda carageenan Microbicide (CarraguardTM) in South Africa. Available at: http://www.itg.be/micro2002/downloads/presentations/2Monday_May_13_2002/Track_B_sessions/Nicol_Coetzee.pdf. Accessed 12Nov08.
157. Sanders EJ, Graham SM, Okuku HS, et al. HIV-1 infection in high risk men who have sex with men in Mombasa, Kenya. *AIDS* 2007; 21: 2513-20.
158. Sanders EJ, Graham SM, Okuku HS, van der Elst EM, Muhaari A, Davies A, et al. HIV-1 infection in high risk men who have sex with men in Mombasa, Kenya. AIDS 2007; 21:2513-20.
159. Grijsen ML, Graham SM, Mwangome M, Githua P, Mutimba S, Wamuyu L, et al. Screening for genital and anorectal sexually transmitted infections in HIV prevention trials in Africa. Sex Transm Infect 2008;84:364-70.

160. Sanders EJ, Muhaari A, van der Elst EM, Kowuor D, Davies A, Agwanda C, et al. Measuring sex acts in male sex workers in Kenya: Intermittent Pre-Exposure Prophylaxis (PrEP) may be feasible. AIDS Vaccine Conference 2008. Cape Town, South Africa(Abstract LB-26).
161. Vrijens B, Goetghebeur E. Comparing compliance patterns between randomized treatments. *Control Clin Trials* 1997,18:187-203.
162. Vrijens B, Urquhart J. Patient adherence to prescribed antimicrobial drug dosing regimens. *J Antimicrob Chemother* 2005,55:616-627.
163. Carey MP, Carey KB, Maisto SA, Gordon CM, Weinhardt LS. Assessing sexual risk behaviour with the Timeline Followback (TLFB) approach: continued development and psychometric evaluation with psychiatric outpatients. *Int J STD AIDS* 2001,12:365-375.
164. Sobell LC, Ellingstad TP, Sobell MB. Natural recovery from alcohol and drug problems: methodological review of the research with suggestions for future directions. *Addiction* 2000,95:749-764.

APPENDIX A: SCHEDULE OF PROCEDURES

	Screen	Rando							Final visit/ET	HIV f/u 1	HIV f/u 2
**Study Week**		0	W1	W2	W4	W4+ 24 hrs	W8	W12	W16		
**Visit Windows (Days)**		-42	± 1	± 2	-7/ +2		- 7/ +2	-7/ +2	- 7/+2		
**Investigational Product**											
FTC/TDF/Placebo provision		X			X		X	X			
Drug accountability (pill count/unused pills)					X		X	X	X		
Upload MEMS data					X		X	X	X		
**Consent / Counselling / Assessments / Questionnaires**											
Informed Consent and assessment of understanding	X										
Screening Questionnaire	X										
HIV risk Assessment	X										
Pre-/Post HIV-test counselling	X	X			X		X	X	X	X**	X**
HIV risk reduction counselling / Family planning counselling	X	X			X		X	X		X	X
Adherence counselling		X	X	X	X		X	X			
Follow-back interview (adherence, sexual risk behaviour, medication sharing)			X	X	X		X	X	X		
Acceptability assessment (focus group/individual interview)									X*		
IVR or SMS behavioural assessment	daily										
**Clinical Safety Assessments**											
Medical History including Concomitant Medications	X	X	X	X	X		X	X	X	X	X
General Physical Exam***	X								X	X	
Directed Physical Exam ****		X	X	X	X		X	X			X
Adverse Events/ Serious Adverse Events		X	X	X	X		X	X	X		
**Lab Samples**											
Hep BsAg and STI screening: urine for gonorrhoea and chlamydia; blood for syphilis; rectal swab for chlamydia and gonorrhoea; additionally for females vaginal swab for trichomonas, Candida vaginitis & bacterial vaginosis **.** + Swab to collect mucosal secretions (vaginal and anal) for antibody secretion.	X										
Haematology (CBC) (+ plasma storage)	X				X		X		X	X	
Clinical Chemistry (creatinine, creatinine clearance, pancreatic amylase†, serum phosphate, aspartate transferase (AST), alanine transferase (ALT), total bilirubin	X				X		X		X	X	
Urine dipstick +/- urinalysis	X										
HIV test	X	X			X		X	X	X	X**	X**
Pregnancy Test (all female volunteers)	X	X			X		X	X	X		
Hair samples for adherence assessment							X		X		
Drug plasma and intracellular level					X††	X	X	X	X	X	
PBMCs (antibodies and cellular response against HIV antigens, intracellular drug levels, serum and plasma storage)		X			X		X	X	X	X	X
Sample for HLA typing and related testing		X									
CD4+ count, HIV viral load, p24 antigen, HIV genotyping, viral tropism assay, HIV sequencing										X	

ET = Early Termination
HIV f/u 1 and HIV f/u 2 = Additional visits for HIV infected individuals after discontinuing investigational product to characterize infection and assess for the development of drug resistance mutations.
†Screening visit only
††Blood specimens for plasma and intracellular drug levels collected before and approx. 2 hours after witnessed administration of investigational product as part of the single-dose, timed pharmacokinetic study at Week 4 visit

*Focus group/individual interview will take place as soon as possible after the final study visit.
**If needed to confirm HIV diagnosis
*** General physical examination including height, weight, vital signs (pulse, respiratory rate, blood pressure and temperature), examination of skin, respiratory, cardiovascular and abdominal systems, an assessment of cervical and axillary lymph nodes. At screening, an ano-gential exam for STIs should be included.
****Symptom directed physical examination including vital signs (pulse, respiratory rate, blood pressure and temperature), and any further examination indicated by history or observation

APPENDIX B: ADVERSE EVENT GRADING TOXICITY TABLE

DIVISION OF AIDS TABLE FOR GRADING THE SEVERITY OF ADULT AND PEDIATRIC ADVERSE EVENTS, PUBLISH DATE: DECEMBER, 2004

Quick Reference

The Division of AIDS Table for Grading the Severity of Adult and Pediatric Adverse Events ("DAIDS AE grading table") is a descriptive terminology which can be utilized for Adverse Event (AE) reporting. A grading (severity) scale is provided for each AE term.

General Instructions

Estimating Severity Grade

If the need arises to grade a clinical AE that is not identified in the DAIDS AE grading table, use the category "Estimating Severity Grade" located at the top of Page 3. For AEs that are not listed in the table but will be collected systematically for a study/trial, protocol teams are highly encouraged to define study specific severity scales within the protocol or an appendix to the protocol. (Please see "Template Wording for the Expedited Adverse Event Reporting Section of DAIDS-sponsored Protocols".) This is particularly important for laboratory values because the "Estimating Severity Grade" category only applies to clinical symptoms.

Grading Adult and Pediatric AEs

The DAIDS AE grading table includes parameters for grading both Adult and Pediatric AEs. When a single set of parameters is not appropriate for grading specific types of AEs for both Adult and Pediatric populations, separate sets of parameters for Adult and/or Pediatric populations (with specified respective age ranges) are given in the table. If there is no distinction in the table between Adult and Pediatric values for a type of AE, then the single set of parameters listed is to be used for grading the severity of both Adult and Pediatric events of that type.

Determining Severity Grade

If the severity of an AE could fall under either one of two grades (e.g., the severity of an AE could be either Grade 2 or Grade 3), select the higher of the two grades for the AE.

Definitions

Basic Self-care Functions	Adult<br>Activities such as bathing, dressing, toileting, transfer/movement, continence, and feeding.
	Young Children<br>Activities that are age and culturally appropriate (e.g., feeding self with culturally appropriate eating implement).
LLN	Lower limit of normal
Medical Intervention	Use of pharmacologic or biologic agent(s) for treatment of an AE.
NA	Not Applicable
Operative Intervention	Surgical OR other invasive mechanical procedures.
ULN	Upper limit of normal
Usual Social & Functional Activities	Adult<br>Adaptive tasks and desirable activities, such as going to work, shopping, cooking, use of transportation, pursuing a hobby, etc
	Young Children<br>Activities that are age and culturally appropriate (e.g., social interactions, play activities, learning tasks, etc.).

CLINICAL				
**PARAMETER**	**GRADE 1 MILD**	**GRADE 2 MODERATE**	**GRADE 3 SEVERE**	**GRADE 4 POTENTIALLY LIFE-THREATENING**
**ESTIMATING SEVERITY GRADE**				
Clinical adverse event NOT identified elsewhere in this DAIDS AE grading table	Symptoms causing no or minimal interference with usual social & functional activities	Symptoms causing greater than minimal interference with usual social & functional activities	Symptoms causing inability to perform usual social & functional activities	Symptoms causing inability to perform basic self-care functions OR Medical or operative intervention indicated to prevent permanent impairment, persistent disability, or death
**SYSTEMIC**				
Acute systemic allergic reaction	Localized urticaria (wheals) with no medical intervention indicated	Localized urticaria with medical intervention indicated OR Mild angioedema with no medical intervention indicated	Generalized urticaria OR Angioedema with medical intervention indicated OR Symptomatic mild bronchospasm	Acute anaphylaxis OR Life-threatening bronchospasm OR laryngeal edema
Chills	Symptoms causing no or minimal interference with usual social & functional activities	Symptoms causing greater than minimal interference with usual social & functional activities	Symptoms causing inability to perform usual social & functional activities	NA
Fatigue<br>Malaise	Symptoms causing no or minimal interference with usual social & functional activities	Symptoms causing greater than minimal interference with usual social & functional activities	Symptoms causing inability to perform usual social & functional activities	Incapacitating fatigue/ malaise symptoms causing inability to perform basic self-care functions
Fever (nonaxillary)	37.7 – 38.6°C	38.7 – 39.3°C	39.4 – 40.5°C	> 40.5°C
Pain (indicate body site)<br>DO NOT use for pain due to injection (See Injection Site Reactions: Injection site pain)<br>See also Headache, Arthralgia, and Myalgia	Pain causing no or minimal interference with usual social & functional activities	Pain causing greater than minimal interference with usual social & functional activities	Pain causing inability to perform usual social & functional activities	Disabling pain causing inability to perform basic self-care functions OR Hospitalization (other than emergency room visit) indicated

Basic Self-care Functions – Adult: Activities such as bathing, dressing, toileting, transfer/movement, continence, and feeding.

Basic Self-care Functions – Young Children: Activities that are age and culturally appropriate (e.g., feeding self with culturally appropriate eating implement).

Usual Social & Functional Activities – Adult: Adaptive tasks and desirable activities, such as going to work, shopping, cooking, use of transportation, pursuing a hobby, etc.

Usual Social & Functional Activities – Young Children: Activities that are age and culturally appropriate (e.g., social interactions, play activities, learning tasks, etc.).

CLINICAL				
**PARAMETER**	**GRADE 1 MILD**	**GRADE 2 MODERATE**	**GRADE 3 SEVERE**	**GRADE 4 POTENTIALLY LIFE-THREATENING**
Unintentional weight loss	NA	5 – 9% loss in body weight from baseline	10 – 19% loss in body weight from baseline	≥ 20% loss in body weight from baseline OR Aggressive intervention indicated [e.g., tube feeding or total parenteral nutrition (TPN)]
**INFECTION**				
Infection (any other than HIV infection)	Localized, no systemic antimicrobial treatment indicated AND Symptoms causing no or minimal interference with usual social & functional activities	Systemic antimicrobial treatment indicated OR Symptoms causing greater than minimal interference with usual social & functional activities	Systemic antimicrobial treatment indicated AND Symptoms causing inability to perform usual social & functional activities OR Operative intervention (other than simple incision and drainage) indicated	Life-threatening consequences (e.g., septic shock)
**INJECTION SITE REACTIONS**				
Injection site pain (pain without touching)<br>Or<br>Tenderness (pain when area is touched)	Pain/tenderness causing no or minimal limitation of use of limb	Pain/tenderness limiting use of limb OR Pain/tenderness causing greater than minimal interference with usual social & functional activities	Pain/tenderness causing inability to perform usual social & functional activities	Pain/tenderness causing inability to perform basic self-care function OR Hospitalization (other than emergency room visit) indicated for management of pain/tenderness
Injection site reaction (localized)				
**Adult > 15 years**	Erythema OR Induration of 5x5 cm – 9x9 cm (or 25 cm^2 – 81cm^2)	Erythema OR Induration OR Edema > 9 cm any diameter (or > 81 cm^2)	Ulceration OR Secondary infection OR Phlebitis OR Sterile abscess OR Drainage	Necrosis (involving dermis and deeper tissue)
**Pediatric ≤ 15 years**	Erythema OR Induration OR Edema present but ≤ 2.5 cm diameter	Erythema OR Induration OR Edema > 2.5 cm diameter but < 50% surface area of the extremity segment (e.g., upper arm/thigh)	Erythema OR Induration OR Edema involving ≥ 50% surface area of the extremity segment (e.g., upper arm/thigh) OR Ulceration OR Secondary infection OR Phlebitis OR Sterile abscess OR Drainage	Necrosis (involving dermis and deeper tissue)

Basic Self-care Functions – Adult: Activities such as bathing, dressing, toileting, transfer/movement, continence, and feeding.

Basic Self-care Functions – Young Children: Activities that are age and culturally appropriate (e.g., feeding self with culturally appropriate eating implement).

Usual Social & Functional Activities – Adult: Adaptive tasks and desirable activities, such as going to work, shopping, cooking, use of transportation, pursuing a hobby, etc.

Usual Social & Functional Activities – Young Children: Activities that are age and culturally appropriate (e.g., social interactions, play activities, learning tasks, etc.).

CLINICAL				
**PARAMETER**	**GRADE 1 MILD**	**GRADE 2 MODERATE**	**GRADE 3 SEVERE**	**GRADE 4 POTENTIALLY LIFE-THREATENING**
Pruritis associated with injection<br>See also Skin: Pruritis (itching - no skin lesions)	Itching localized to injection site AND Relieved spontaneously or with < 48 hours treatment	Itching beyond the injection site but not generalized OR Itching localized to injection site requiring ≥ 48 hours treatment	Generalized itching causing inability to perform usual social & functional activities	NA
**SKIN – DERMATOLOGICAL**				
Alopecia	Thinning detectable by study participant (or by caregiver for young children and disabled adults)	Thinning or patchy hair loss detectable by health care provider	Complete hair loss	NA
Cutaneous reaction – rash	Localized macular rash	Diffuse macular, maculopapular, or morbilliform rash OR Target lesions	Diffuse macular, maculopapular, or morbilliform rash with vesicles or limited number of bullae OR Superficial ulcerations of mucous membrane limited to one site	Extensive or generalized bullous lesions OR Stevens-Johnson syndrome OR Ulceration of mucous membrane involving two or more distinct mucosal sites OR Toxic epidermal necrolysis (TEN)
Hyperpigmentation	Slight or localized	Marked or generalized	NA	NA
Hypopigmentation	Slight or localized	Marked or generalized	NA	NA
Pruritis (itching – no skin lesions)<br>(See also Injection Site Reactions: Pruritis associated with injection)	Itching causing no or minimal interference with usual social & functional activities	Itching causing greater than minimal interference with usual social & functional activities	Itching causing inability to perform usual social & functional activities	NA
**CARDIOVASCULAR**				
Cardiac arrhythmia (general)<br>(By ECG or physical exam)	Asymptomatic AND No intervention indicated	Asymptomatic AND Non-urgent medical intervention indicated	Symptomatic, non-life-threatening AND Non-urgent medical intervention indicated	Life-threatening arrhythmia OR Urgent intervention indicated
Cardiac-ischemia/infarction	NA	NA	Symptomatic ischemia (stable angina) OR Testing consistent with ischemia	Unstable angina OR Acute myocardial infarction

Basic Self-care Functions – Adult: Activities such as bathing, dressing, toileting, transfer/movement, continence, and feeding.

Basic Self-care Functions – Young Children: Activities that are age and culturally appropriate (e.g., feeding self with culturally appropriate eating implement).

Usual Social & Functional Activities – Adult: Adaptive tasks and desirable activities, such as going to work, shopping, cooking, use of transportation, pursuing a hobby, etc.

Usual Social & Functional Activities – Young Children: Activities that are age and culturally appropriate (e.g., social interactions, play activities, learning tasks, etc.).

CLINICAL				
**PARAMETER**	**GRADE 1 MILD**	**GRADE 2 MODERATE**	**GRADE 3 SEVERE**	**GRADE 4 POTENTIALLY LIFE-THREATENING**
Hemorrhage (significant acute blood loss)	NA	Symptomatic AND No transfusion indicated	Symptomatic AND Transfusion of ≤ 2 units packed RBCs (for children ≤ 10 cc/kg) indicated	Life-threatening hypotension OR Transfusion of > 2 units packed RBCs (for children > 10 cc/kg) indicated
Hypertension				
**Adult > 17 years** (with repeat testing at same visit)	> 140 – 159 mmHg systolic OR > 90 – 99 mmHg diastolic	> 160 – 179 mmHg systolic OR > 100 – 109 mmHg diastolic	> 180 mmHg systolic OR > 110 mmHg diastolic	Life-threatening consequences (e.g., malignant hypertension) OR Hospitalization indicated (other than emergency room visit)
**Pediatric ≤ 17 years** (with repeat testing at same visit)	NA	91st – 94th percentile adjusted for age, height, and gender (systolic and/or diastolic)	≥ 95th percentile adjusted for age, height, and gender (systolic and/or diastolic)	Life-threatening consequences (e.g., malignant hypertension) OR Hospitalization indicated (other than emergency room visit)
Hypotension	NA	Symptomatic, corrected with oral fluid replacement	Symptomatic, IV fluids indicated	Shock requiring use of vasopressors or mechanical assistance to maintain blood pressure
Pericardial effusion	Asymptomatic, small effusion requiring no intervention	Asymptomatic, moderate or larger effusion requiring no intervention	Effusion with non-life threatening physiologic consequences OR Effusion with non-urgent intervention indicated	Life-threatening consequences (e.g., tamponade) OR Urgent intervention indicated
Prolonged PR interval				
**Adult > 16 years**	PR interval 0.21 – 0.25 sec	PR interval > 0.25 sec	Type II 2nd degree AV block OR Ventricular pause > 3.0 sec	Complete AV block
**Pediatric ≤ 16 years**	1st degree AV block (PR > normal for age and rate)	Type I 2nd degree AV block	Type II 2nd degree AV block	Complete AV block

Basic Self-care Functions – Adult: Activities such as bathing, dressing, toileting, transfer/movement, continence, and feeding.

Basic Self-care Functions – Young Children: Activities that are age and culturally appropriate (e.g., feeding self with culturally appropriate eating implement).

Usual Social & Functional Activities – Adult: Adaptive tasks and desirable activities, such as going to work, shopping, cooking, use of transportation, pursuing a hobby, etc.

Usual Social & Functional Activities – Young Children: Activities that are age and culturally appropriate (e.g., social interactions, play activities, learning tasks, etc.).

CLINICAL					
**PARAMETER**		**GRADE 1 MILD**	**GRADE 2 MODERATE**	**GRADE 3 SEVERE**	**GRADE 4 POTENTIALLY LIFE-THREATENING**
Prolonged QTc					
	**Adult > 16 years**	Asymptomatic, QTc interval 0.45 – 0.47 sec OR Increase interval < 0.03 sec above baseline	Asymptomatic, QTc interval 0.48 – 0.49 sec OR Increase in interval 0.03 – 0.05 sec above baseline	Asymptomatic, QTc interval ≥ 0.50 sec OR Increase in interval ≥ 0.06 sec above baseline	Life-threatening consequences, e.g. Torsade de pointes or other associated serious ventricular dysrhythmia
	**Pediatric ≤ 16 years**	Asymptomatic, QTc interval 0.450 – 0.464 sec	Asymptomatic, QTc interval 0.465 – 0.479 sec	Asymptomatic, QTc interval ≥ 0.480 sec	Life-threatening consequences, e.g. Torsade de pointes or other associated serious ventricular dysrhythmia
Thrombosis/embolism		NA	Deep vein thrombosis AND No intervention indicated (e.g., anticoagulation, lysis filter, invasive procedure)	Deep vein thrombosis AND Intervention indicated (e.g., anticoagulation, lysis filter, invasive procedure)	Embolic event (e.g., pulmonary embolism, life-threatening thrombus)
Vasovagal episode (associated with a procedure of any kind)		Present without loss of consciousness	Present with transient loss of consciousness	NA	NA
Ventricular dysfunction (congestive heart failure)		NA	Asymptomatic diagnostic finding AND intervention indicated	New onset with symptoms OR Worsening symptomatic congestive heart failure	Life-threatening congestive heart failure
**GASTROINTESTINAL**					
Anorexia		Loss of appetite without decreased oral intake	Loss of appetite associated with decreased oral intake without significant weight loss	Loss of appetite associated with significant weight loss	Life-threatening consequences OR Aggressive intervention indicated [e.g., tube feeding or total parenteral nutrition (TPN)]
Ascites		Asymptomatic	Symptomatic AND Intervention indicated (e.g., diuretics or therapeutic paracentesis)	Symptomatic despite intervention	Life-threatening consequences

Basic Self-care Functions – Adult: Activities such as bathing, dressing, toileting, transfer/movement, continence, and feeding.

Basic Self-care Functions – Young Children: Activities that are age and culturally appropriate (e.g., feeding self with culturally appropriate eating implement).

Usual Social & Functional Activities – Adult: Adaptive tasks and desirable activities, such as going to work, shopping, cooking, use of transportation, pursuing a hobby, etc.

Usual Social & Functional Activities – Young Children: Activities that are age and culturally appropriate (e.g., social interactions, play activities, learning tasks, etc.).

CLINICAL				
**PARAMETER**	**GRADE 1 MILD**	**GRADE 2 MODERATE**	**GRADE 3 SEVERE**	**GRADE 4 POTENTIALLY LIFE-THREATENING**
Cholecystitis	NA	Symptomatic AND Medical intervention indicated	Radiologic, endoscopic, or operative intervention indicated	Life-threatening consequences (e.g., sepsis or perforation)
Constipation	NA	Persistent constipation requiring regular use of dietary modifications, laxatives, or enemas	Obstipation with manual evacuation indicated	Life-threatening consequences (e.g., obstruction)
Diarrhea				
**Adult and Pediatric ≥ 1 year**	Transient or intermittent episodes of unformed stools OR Increase of ≤ 3 stools over baseline per 24-hour period	Persistent episodes of unformed to watery stools OR Increase of 4 – 6 stools over baseline per 24-hour period	Bloody diarrhea OR Increase of ≥ 7 stools per 24-hour period OR IV fluid replacement indicated	Life-threatening consequences (e.g., hypotensive shock)
**Pediatric < 1 year**	Liquid stools (more unformed than usual) but usual number of stools	Liquid stools with increased number of stools OR Mild dehydration	Liquid stools with moderate dehydration	Liquid stools resulting in severe dehydration with aggressive rehydration indicated OR Hypotensive shock
Dysphagia-Odynophagia	Symptomatic but able to eat usual diet	Symptoms causing altered dietary intake without medical intervention indicated	Symptoms causing severely altered dietary intake with medical intervention indicated	Life-threatening reduction in oral intake
Mucositis/stomatitis (clinical exam)<br>Indicate site (e.g., larynx, oral)<br>See Genitourinary for Vulvovaginitis<br>See also Dysphagia-Odynophagia and Proctitis	Erythema of the mucosa	Patchy pseudomembranes or ulcerations	Confluent pseudomembranes or ulcerations OR Mucosal bleeding with minor trauma	Tissue necrosis OR Diffuse spontaneous mucosal bleeding OR Life-threatening consequences (e.g., aspiration, choking)
Nausea	Transient (< 24 hours) or intermittent nausea with no or minimal interference with oral intake	Persistent nausea resulting in decreased oral intake for 24 – 48 hours	Persistent nausea resulting in minimal oral intake for > 48 hours OR Aggressive rehydration indicated (e.g., IV fluids)	Life-threatening consequences (e.g., hypotensive shock)

Basic Self-care Functions – Adult: Activities such as bathing, dressing, toileting, transfer/movement, continence, and feeding.

Basic Self-care Functions – Young Children: Activities that are age and culturally appropriate (e.g., feeding self with culturally appropriate eating implement).

Usual Social & Functional Activities – Adult: Adaptive tasks and desirable activities, such as going to work, shopping, cooking, use of transportation, pursuing a hobby, etc.

Usual Social & Functional Activities – Young Children: Activities that are age and culturally appropriate (e.g., social interactions, play activities, learning tasks, etc.).

CLINICAL				
**PARAMETER**	**GRADE 1 MILD**	**GRADE 2 MODERATE**	**GRADE 3 SEVERE**	**GRADE 4 POTENTIALLY LIFE-THREATENING**
Pancreatitis	NA	Symptomatic AND Hospitalization not indicated (other than emergency room visit)	Symptomatic AND Hospitalization indicated (other than emergency room visit)	Life-threatening consequences (e.g., circulatory failure, hemorrhage, sepsis)
Proctitis (functional-symptomatic)<br>Also see Mucositis/stomatitis for clinical exam	Rectal discomfort AND No intervention indicated	Symptoms causing greater than minimal interference with usual social & functional activities OR Medical intervention indicated	Symptoms causing inability to perform usual social & functional activities OR Operative intervention indicated	Life-threatening consequences (e.g., perforation)
Vomiting	Transient or intermittent vomiting with no or minimal interference with oral intake	Frequent episodes of vomiting with no or mild dehydration	Persistent vomiting resulting in orthostatic hypotension OR Aggressive rehydration indicated (e.g., IV fluids)	Life-threatening consequences (e.g., hypotensive shock)
**NEUROLOGIC**				
Alteration in personality-behavior or in mood (e.g., agitation, anxiety, depression, mania, psychosis)	Alteration causing no or minimal interference with usual social & functional activities	Alteration causing greater than minimal interference with usual social & functional activities	Alteration causing inability to perform usual social & functional activities	Behavior potentially harmful to self or others (e.g., suicidal and homicidal ideation or attempt, acute psychosis) OR Causing inability to perform basic self-care functions
Altered Mental Status<br>For Dementia, see Cognitive and behavioral/attentional disturbance (including dementia and attention deficit disorder)	Changes causing no or minimal interference with usual social & functional activities	Mild lethargy or somnolence causing greater than minimal interference with usual social & functional activities	Confusion, memory impairment, lethargy, or somnolence causing inability to perform usual social & functional activities	Delirium OR obtundation, OR coma
Ataxia	Asymptomatic ataxia detectable on exam OR Minimal ataxia causing no or minimal interference with usual social & functional activities	Symptomatic ataxia causing greater than minimal interference with usual social & functional activities	Symptomatic ataxia causing inability to perform usual social & functional activities	Disabling ataxia causing inability to perform basic self-care functions

Basic Self-care Functions – Adult: Activities such as bathing, dressing, toileting, transfer/movement, continence, and feeding.

Basic Self-care Functions – Young Children: Activities that are age and culturally appropriate (e.g., feeding self with culturally appropriate eating implement).

Usual Social & Functional Activities – Adult: Adaptive tasks and desirable activities, such as going to work, shopping, cooking, use of transportation, pursuing a hobby, etc.

Usual Social & Functional Activities – Young Children: Activities that are age and culturally appropriate (e.g., social interactions, play activities, learning tasks, etc.).

CLINICAL				
**PARAMETER**	**GRADE 1 MILD**	**GRADE 2 MODERATE**	**GRADE 3 SEVERE**	**GRADE 4 POTENTIALLY LIFE-THREATENING**
Cognitive and behavioral/attentional disturbance (including dementia and attention deficit disorder)	Disability causing no or minimal interference with usual social & functional activities OR Specialized resources not indicated	Disability causing greater than minimal interference with usual social & functional activities OR Specialized resources on part-time basis indicated	Disability causing inability to perform usual social & functional activities OR Specialized resources on a full-time basis indicated	Disability causing inability to perform basic self-care functions OR Institutionalization indicated
CNS ischemia (acute)	NA	NA	Transient ischemic attack	Cerebral vascular accident (CVA, stroke) with neurological deficit
Developmental delay – **Pediatric ≤ 16 years**	Mild developmental delay, either motor or cognitive, as determined by comparison with a developmental screening tool appropriate for the setting	Moderate developmental delay, either motor or cognitive, as determined by comparison with a developmental screening tool appropriate for the setting	Severe developmental delay, either motor or cognitive, as determined by comparison with a developmental screening tool appropriate for the setting	Developmental regression, either motor or cognitive, as determined by comparison with a developmental screening tool appropriate for the setting
Headache	Symptoms causing no or minimal interference with usual social & functional activities	Symptoms causing greater than minimal interference with usual social & functional activities	Symptoms causing inability to perform usual social & functional activities	Symptoms causing inability to perform basic self-care functions OR Hospitalization indicated (other than emergency room visit) OR Headache with significant impairment of alertness or other neurologic function
Insomnia	NA	Difficulty sleeping causing greater than minimal interference with usual social & functional activities	Difficulty sleeping causing inability to perform usual social & functional activities	Disabling insomnia causing inability to perform basic self-care functions
Neuromuscular weakness (including myopathy & neuropathy)	Asymptomatic with decreased strength on exam OR Minimal muscle weakness causing no or minimal interference with usual social & functional activities	Muscle weakness causing greater than minimal interference with usual social & functional activities	Muscle weakness causing inability to perform usual social & functional activities	Disabling muscle weakness causing inability to perform basic self-care functions OR Respiratory muscle weakness impairing ventilation

Basic Self-care Functions – Adult: Activities such as bathing, dressing, toileting, transfer/movement, continence, and feeding.

Basic Self-care Functions – Young Children: Activities that are age and culturally appropriate (e.g., feeding self with culturally appropriate eating implement).

Usual Social & Functional Activities – Adult: Adaptive tasks and desirable activities, such as going to work, shopping, cooking, use of transportation, pursuing a hobby, etc.

Usual Social & Functional Activities – Young Children: Activities that are age and culturally appropriate (e.g., social interactions, play activities, learning tasks, etc.).

CLINICAL				
**PARAMETER**	**GRADE 1 MILD**	**GRADE 2 MODERATE**	**GRADE 3 SEVERE**	**GRADE 4 POTENTIALLY LIFE-THREATENING**
Neurosensory alteration (including paresthesia and painful neuropathy)	Asymptomatic with sensory alteration on exam or minimal paresthesia causing no or minimal interference with usual social & functional activities	Sensory alteration or paresthesia causing greater than minimal interference with usual social & functional activities	Sensory alteration or paresthesia causing inability to perform usual social & functional activities	Disabling sensory alteration or paresthesia causing inability to perform basic self-care functions
Seizure: (new onset) **– Adult ≥ 18 years**<br>See also Seizure: (known pre-existing seizure disorder)	NA	1 seizure	2 – 4 seizures	Seizures of any kind which are prolonged, repetitive (e.g., status epilepticus), or difficult to control (e.g., refractory epilepsy)
Seizure: (known pre-existing seizure disorder) **– Adult ≥ 18 years**<br>For worsening of existing epilepsy the grades should be based on an increase from previous level of control to any of these levels.	NA	Increased frequency of pre-existing seizures (non-repetitive) without change in seizure character OR Infrequent break-through seizures while on stable medication in a previously controlled seizure disorder	Change in seizure character from baseline either in duration or quality (e.g., severity or focality)	Seizures of any kind which are prolonged, repetitive (e.g., status epilepticus), or difficult to control (e.g., refractory epilepsy)
Seizure **– Pediatric < 18 years**	Seizure, generalized onset with or without secondary generalization, lasting < 5 minutes with < 24 hours post ictal state	Seizure, generalized onset with or without secondary generalization, lasting 5 – 20 minutes with < 24 hours post ictal state	Seizure, generalized onset with or without secondary generalization, lasting > 20 minutes	Seizure, generalized onset with or without secondary generalization, requiring intubation and sedation
Syncope (not associated with a procedure)	NA	Present	NA	NA
Vertigo	Vertigo causing no or minimal interference with usual social & functional activities	Vertigo causing greater than minimal interference with usual social & functional activities	Vertigo causing inability to perform usual social & functional activities	Disabling vertigo causing inability to perform basic self-care functions

Basic Self-care Functions – Adult: Activities such as bathing, dressing, toileting, transfer/movement, continence, and feeding.

Basic Self-care Functions – Young Children: Activities that are age and culturally appropriate (e.g., feeding self with culturally appropriate eating implement).

Usual Social & Functional Activities – Adult: Adaptive tasks and desirable activities, such as going to work, shopping, cooking, use of transportation, pursuing a hobby, etc.

Usual Social & Functional Activities – Young Children: Activities that are age and culturally appropriate (e.g., social interactions, play activities, learning tasks, etc.).

CLINICAL				
**PARAMETER**	**GRADE 1 MILD**	**GRADE 2 MODERATE**	**GRADE 3 SEVERE**	**GRADE 4 POTENTIALLY LIFE-THREATENING**
**RESPIRATORY**				
Bronchospasm (acute)	FEV1 or peak flow reduced to 70 – 80%	FEV1 or peak flow 50 – 69%	FEV1 or peak flow 25 – 49%	Cyanosis OR FEV1 or peak flow < 25% OR Intubation
Dyspnea or respiratory distress				
**Adult ≥ 14 years**	Dyspnea on exertion with no or minimal interference with usual social & functional activities	Dyspnea on exertion causing greater than minimal interference with usual social & functional activities	Dyspnea at rest causing inability to perform usual social & functional activities	Respiratory failure with ventilatory support indicated
**Pediatric < 14 years**	Wheezing OR minimal increase in respiratory rate for age	Nasal flaring OR Intercostal retractions OR Pulse oximetry 90 – 95%	Dyspnea at rest causing inability to perform usual social & functional activities OR Pulse oximetry < 90%	Respiratory failure with ventilatory support indicated
**MUSCULOSKELETAL**				
Arthralgia<br>See also Arthritis	Joint pain causing no or minimal interference with usual social & functional activities	Joint pain causing greater than minimal interference with usual social & functional activities	Joint pain causing inability to perform usual social & functional activities	Disabling joint pain causing inability to perform basic self-care functions
Arthritis<br>See also Arthralgia	Stiffness or joint swelling causing no or minimal interference with usual social & functional activities	Stiffness or joint swelling causing greater than minimal interference with usual social & functional activities	Stiffness or joint swelling causing inability to perform usual social & functional activities	Disabling joint stiffness or swelling causing inability to perform basic self-care functions
Bone Mineral Loss				
**Adult ≥ 21 years**	BMD t-score -2.5 to -1.0	BMD t-score < -2.5	Pathological fracture (including loss of vertebral height)	Pathologic fracture causing life-threatening consequences
**Pediatric < 21 years**	BMD z-score -2.5 to -1.0	BMD z-score < -2.5	Pathological fracture (including loss of vertebral height)	Pathologic fracture causing life-threatening consequences

Basic Self-care Functions – Adult: Activities such as bathing, dressing, toileting, transfer/movement, continence, and feeding.

Basic Self-care Functions – Young Children: Activities that are age and culturally appropriate (e.g., feeding self with culturally appropriate eating implement).

Usual Social & Functional Activities – Adult: Adaptive tasks and desirable activities, such as going to work, shopping, cooking, use of transportation, pursuing a hobby, etc.

Usual Social & Functional Activities – Young Children: Activities that are age and culturally appropriate (e.g., social interactions, play activities, learning tasks, etc.).

CLINICAL				
**PARAMETER**	**GRADE 1 MILD**	**GRADE 2 MODERATE**	**GRADE 3 SEVERE**	**GRADE 4 POTENTIALLY LIFE-THREATENING**
Myalgia (non-injection site)	Muscle pain causing no or minimal interference with usual social & functional activities	Muscle pain causing greater than minimal interference with usual social & functional activities	Muscle pain causing inability to perform usual social & functional activities	Disabling muscle pain causing inability to perform basic self-care functions
Osteonecrosis	NA	Asymptomatic with radiographic findings AND No operative intervention indicated	Symptomatic bone pain with radiographic findings OR Operative intervention indicated	Disabling bone pain with radiographic findings causing inability to perform basic self-care functions
**GENITOURINARY**				
Cervicitis (symptoms) (For use in studies evaluating topical study agents) For other cervicitis see Infection: Infection (any other than HIV infection)	Symptoms causing no or minimal interference with usual social & functional activities	Symptoms causing greater than minimal interference with usual social & functional activities	Symptoms causing inability to perform usual social & functional activities	Symptoms causing inability to perform basic self-care functions
Cervicitis (clinical exam) (For use in studies evaluating topical study agents) For other cervicitis see Infection: Infection (any other than HIV infection)	Minimal cervical abnormalities on examination (erythema, mucopurulent discharge, or friability) OR Epithelial disruption < 25% of total surface	Moderate cervical abnormalities on examination (erythema, mucopurulent discharge, or friability) OR Epithelial disruption of 25 – 49% total surface	Severe cervical abnormalities on examination (erythema, mucopurulent discharge, or friability) OR Epithelial disruption 50 – 75% total surface	Epithelial disruption > 75% total surface
Inter-menstrual bleeding (IMB)	Spotting observed by participant OR Minimal blood observed during clinical or colposcopic examination	Inter-menstrual bleeding not greater in duration or amount than usual menstrual cycle	Inter-menstrual bleeding greater in duration or amount than usual menstrual cycle	Hemorrhage with life-threatening hypotension OR Operative intervention indicated
Urinary tract obstruction (e.g., stone)	NA	Signs or symptoms of urinary tract obstruction without hydronephrosis or renal dysfunction	Signs or symptoms of urinary tract obstruction with hydronephrosis or renal dysfunction	Obstruction causing life-threatening consequences

Basic Self-care Functions – Adult: Activities such as bathing, dressing, toileting, transfer/movement, continence, and feeding.

Basic Self-care Functions – Young Children: Activities that are age and culturally appropriate (e.g., feeding self with culturally appropriate eating implement).

Usual Social & Functional Activities – Adult: Adaptive tasks and desirable activities, such as going to work, shopping, cooking, use of transportation, pursuing a hobby, etc.

Usual Social & Functional Activities – Young Children: Activities that are age and culturally appropriate (e.g., social interactions, play activities, learning tasks, etc.).

CLINICAL				
**PARAMETER**	**GRADE 1 MILD**	**GRADE 2 MODERATE**	**GRADE 3 SEVERE**	**GRADE 4 POTENTIALLY LIFE-THREATENING**
Vulvovaginitis (symptoms)<br>(Use in studies evaluating topical study agents)<br>For other vulvovaginitis see Infection: Infection (any other than HIV infection)	Symptoms causing no or minimal interference with usual social & functional activities	Symptoms causing greater than minimal interference with usual social & functional activities	Symptoms causing inability to perform usual social & functional activities	Symptoms causing inability to perform basic self-care functions
Vulvovaginitis (clinical exam)<br>(Use in studies evaluating topical study agents)<br>For other vulvovaginitis see Infection: Infection (any other than HIV infection)	Minimal vaginal abnormalities on examination OR Epithelial disruption < 25% of total surface	Moderate vaginal abnormalities on examination OR Epithelial disruption of 25 - 49% total surface	Severe vaginal abnormalities on examination OR Epithelial disruption 50 - 75% total surface	Vaginal perforation OR Epithelial disruption > 75% total surface
**OCULAR/VISUAL**				
Uveitis	Asymptomatic but detectable on exam	Symptomatic anterior uveitis OR Medical intervention indicated	Posterior or pan-uveitis OR Operative intervention indicated	Disabling visual loss in affected eye(s)
Visual changes (from baseline)	Visual changes causing no or minimal interference with usual social & functional activities	Visual changes causing greater than minimal interference with usual social & functional activities	Visual changes causing inability to perform usual social & functional activities	Disabling visual loss in affected eye(s)
**ENDOCRINE/METABOLIC**				
Abnormal fat accumulation (e.g., back of neck, breasts, abdomen)	Detectable by study participant (or by caregiver for young children and disabled adults)	Detectable on physical exam by health care provider	Disfiguring OR Obvious changes on casual visual inspection	NA

Basic Self-care Functions – Adult: Activities such as bathing, dressing, toileting, transfer/movement, continence, and feeding.

Basic Self-care Functions – Young Children: Activities that are age and culturally appropriate (e.g., feeding self with culturally appropriate eating implement).

Usual Social & Functional Activities – Adult: Adaptive tasks and desirable activities, such as going to work, shopping, cooking, use of transportation, pursuing a hobby, etc.

Usual Social & Functional Activities – Young Children: Activities that are age and culturally appropriate (e.g., social interactions, play activities, learning tasks, etc.).

CLINICAL				
**PARAMETER**	**GRADE 1 MILD**	**GRADE 2 MODERATE**	**GRADE 3 SEVERE**	**GRADE 4 POTENTIALLY LIFE-THREATENING**
Diabetes mellitus	NA	New onset without need to initiate medication OR Modification of current medications to regain glucose control	New onset with initiation of medication indicated OR Diabetes uncontrolled despite treatment modification	Life-threatening consequences (e.g., ketoacidosis, hyperosmolar non-ketotic coma)
Gynecomastia	Detectable by study participant or caregiver (for young children and disabled adults)	Detectable on physical exam by health care provider	Disfiguring OR Obvious on casual visual inspection	NA
Hyperthyroidism	Asymptomatic	Symptomatic causing greater than minimal interference with usual social & functional activities OR Thyroid suppression therapy indicated	Symptoms causing inability to perform usual social & functional activities OR Uncontrolled despite treatment modification	Life-threatening consequences (e.g., thyroid storm)
Hypothyroidism	Asymptomatic	Symptomatic causing greater than minimal interference with usual social & functional activities OR Thyroid replacement therapy indicated	Symptoms causing inability to perform usual social & functional activities OR Uncontrolled despite treatment modification	Life-threatening consequences (e.g., myxedema coma)
Lipoatrophy (e.g., fat loss from the face, extremities, buttocks)	Detectable by study participant (or by caregiver for young children and disabled adults)	Detectable on physical exam by health care provider	Disfiguring OR Obvious on casual visual inspection	NA

Basic Self-care Functions – Adult: Activities such as bathing, dressing, toileting, transfer/movement, continence, and feeding.

Basic Self-care Functions – Young Children: Activities that are age and culturally appropriate (e.g., feeding self with culturally appropriate eating implement).

Usual Social & Functional Activities – Adult: Adaptive tasks and desirable activities, such as going to work, shopping, cooking, use of transportation, pursuing a hobby, etc.

Usual Social & Functional Activities – Young Children: Activities that are age and culturally appropriate (e.g., social interactions, play activities, learning tasks, etc.).

LABORATORY				
**PARAMETER**	**GRADE 1 MILD**	**GRADE 2 MODERATE**	**GRADE 3 SEVERE**	**GRADE 4 POTENTIALLY LIFE-THREATENING**
**HEMATOLOGY**	***Standard International Units are listed in italics***			
Absolute CD4+ count **– Adult and Pediatric > 13 years** (HIV NEGATIVE ONLY)	300 – 400/mm^3 *300 – 400/µL*	200 – 299/mm^3 *200 – 299/µL*	100 – 199/mm^3 *100 – 199/µL*	< 100/mm^3 *< 100/µL*
Absolute lymphocyte count **– Adult and Pediatric > 13 years** (HIV NEGATIVE ONLY)	600 – 650/mm^3 *0.600 x 10^9 – 0.650 x 10^9/L*	500 – 599/mm^3 *0.500 x 10^9 – 0.599 x 10^9/L*	350 – 499/mm^3 *0.350 x 10^9 – 0.499 x 10^9/L*	< 350/mm^3 *< 0.350 x 10^9/L*
Absolute neutrophil count (ANC)				
**Adult and Pediatric, > 7 days**	1,000 – 1,300/mm^3 *1.000 x 10^9 – 1.300 x 10^9/L*	750 – 999/mm^3 *0.750 x 10^9 – 0.999 x 10^9/L*	500 – 749/mm^3 *0.500 x 10^9 – 0.749 x 10^9/L*	< 500/mm^3 *< 0.500 x 10^9/L*
**Infant*†, 2 – ≤ 7 days**	1,250 – 1,500/mm^3 *1.250 x 10^9 – 1.500 x 10^9/L*	1,000 – 1,249/mm^3 *1.000 x 10^9 – 1.249 x 10^9/L*	750 – 999/mm^3 *0.750 x 10^9 – 0.999 x 10^9/L*	< 750/mm^3 *< 0.750 x 10^9/L*
**Infant*†, 1 day**	4,000 – 5,000/mm^3 *4.000 x 10^9 – 5.000 x 10^9/L*	3,000 – 3,999/mm^3 *3.000 x 10^9 – 3.999 x10^9/L*	1,500 – 2,999/mm^3 *1.500 x 10^9 – 2.999 x 10^9/L*	< 1,500/mm^3 *< 1.500 x 10^9/L*
Fibrinogen, decreased	100 – 200 mg/dL *1.00 – 2.00 g/L* OR 0.75 – 0.99 x LLN	75 – 99 mg/dL *0.75 – 0.99 g/L* OR 0.50 – 0.74 x LLN	50 – 74 mg/dL *0.50 – 0.74 g/L* OR 0.25 – 0.49 x LLN	< 50 mg/dL *< 0.50 g/L* OR < 0.25 x LLN OR Associated with gross bleeding
Hemoglobin (Hgb)				
**Adult and Pediatric ≥ 57 days** (HIV POSITIVE ONLY)	8.5 – 10.0 g/dL *1.32 – 1.55 mmol/L*	7.5 – 8.4 g/dL *1.16 – 1.31 mmol/L*	6.50 – 7.4 g/dL *1.01 – 1.15 mmol/L*	< 6.5 g/dL *< 1.01 mmol/L*
**Adult and Pediatric ≥ 57 days** (HIV NEGATIVE ONLY)	10.0 – 10.9 g/dL *1.55 – 1.69 mmol/L* OR Any decrease 2.5 – 3.4 g/dL *0.39 – 0.53 mmol/L*	9.0 – 9.9 g/dL *1.40 – 1.54 mmol/L* OR Any decrease 3.5 – 4.4 g/dL *0.54 – 0.68 mmol/L*	7.0 – 8.9 g/dL *1.09 – 1.39 mmol/L* OR Any decrease ≥ 4.5 g/dL *≥ 0.69 mmol/L*	< 7.0 g/dL *< 1.09 mmol/L*
**Infant*†, 36 – 56 days** (HIV POSITIVE OR NEGATIVE)	8.5 – 9.4 g/dL *1.32 – 1.46 mmol/L*	7.0 – 8.4 g/dL *1.09 – 1.31 mmol/L*	6.0 – 6.9 g/dL *0.93 – 1.08 mmol/L*	< 6.00 g/dL *< 0.93 mmol/L*

* Values are for term infants.

† Use age and sex appropriate values (e.g., bilirubin), including preterm infants.

LABORATORY				
**PARAMETER**	**GRADE 1 MILD**	**GRADE 2 MODERATE**	**GRADE 3 SEVERE**	**GRADE 4 POTENTIALLY LIFE-THREATENING**
**Infant*,†, 22 – 35 days** (HIV POSITIVE OR NEGATIVE)	9.5 – 10.5 g/dL<br>*1.47 – 1.63 mmol/L*	8.0 – 9.4 g/dL<br>*1.24 – 1.46 mmol/L*	7.0 – 7.9 g/dL<br>*1.09 – 1.23 mmol/L*	< 7.00 g/dL<br>*< 1.09 mmol/L*
**Infant*,†, 1 – 21 days** (HIV POSITIVE OR NEGATIVE)	12.0 – 13.0 g/dL<br>*1.86 – 2.02 mmol/L*	10.0 – 11.9 g/dL<br>*1.55 – 1.85 mmol/L*	9.0 – 9.9 g/dL<br>*1.40 – 1.54 mmol/L*	< 9.0 g/dL<br>*< 1.40 mmol/L*
International Normalized Ratio of prothrombin time (INR)	1.1 – 1.5 x ULN	1.6 – 2.0 x ULN	2.1 – 3.0 x ULN	> 3.0 x ULN
Methemoglobin	5.0 – 10.0%	10.1 – 15.0%	15.1 – 20.0%	> 20.0%
Prothrombin Time (PT)	1.1 – 1.25 x ULN	1.26 – 1.50 x ULN	1.51 – 3.00 x ULN	> 3.00 x ULN
Partial Thromboplastin Time (PTT)	1.1 – 1.66 x ULN	1.67 – 2.33 x ULN	2.34 – 3.00 x ULN	> 3.00 x ULN
Platelets, decreased	100,000 – 124,999/mm^3<br>*100.000 x 10^9 – 124.999 x 10^9/L*	50,000 – 99,999/mm^3<br>*50.000 x 10^9 – 99.999 x 10^9/L*	25,000 – 49,999/mm^3<br>*25.000 x 10^9 – 49.999 x 10^9/L*	< 25,000/mm^3<br>*< 25.000 x 10^9/L*
WBC, decreased	2,000 – 2,500/mm^3<br>*2.000 x 10^9 – 2.500 x 10^9/L*	1,500 – 1,999/mm^3<br>*1.500 x 10^9 – 1.999 x 10^9/L*	1,000 – 1,499/mm^3<br>*1.000 x 10^9 – 1.499 x 10^9/L*	< 1,000/mm^3<br>*< 1.000 x 10^9/L*
**CHEMISTRIES**	***Standard International Units are listed in italics***			
Acidosis	NA	pH < normal, but ≥ 7.3	pH < 7.3 without life-threatening consequences	pH < 7.3 with life-threatening consequences
Albumin, serum, low	3.0 g/dL – < LLN<br>*30 g/L – < LLN*	2.0 – 2.9 g/dL<br>*20 – 29 g/L*	< 2.0 g/dL<br>*< 20 g/L*	NA
Alkaline Phosphatase	1.25 – 2.5 x ULN†	2.6 – 5.0 x ULN†	5.1 – 10.0 x ULN†	> 10.0 x ULN†
Alkalosis	NA	pH > normal, but ≤ 7.5	pH > 7.5 without life-threatening consequences	pH > 7.5 with life-threatening consequences
ALT (SGPT)	1.25 – 2.5 x ULN	2.6 – 5.0 x ULN	5.1 – 10.0 x ULN	> 10.0 x ULN
AST (SGOT)	1.25 – 2.5 x ULN	2.6 – 5.0 x ULN	5.1 – 10.0 x ULN	> 10.0 x ULN
Bicarbonate, serum, low	16.0 mEq/L – < LLN<br>*16.0 mmol/L – < LLN*	11.0 – 15.9 mEq/L<br>*11.0 – 15.9 mmol/L*	8.0 – 10.9 mEq/L<br>*8.0 – 10.9 mmol/L*	< 8.0 mEq/L<br>*< 8.0 mmol/L*
Bilirubin (Total)				
**Adult and Pediatric > 14 days**	1.1 – 1.5 x ULN	1.6 – 2.5 x ULN	2.6 – 5.0 x ULN	> 5.0 x ULN

* Values are for term infants.

† Use age and sex appropriate values (e.g., bilirubin), including preterm infants.

LABORATORY					
**PARAMETER**		**GRADE 1 MILD**	**GRADE 2 MODERATE**	**GRADE 3 SEVERE**	**GRADE 4 POTENTIALLY LIFE-THREATENING**
	**Infant*†, ≤ 14 days** (non-hemolytic)	NA	20.0 – 25.0 mg/dL<br>*342 – 428 µmol/L*	25.1 – 30.0 mg/dL<br>*429 – 513 µmol/L*	> 30.0 mg/dL<br>*> 513.0 µmol/L*
	**Infant*†, ≤ 14 days** (hemolytic)	NA	NA	20.0 – 25.0 mg/dL<br>*342 – 428 µmol/L*	> 25.0 mg/dL<br>*> 428 µmol/L*
Calcium, serum, high (corrected for albumin)					
	**Adult and Pediatric ≥ 7 days**	10.6 – 11.5 mg/dL<br>*2.65 – 2.88 mmol/L*	11.6 – 12.5 mg/dL<br>*2.89 – 3.13 mmol/L*	12.6 – 13.5 mg/dL<br>*3.14 – 3.38 mmol/L*	> 13.5 mg/dL<br>*> 3.38 mmol/L*
	**Infant*†, < 7 days**	11.5 – 12.4 mg/dL<br>*2.88 – 3.10 mmol/L*	12.5 – 12.9 mg/dL<br>*3.11 – 3.23 mmol/L*	13.0 – 13.5 mg/dL<br>*3.245 – 3.38 mmol/L*	> 13.5 mg/dL<br>*> 3.38 mmol/L*
Calcium, serum, low (corrected for albumin)					
	**Adult and Pediatric ≥ 7 days**	7.8 – 8.4 mg/dL<br>*1.95 – 2.10 mmol/L*	7.0 – 7.7 mg/dL<br>*1.75 – 1.94 mmol/L*	6.1 – 6.9 mg/dL<br>*1.53 – 1.74 mmol/L*	< 6.1 mg/dL<br>*< 1.53 mmol/L*
	**Infant*†, < 7 days**	6.5 – 7.5 mg/dL<br>*1.63 – 1.88 mmol/L*	6.0 – 6.4 mg/dL<br>*1.50 – 1.62 mmol/L*	5.50 – 5.90 mg/dL<br>*1.38 – 1.51 mmol/L*	< 5.50 mg/dL<br>*< 1.38 mmol/L*
Cardiac troponin I (cTnI)		NA	NA	NA	Levels consistent with myocardial infarction or unstable angina as defined by the manufacturer
Cardiac troponin T (cTnT)		NA	NA	NA	≥ 0.20 ng/mL<br>OR<br>Levels consistent with myocardial infarction or unstable angina as defined by the manufacturer
Cholesterol (fasting)					
	**Adult ≥ 18 years**	200 – 239 mg/dL<br>*5.18 – 6.19 mmol/L*	240 – 300 mg/dL<br>*6.20 – 7.77 mmol/L*	> 300 mg/dL<br>*> 7.77 mmol/L*	NA
	**Pediatric < 18 years**	170 – 199 mg/dL<br>*4.40 – 5.15 mmol/L*	200 – 300 mg/dL<br>*5.16 – 7.77 mmol/L*	> 300 mg/dL<br>*> 7.77 mmol/L*	NA
Creatine Kinase		3.0 – 5.9 x ULN†	6.0 – 9.9 x ULN†	10.0 – 19.9 x ULN†	≥ 20.0 x ULN†
Creatinine		1.1 – 1.3 x ULN†	1.4 – 1.8 x ULN†	1.9 – 3.4 x ULN†	≥ 3.5 x ULN†
Glucose, serum, high					
	Nonfasting	116 – 160 mg/dL<br>*6.44 – 8.88 mmol/L*	161 – 250 mg/dL<br>*8.89 – 13.88 mmol/L*	251 – 500 mg/dL<br>*13.89 – 27.75 mmol/L*	> 500 mg/dL<br>*> 27.75 mmol/L*
	Fasting	110 – 125 mg/dL<br>*6.11 – 6.94 mmol/L*	126 – 250 mg/dL<br>*6.95 – 13.88 mmol/L*	251 – 500 mg/dL<br>*13.89 – 27.75 mmol/L*	> 500 mg/dL<br>*> 27.75 mmol/L*

* Values are for term infants.

† Use age and sex appropriate values (e.g., bilirubin), including preterm infants.

LABORATORY				
**PARAMETER**	**GRADE 1 MILD**	**GRADE 2 MODERATE**	**GRADE 3 SEVERE**	**GRADE 4 POTENTIALLY LIFE-THREATENING**
Glucose, serum, low				
**Adult and Pediatric ≥ 1 month**	55 – 64 mg/dL<br>*3.05 – 3.55 mmol/L*	40 – 54 mg/dL<br>*2.22 – 3.06 mmol/L*	30 – 39 mg/dL<br>*1.67 – 2.23 mmol/L*	< 30 mg/dL<br>*< 1.67 mmol/L*
**Infant*[†], < 1 month**	50 – 54 mg/dL<br>*2.78 – 3.00 mmol/L*	40 – 49 mg/dL<br>*2.22 – 2.77 mmol/L*	30 – 39 mg/dL<br>*1.67 – 2.21 mmol/L*	< 30 mg/dL<br>*< 1.67 mmol/L*
Lactate	< 2.0 x ULN without acidosis	≥ 2.0 x ULN without acidosis	Increased lactate with pH < 7.3 without life-threatening consequences	Increased lactate with pH < 7.3 with life-threatening consequences
LDL cholesterol (fasting)				
**Adult ≥ 18 years**	130 – 159 mg/dL<br>*3.37 – 4.12 mmol/L*	160 – 190 mg/dL<br>*4.13 – 4.90 mmol/L*	≥ 190 mg/dL<br>*≥ 4.91 mmol/L*	NA
**Pediatric > 2 - < 18 years**	110 – 129 mg/dL<br>*2.85 – 3.34 mmol/L*	130 – 189 mg/dL<br>*3.35 – 4.90 mmol/L*	≥ 190 mg/dL<br>*≥ 4.91 mmol/L*	NA
Lipase	1.1 – 1.5 x ULN	1.6 – 3.0 x ULN	3.1 – 5.0 x ULN	> 5.0 x ULN
Magnesium, serum, low	1.2 – 1.4 mEq/L<br>*0.60 – 0.70 mmol/L*	0.9 – 1.1 mEq/L<br>*0.45 – 0.59 mmol/L*	0.6 – 0.8 mEq/L<br>*0.30 – 0.44 mmol/L*	< 0.60 mEq/L<br>*< 0.30 mmol/L*
Pancreatic amylase	1.1 – 1.5 x ULN	1.6 – 2.0 x ULN	2.1 – 5.0 x ULN	> 5.0 x ULN
Phosphate, serum, low				
**Adult and Pediatric > 14 years**	2.5 mg/dL – < LLN<br>*0.81 mmol/L – < LLN*	2.0 – 2.4 mg/dL<br>*0.65 – 0.80 mmol/L*	1.0 – 1.9 mg/dL<br>*0.32 – 0.64 mmol/L*	< 1.00 mg/dL<br>*< 0.32 mmol/L*
**Pediatric 1 year – 14 years**	3.0 – 3.5 mg/dL<br>*0.97 – 1.13 mmol/L*	2.5 – 2.9 mg/dL<br>*0.81 – 0.96 mmol/L*	1.5 – 2.4 mg/dL<br>*0.48 – 0.80 mmol/L*	< 1.50 mg/dL<br>*< 0.48 mmol/L*
**Pediatric < 1 year**	3.5 – 4.5 mg/dL<br>*1.13 – 1.45 mmol/L*	2.5 – 3.4 mg/dL<br>*0.81 – 1.12 mmol/L*	1.5 – 2.4 mg/dL<br>*0.48 – 0.80 mmol/L*	< 1.50 mg/dL<br>*< 0.48 mmol/L*
Potassium, serum, high	5.6 – 6.0 mEq/L<br>*5.6 – 6.0 mmol/L*	6.1 – 6.5 mEq/L<br>*6.1 – 6.5 mmol/L*	6.6 – 7.0 mEq/L<br>*6.6 – 7.0 mmol/L*	> 7.0 mEq/L<br>*> 7.0 mmol/L*
Potassium, serum, low	3.0 – 3.4 mEq/L<br>*3.0 – 3.4 mmol/L*	2.5 – 2.9 mEq/L<br>*2.5 – 2.9 mmol/L*	2.0 – 2.4 mEq/L<br>*2.0 – 2.4 mmol/L*	< 2.0 mEq/L<br>*< 2.0 mmol/L*
Sodium, serum, high	146 – 150 mEq/L<br>*146 – 150 mmol/L*	151 – 154 mEq/L<br>*151 – 154 mmol/L*	155 – 159 mEq/L<br>*155 – 159 mmol/L*	≥ 160 mEq/L<br>*≥ 160 mmol/L*
Sodium, serum, low	130 – 135 mEq/L<br>*130 – 135 mmol/L*	125 – 129 mEq/L<br>*125 – 129 mmol/L*	121 – 124 mEq/L<br>*121 – 124 mmol/L*	≤ 120 mEq/L<br>*≤ 120 mmol/L*
Triglycerides (fasting)	NA	500 – 750 mg/dL<br>*5.65 – 8.48 mmol/L*	751 – 1,200 mg/dL<br>*8.49 – 13.56 mmol/L*	> 1,200 mg/dL<br>*> 13.56 mmol/L*
Uric acid	7.5 – 10.0 mg/dL<br>*0.45 – 0.59 mmol/L*	10.1 – 12.0 mg/dL<br>*0.60 – 0.71 mmol/L*	12.1 – 15.0 mg/dL<br>*0.72 – 0.89 mmol/L*	> 15.0 mg/dL<br>*> 0.89 mmol/L*

* Values are for term infants.

† Use age and sex appropriate values (e.g., bilirubin), including preterm infants.

LABORATORY					
**PARAMETER**		**GRADE 1 MILD**	**GRADE 2 MODERATE**	**GRADE 3 SEVERE**	**GRADE 4 POTENTIALLY LIFE-THREATENING**
**URINALYSIS**		***Standard International Units are listed in italics***			
Hematuria (microscopic)		6 – 10 RBC/HPF	> 10 RBC/HPF	Gross, with or without clots OR with RBC casts	Transfusion indicated
Proteinuria, random collection		1 +	2 – 3 +	4 +	NA
Proteinuria, 24 hour collection					
	**Adult and Pediatric ≥ 10 years**	200 – 999 mg/24 h<br>*0.200 – 0.999 g/d*	1,000 – 1,999 mg/24 h<br>*1.000 – 1.999 g/d*	2,000 – 3,500 mg/24 h<br>*2.000 – 3.500 g/d*	> 3,500 mg/24 h<br>*> 3.500 g/d*
	**Pediatric > 3 mo - < 10 years**	201 – 499 mg/m^2/24 h<br>*0.201 – 0.499 g/d*	500 – 799 mg/m^2/24 h<br>*0.500 – 0.799 g/d*	800 – 1,000 mg/m^2/24 h<br>*0.800 – 1.000 g/d*	> 1,000 mg/ m^2/24 h<br>*> 1.000 g/d*

[*] Values are for term infants.

[†] Use age and sex appropriate values (e.g., bilirubin), including preterm infants.
